# Building of EMR Tools to Support Quality and Research in a Memory Disorders Clinic

**DOI:** 10.3389/fneur.2019.00161

**Published:** 2019-03-07

**Authors:** Kelly Claire Simon, Chad Yucus, James Castle, Richard Chesis, Rebekah Lai, Laura Hillman, Samuel Tideman, Lisette Garduno, Steven Meyers, Roberta Frigerio, Demetrius M. Maraganore

**Affiliations:** ^1^Department of Neurology, NorthShore University HealthSystem, Evanston, IL, United States; ^2^Health Information Technology, NorthShore University HealthSystem, Evanston, IL, United States

**Keywords:** quality improvement, cohort studies, electronic health records, data collection, research, neurology, memory disorders

## Abstract

The electronic medical record (EMR) presents an opportunity to standardize patient data collection based on quality guidelines and conduct practice-based research. We describe the development of a customized EMR “toolkit” that standardizes patient data collection with hundreds of discrete fields that supports Best Practices for treating patients with memory disorders. The toolkit also supports practice-based research. We describe the design and successful implementation of a customized EMR toolkit to support Best Practices in the care of patients with memory disorders. We discuss applications, including quality improvement projects and current research initiatives, using the toolkit. This toolkit is being shared with other departments of Neurology as part of the Neurology Practice-Based Research Network. Data collection is ongoing, including longitudinal follow-up. This toolkit will generate data that will allow for descriptive and hypothesis driven research as well-quality improvement among patients seen in a memory clinic.

## Introduction

The prevalence of dementia worldwide in 2015 was estimated to be 47 million people and is projected to increase to 132 million by 2050. The financial burden is substantial, with the cost worldwide expected to grow to 2 trillion by 2030 (1). Alzheimer's disease (AD) is the most common form of dementia, affecting more than 5 million Americans (2). With the aging population of the US, there is likely to be a significant increase in the number of individuals living with AD. Projections suggest this number could grow to 16 million by 2050 (1), representing a more than three-fold increase in prevalence. In addition to considerable financial costs, dementia is a significant burden to patients, families, and caregivers.

Dementia represents the end of a spectrum of pathological changes resulting in significant impairment of memory, learning, thinking, language and judgment. MCI is an intermediate between normal age-related cognitive changes and dementia (3) and is categorized as either amnestic or non-amnestic (4). Further, these are subdivided into a single domain or multi-domain depending on the types of deficits present (3). Amnestic type is a strong prognostic factor for the subsequent development of AD. Prevalence estimates are challenging to ascertain for a variety of reasons, but in the population-based Mayo Clinic Study of Aging, the prevalence of MCI among non-demented individuals 70 and older was 16% (5), and the incidence rate was 68/1,000 person-years (6).

Delaying progression is crucial as dementia imposes a substantial burden beyond that of MCI. As such, early identification and intervention in individuals with MCI is of benefit for reducing future disease burden. Notably, patients with MCI and AD have an increased risk of behavioral and psychiatric symptoms (7–10). It has been estimated that up to 90% of dementia patients will experience these symptoms at some point during their disease (11, 12), and the presence of these is associated with worse outcomes, increased caregiver burden, longer hospitalizations and increased risk of medication misuse (13–18). Therefore, identifying these is crucial to providing early intervention and decreasing the risk of injury or worsening symptoms. Given the burden of dementia and the overall prevalence, even a small reduction in disease burden or small increase in symptom management could have an important population-level impact.

Providing care for dementia patients is complex and is often time-consuming for a thorough evaluation. Patients are frequently experiencing cognitive changes involving memory, thinking, language and judgment. Also, they may require a caregiver or proxy to conduct a thorough history. In a recent qualitative study, Jennings et al. found that patient and caregiver health goals change throughout the disease (19), emphasizing the importance of frequent re-assessment and ongoing care management. AAN guidelines have been published to address quality measures for patients with dementia (20–24), and more recently for MCI (25). Given the high need for intervention and support services for patients and their caregivers, integrating these Best Practices into routine clinical care represents optimal care. The degree, however, to which guidelines are implemented in practice and how frequently they are adhered to, is unclear.

Traditionally, electronic medical information has been entered in a non-standardized manner, frequently as free text. This makes data extraction challenging and hinders the ability to assess quality measures and conduct quality improvement initiatives and practice-based research. The electronic medical record (EMR) presents an opportunity to address these issues by standardizing care with discrete data collection. We have developed an EMR (Epic) “toolkit” that is customized to care of MCI/dementia patients to support Best Practices. The toolkit collects hundreds of fields of discrete data and includes progress notes with simple mouse clicks. Additionally, the toolkit supports practice-based research, at the point of care.

## Methods

### Toolkit Development and Building

The Department of Neurology at NorthShore University HealthSystem (NorthShore), located in the northern suburbs of Chicago, includes two cognitive disorder specialists practicing at four practice sites. Our seven-stage process for quality improvement and practice-based research using the electronic medical record has been previously described (26). We describe the development of our highly customized Memory SCDS “toolkit” that supports clinical evaluation at initial, annual or interval visits.

#### Content Development

Our goal was to build an EMR toolkit with the purpose of supporting Best Practices in treating patients with cognitive disorders. To achieve this goal, neurologists specializing in cognitive impairment at NorthShore held frequent meetings to discuss necessary elements to support Best Practices. This included not only specific elements but also specific instruments. A review of pertinent medical literature, AAN quality measures on Dementia (20–25) and the Alzheimer's Association Guidelines (27) was used to reach our physician consensus on the content for the toolkit.

#### Toolkit Building

After deciding on the content, we conducted meetings with programmers from NorthShore's EMR Optimization team every 2 weeks. Using our existing EMR platform (Epic), they built an SCDS toolkit that included navigators (a sidebar index of processes to choose from), electronic forms (which have the ability to auto-score and auto-interpret), and summary flow sheets. We included free text fields to allow for additional information. The content includes discretized fields to record detailed information regarding symptoms (past and current), medication history, and treatment response. Relevant imaging and imaging reports are also included. When possible, results of imaging reports are also entered as discretized fields. We also included several score test measures, including the Barthel Index (28), GDS (Geriatric Depression Score) (29), FAQ (Functional Activities Questionnaire) (30), MoCA (Montreal Cognitive Assessment) 8.1 (31), and STMS (Short Test of Mental Status) (32). These measures are auto scored (when appropriate) and provide interpretation to the clinician (for example, normal cognition vs. possible cognitive impairment). Concerning the MoCA and STMS, based on the physician's clinical judgment, one or the other may be administered depending on the particular patient. As such, we convert each to an MMSE-converted score (Mini-Mental State Examination-converted) (33). Both the MoCA and STMS can be converted to the MMSE and, thus, we use the MMSE as a standardized measure of cognitive assessment. Although there are many potentially relevant tests, these were chosen based on our physician's review of pertinent literature and clinical judgment to support their practice. We designed workflows (the order and assignment of tasks to a care team that included a medical assistant and a behavioral neurologist) and mapped items to the progress notes (the order and layout in which the content would write). Of importance was that the toolkit implementation did not extend our appointment times (60 min for initial visit). Although there is a learning curve, we have found that once physicians are familiar with the toolkit, these do not add to face-to-face physician time. However, because time is important, as discussed later, we continually evaluate the toolkits for opportunities to reduce time without compromising elements of Best Practices.

#### Toolkit Implementation

After the SCDS toolkit build was complete, the implementation phase began. We first used the toolkit in a development environment, to test the usability and allow physicians the opportunity to provide feedback on the toolkit flow and any potential issues with usage. Once all users were satisfied with the toolkits performance, it was moved to production. We continued to meet bi-weekly to discuss any new issues or potential opportunities to refine the toolkit.

#### Quality Monitoring

Following implementation, we continued to meet every 2 weeks with programmers specialized in extracting, transforming, and loading data from the EMR's data repository to specific data marts in NorthShore's Enterprise Data Warehouse (EDW). The EDW programmers created enrollment reports for tracking patients and produced data quality reports indicating which required data was missing from office visits. These data quality reports are distributed to the care team monthly. Physicians and other care team members have the opportunity to review missing data to determine the cause. When systematic errors occurred, the teams had the opportunity to improve their use of the toolkits or to request optimizations or a change in data requirements. Because the toolkit content was determined through a physician-led process, frequently missing data is likely due to a measure not to be testable by the nurse or the physicians. For this reason we have included “not tested” or “unable to test” options for tests to distinguish this from missing data when appropriate. If the physicians are not using an element, it presents an opportunity to make changes to the toolkit. The monthly reports produced only a few or no data checks per provider once the project was established. However, toolkit optimization is ongoing and providers have the opportunity to request changes as practice standards and diagnostics evolve continually. We also produce monthly de-identified descriptive reports of the data generated from patient encounters. Our research team reviews these for inconsistencies or errors.

### Application

**Figure 1:**
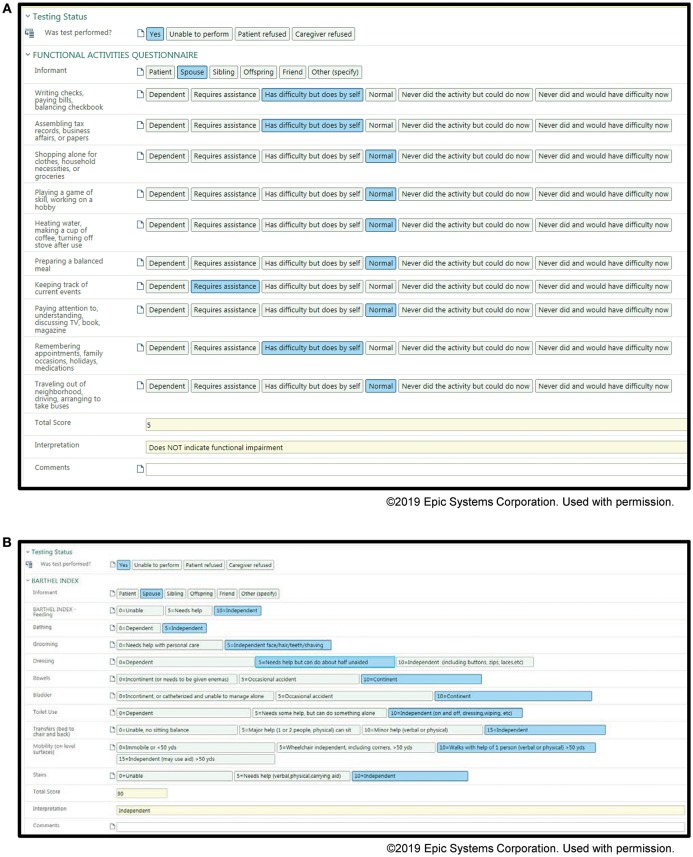
Screenshots of the Memory Disorders SDCS toolkit within the EMR. **(A)** Functional activities questionnaire, **(B)** Barthel Index.

**Figure 2:**
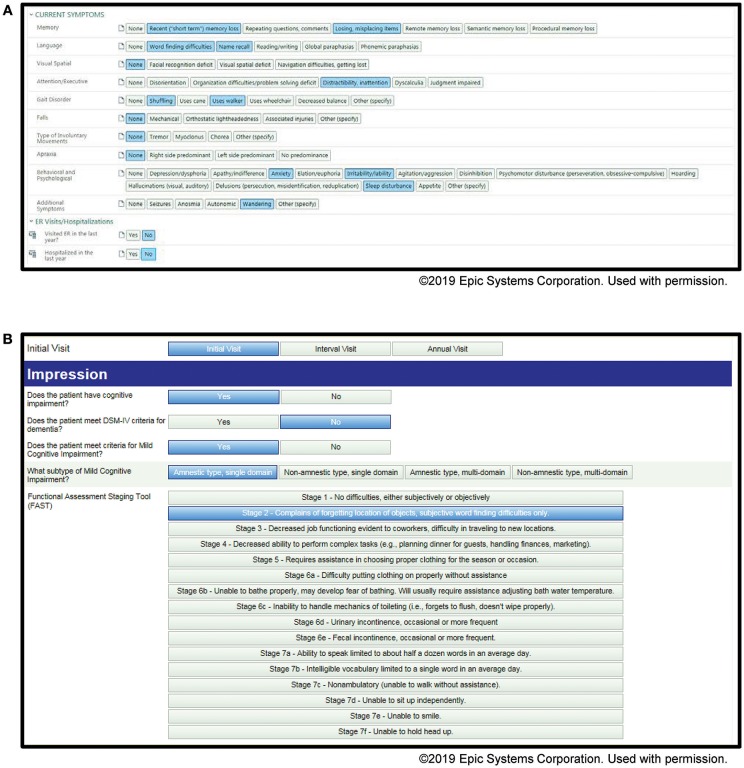
Screenshots of the Memory Disorders SDCS toolkit within the EMR. **(A)** Current symptoms, **(B)** initial visit impression.

Examples of our screenshots from our toolkits are shown in Figures 1, 2. From the toolkits, we have productionalized de-identified descriptive reports. These reports are run monthly and provide visual displays of the data. For categorical variables, histograms with raw counts are presented (see Data Sheet 1 for full descriptive report). For continuous variables, this includes scatter plots as well as measures of central tendency and dispersion. Lastly, our productionalized reports include correlation plots and principal components analysis to examine the relationship between our continuous measures (see Data Sheet 2 for full analytical report). In addition to being of interest in understanding our patient population, these reports present opportunities for refinement of the toolkit. For example, if a variable has an extremely low frequency, we can consider whether it should be removed from the toolkit.

Similarly, if we find two measures are highly correlated, we can consider whether both need to be included. Additionally, these reports allow us to examine the data for expected relationships and to understand our patient population. For example, we can stratify these reports according to degree of impairment, producing one for patients with MCI and one for patients with dementia, for comparison.

We are also using the toolkit to identify quality improvement opportunities at the point-of-care, allowing the physician to take immediate action. As a department based initiative, we have developed Best Practice Advisories (BPAs) that alert the neurologist when a patient screens positive for depression and anxiety. When alerted, the neurologist must provide information on whether action was taken (referral, medication prescribed, etc.) or not. If not, a reason must be provided from a drop-down menu. We plan to evaluate the impact of our depression BPA by assessing clinical measures of depression and quality of life, before and after the implementation of this BPA. We are also planning similar BPAs specific to patients in our memory clinic. For example, when a patient's FAQ suggests difficulty with financial management tasks, we plan to assess the frequency that a social worker referral is ordered. Also, we plan to assess how frequently a driving evaluation is ordered if a patient has a positive driving safety screen.

Similarly we could consider whether patients with evidence of cognitive disorder as evidenced by the MoCA or STMS, have neuropsychiatric testing or advanced care planning documented. Lastly, in patients reporting falls within the past year, we plan to assess the frequency with which a physical therapy referral is ordered. Once these are implemented, we can assess the effectiveness by determining whether there is a change in physician behavior and how these changes relate to patient outcomes.

To complement the clinical data, we are also using the toolkit to enroll patients in our IRB approved DNA biobanking study. We developed a BPA that is triggered if patients meet the eligibility criteria for our DNA biobanking study. The BPA prompts the consenting of the patient and subsequent enrollment at the point of care (NorthShore IRB approved study EH10-139). These patients consent to a one-time blood draw, but otherwise, no study specific visits are required. Data is completely captured within the context of the office visit through use of the toolkit. Genome-wide SNP genotyping was recently completed on these patients and will be used to complement the clinical data and conduct novel studies of biomarkers and risk assessment. Lastly, we are actively sharing this toolkit through the Neurology Practice Based Research Network (NPBRN), which we created through a grant from AHRQ. The NPBRN partner sites adopt relevant toolkits at their site for the purposes of data sharing, to benchmark performance and to conduct quality improvement initiatives and practice-based research. For information regarding joining the NPBRN, please contact the corresponding author (DMM). Lastly, we are also enrolling patients in a point-of-care clinical trial for patients with MCI using a sub-group based adaptive design (SUBA) (NorthShore IRB approved study EH14-355) (34). We are comparing the effectiveness of three nootropic drugs. Although it is a randomized trial, it is conducted in a real clinical setting, with no study specific office visits. This trial is currently in progress.

## Advantages and Limitations

We describe here our experience with creating and implementing a customized toolkit to care for patients presenting with cognitive complaints. The advantage of our approach is that it is a physician-driven process. The foundation of the toolkit is supporting Best Practices for clinical care. From this, we can conduct clinical research at the point-of-care, as described. Data extraction from clinical data is challenging because of heterogeneity in data entry. Our toolkit has the advantage of being highly discretized, collecting hundreds of fields of data at each encounter. This makes extraction, reporting and data analysis much more accessible. We also generate frequent descriptive and analytic reports providing feedback to clinicians on their patients and the utility of toolkit usage. The toolkit also streamlines the office visit by assigning tasks to different members of the care team. Standardization of care also makes longitudinal comparison easier. In the toolkit, repeated measures can be easily visualized, allowing the physician to see changes over time quickly. This also presents the opportunity for longitudinal research. We currently have almost 3 years of longitudinal data on these patients that can be examined for quality improvement and practice based-research studies. Lastly, the standardization of data allows for collaborative research initiatives through data sharing. Multi-site research is often challenging because of differences in data collection. Standardized data collection allows the opportunity to assess quality and research questions in diverse patient populations across geographic sites, all with comparable data.

## Conclusion

The EMR presents a novel opportunity to improve patient care through quality assessment and research initiatives. We demonstrate here the creation of a standardized EMR that we are currently using in clinical practice to conduct quality initiatives and practice-based research. Through these projects, we strive to identify opportunities to improve care and outcomes for patients living with cognitive disorders.

## Ethics Statement

Written informed consent was not obtained for assessment using the EMR toolkits, as this was done in the course of routine clinical practice. Written informed consent was obtained for the extraction and storage of DNA, as part of a separate study. The DNA collection protocol was approved by the NorthShore University HealthSystem Institutional Review Board.

## Author Contributions

SM, RF, and DM developed the toolkit and contributed conception and design of the study. CY contributed data and study conception and design. Other data contributors are JC and LG. RC, RL, and LH also developed the toolkit. ST contributed statistical support. KS wrote the first draft of the manuscript. All authors contributed to manuscript revision, read, and approved the submitted version.

### Conflict of Interest Statement

The authors declare that the research was conducted in the absence of any commercial or financial relationships that could be construed as a potential conflict of interest.
