# Supplementary material for: Building of EMR Tools to Support Quality and Research in a Memory Disorders Clinic
Source: Front Neurol. 2019 Mar 7;10:161. doi: 10.3389/fneur.2019.00161 (PMC6416163; doi:10.3389/fneur.2019.00161)
Supplement: Supplementary file 1 [file Data_Sheet_1.PDF]

Memory

Descriptive Report

2018-04-01

- Gender
- Race
- BMI
- Years of Education
- Tobacco User
- Alcohol User
- Age at encounter
- Age at Onset
- Disease Duration
- Initial Symptoms
- Current Symptoms
- Current Language Symptoms
- Current Visual Spatial Symptoms
- Current Executive Symptoms
- Current Gait Disorder Symptoms
- Current Fall Symptoms
- Current Involuntary Movements Symptoms
- Current Apraxia Symptoms
- Current Behavioral Symptoms
- Current Additional Symptoms
- Current Rx
- Prior Rx
- Live at Location
- Caregiver
- CT Head
- MRI Brain
- PET
- EEG
- Neuropsych
- Cognitive Impairment Impression
- DSM-IV Criteria for Dementia Impression
- Dementia Cause
- Criteria for Mild Cognitive Impairment
- Mild Cognitive Impairment Subtype
- Functional ASMT Staging Tool
- Barthel Interpretation
- FAQ Interpretation
- GDS Interpretation
- MMSE Interpretation
- Family History of ALS
- Family Members with History of ALS

- Family History of Epilepsy
- Family Members with History of Epilepsy
- Family History of Dementia
- Family Members with History of Dementia
- Family History of Parkinsons
- Family Members with History of Parkinsons
- Family History of Stroke
- Family Members with History of Stroke

Total Patients = 1956

Encounters restricted to the following visit types: Initial Visit

---

Gender

Gender	Count	Percent
Female	1140	58.3
Male	816	41.7

Race

Value	Percent	Count	Male	Female
NA	57.2	1118	403	715
Caucasian	39.3	769	385	384
Asian	1.6	32	15	17
African American	1.1	21	11	10
Hispanic/Latino	0.8	16	2	14

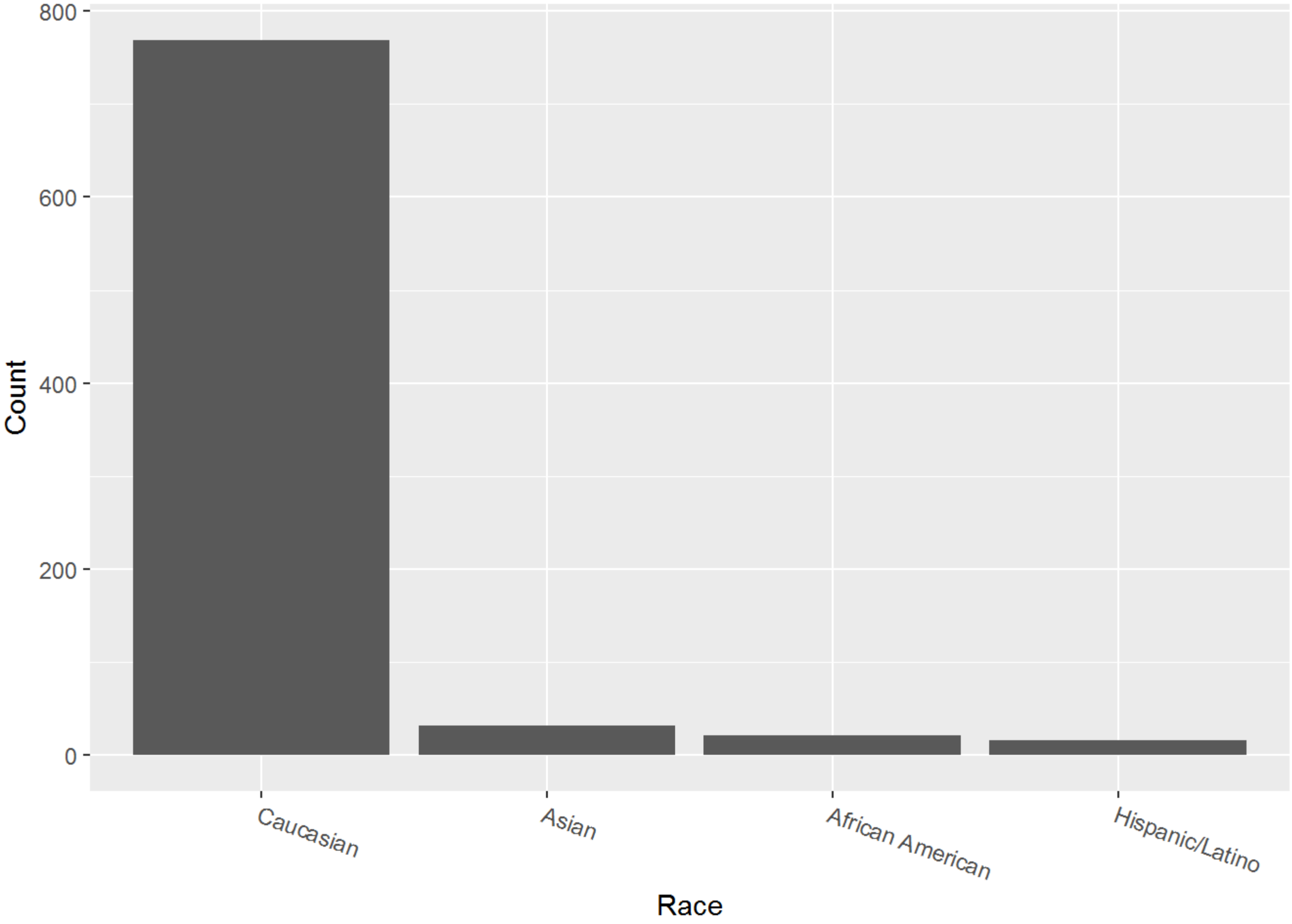

BMI

Min	Median	Mean	Max	St.Dev	No.Data
14	25	25.7	50	4.861	162

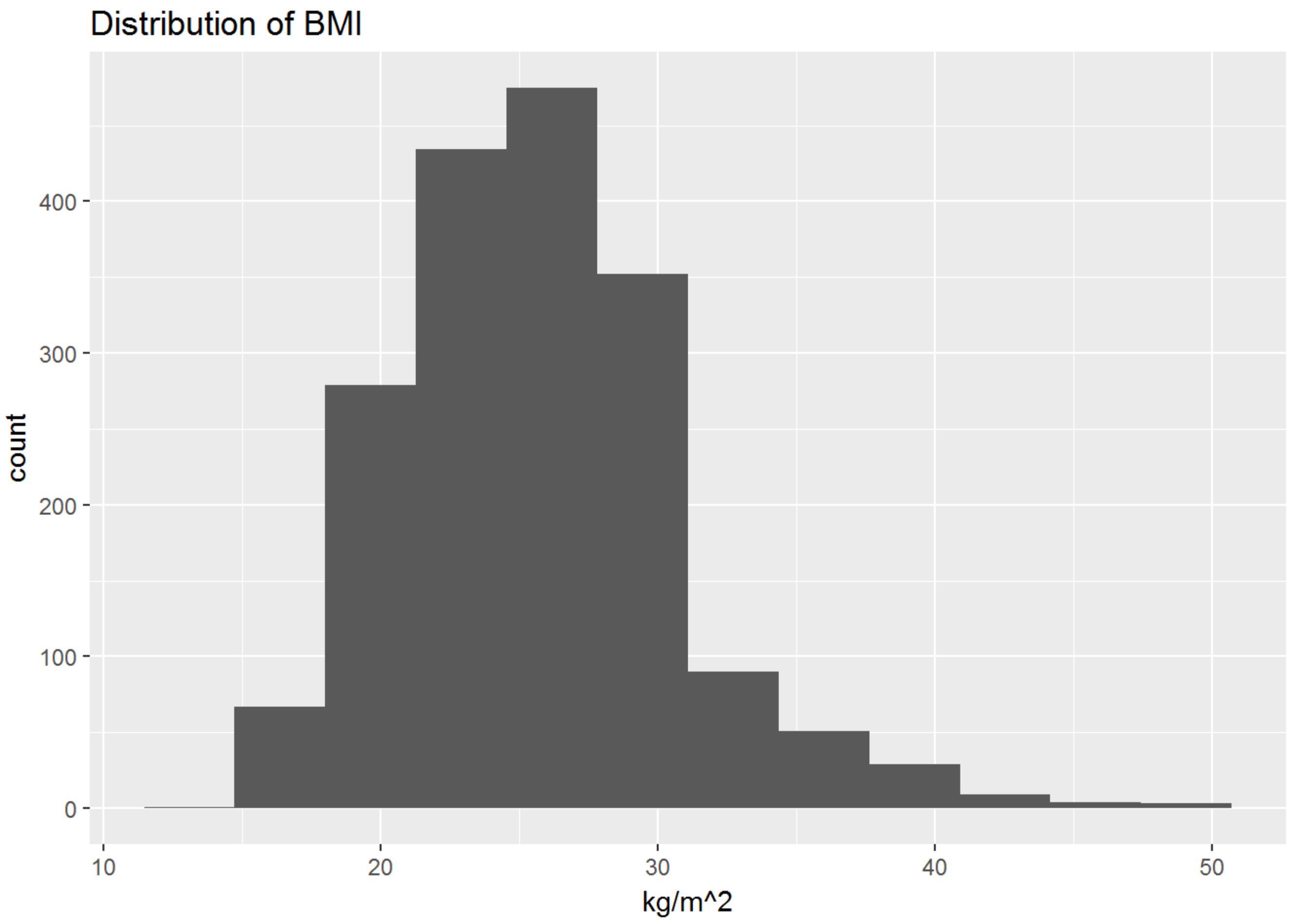

Distribution of BMI
count
400
300
200
100
0
10
20
30
40
50
kg/m^2

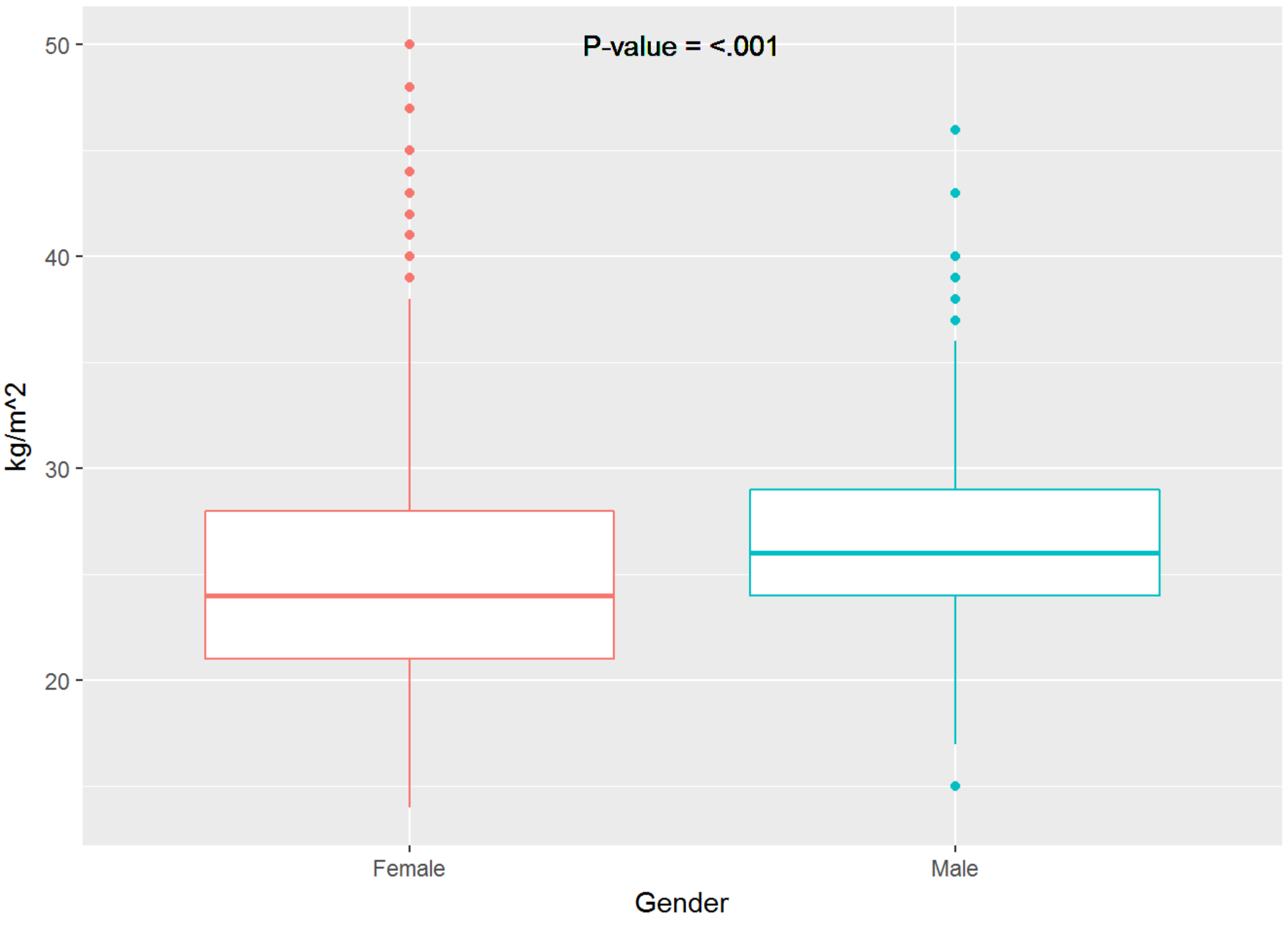

P-value = <.001
50
40
30
20
kg/m^2
Female
Male
Gender

Years of Education

Min	Median	Mean	Max	St.Dev	No.Data
0	15	14.81	30	3.691	37

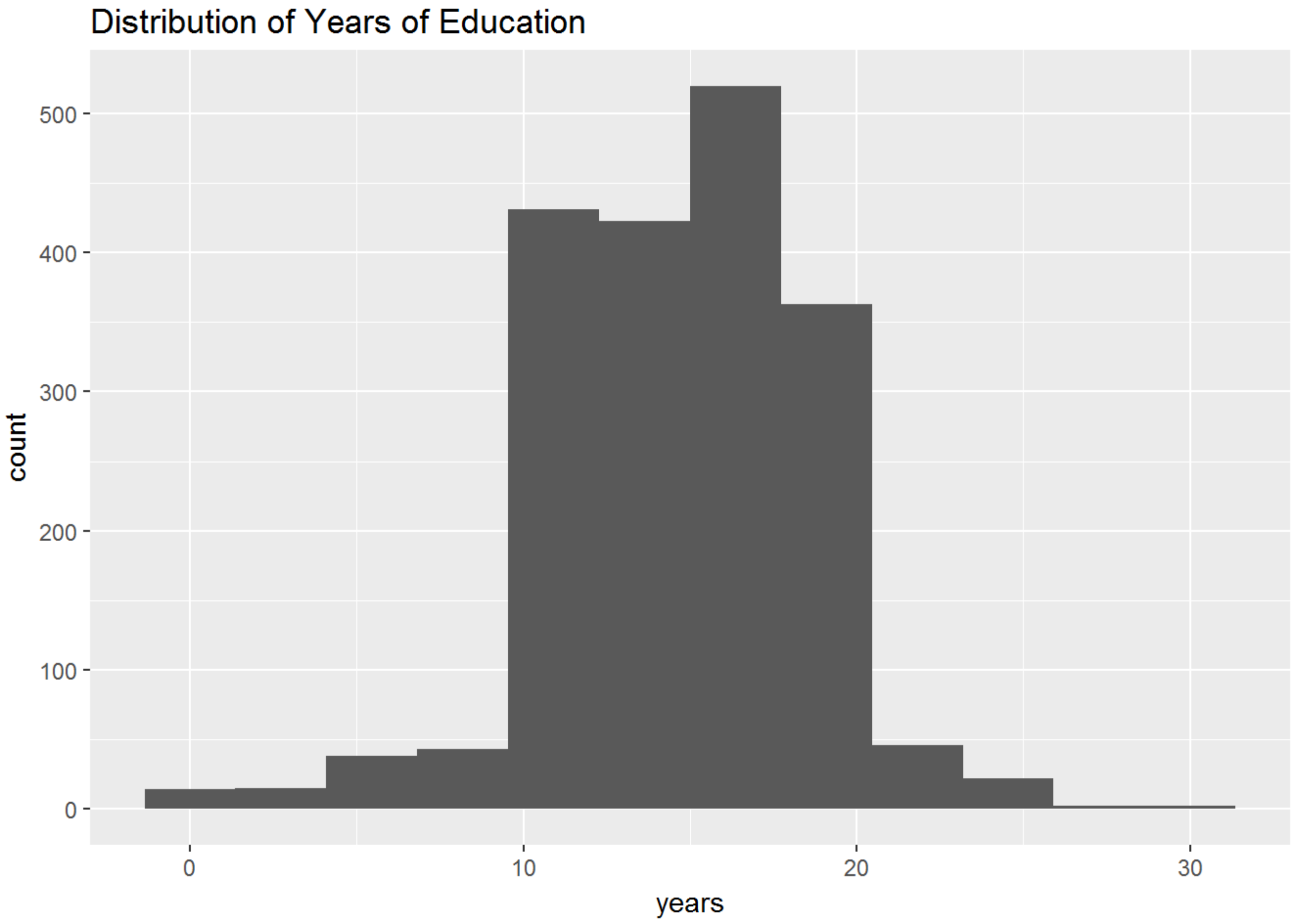

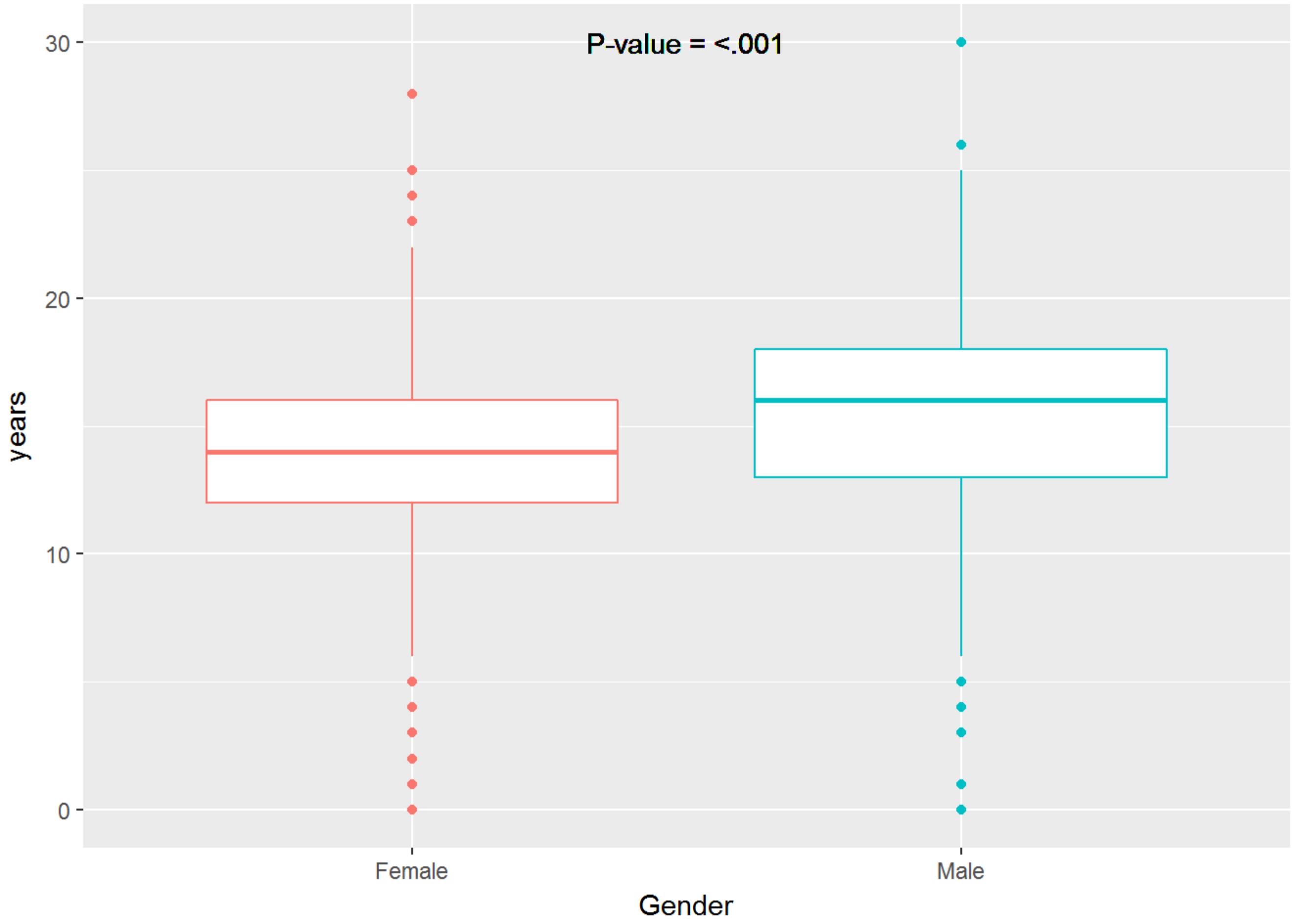

Tobacco User

Value	Percent	Count	Male	Female
Never	50.2	981	338	643
Quit	46.8	915	450	465
Yes	2.9	56	26	30
Not Asked	0.1	2	0	2
Passive	0.1	2	2	0

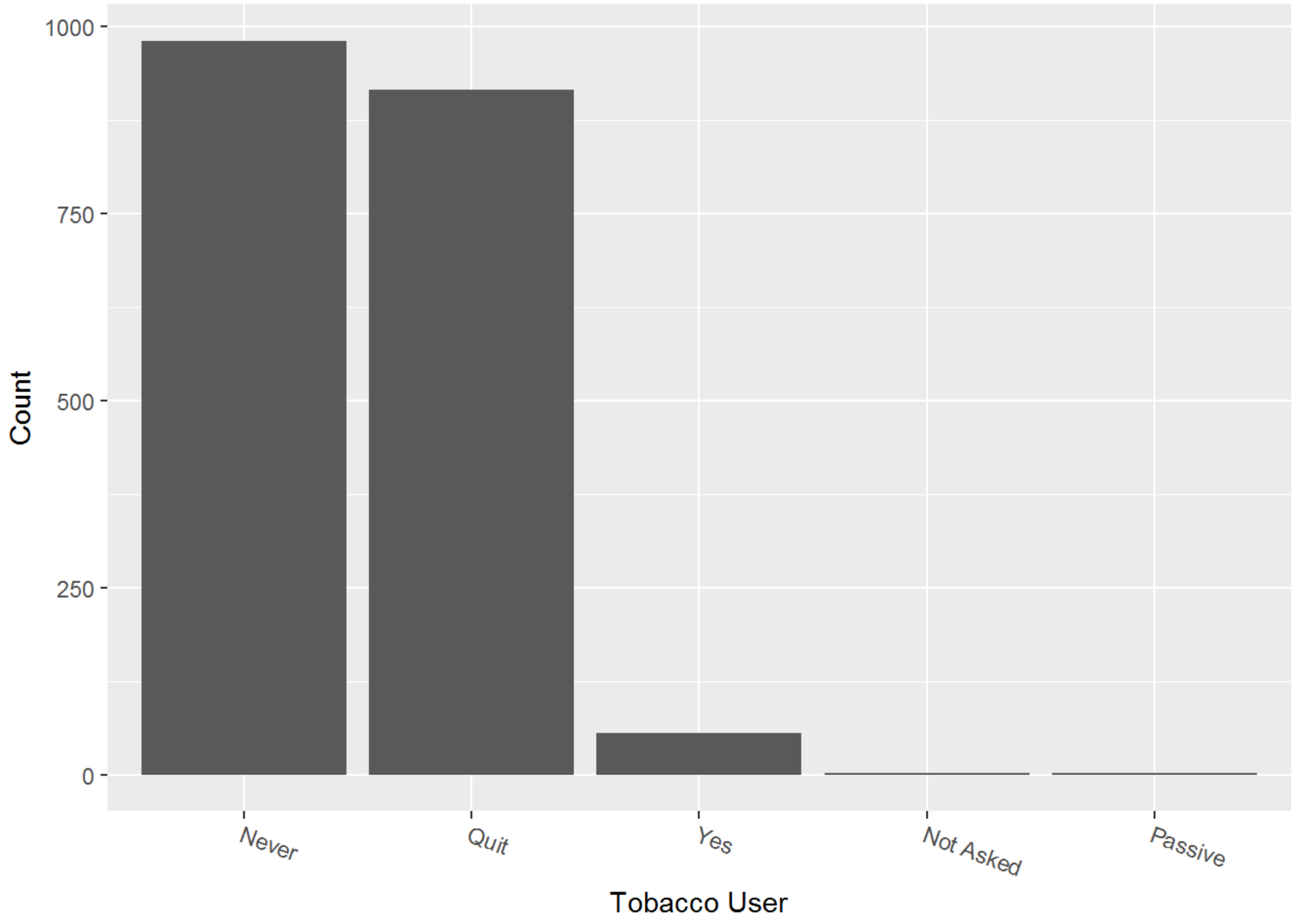

Alcohol User

Value	Percent	Count	Male	Female
No	53.6	1048	393	655
Yes	45.3	886	417	469
Not Asked	0.9	18	6	12
NA	0.2	4	0	4

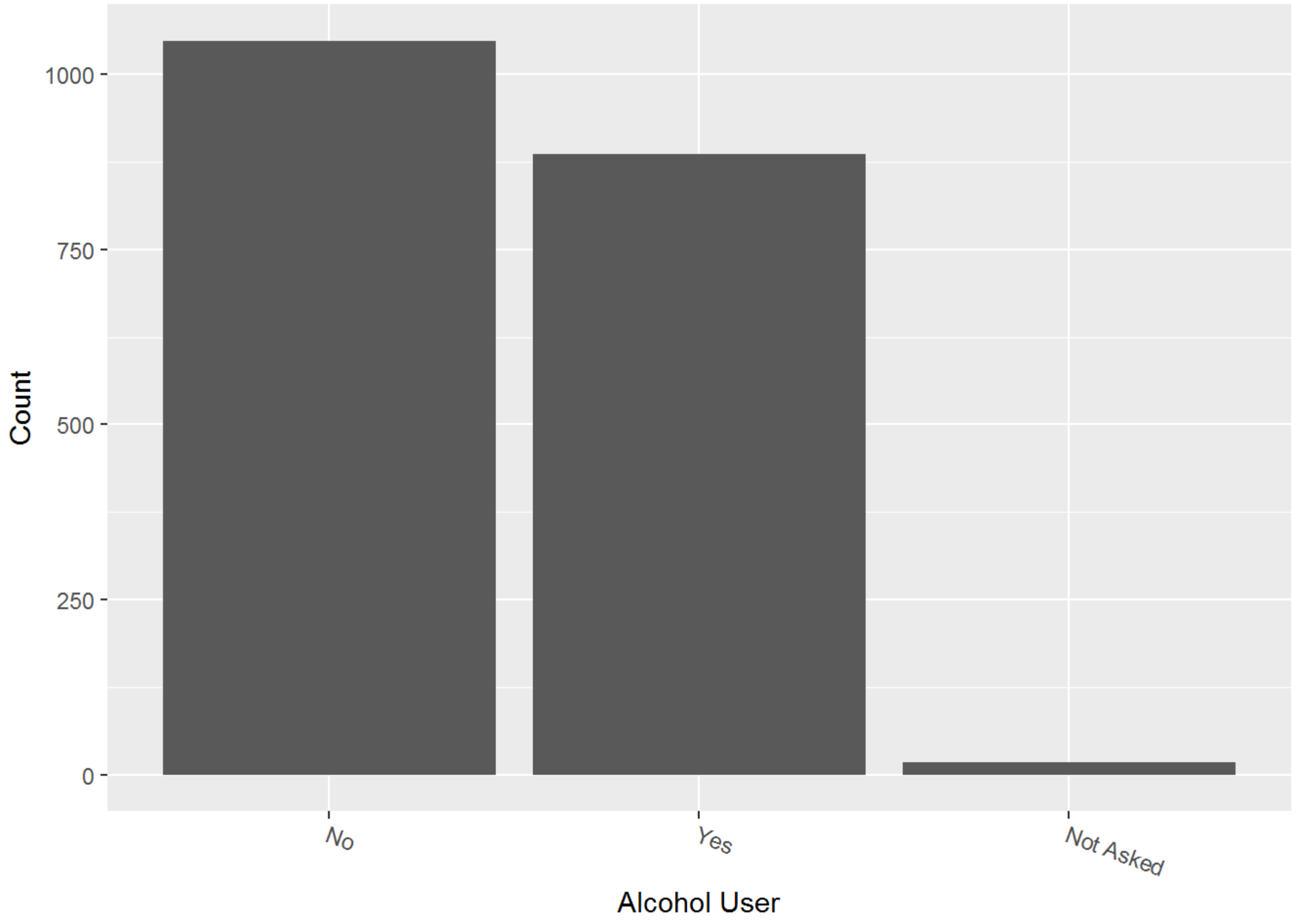

Age at encounter

Min	Median	Mean	Max	St.Dev	No.Data
34.88	80.6	79.05	98.78	8.991	0

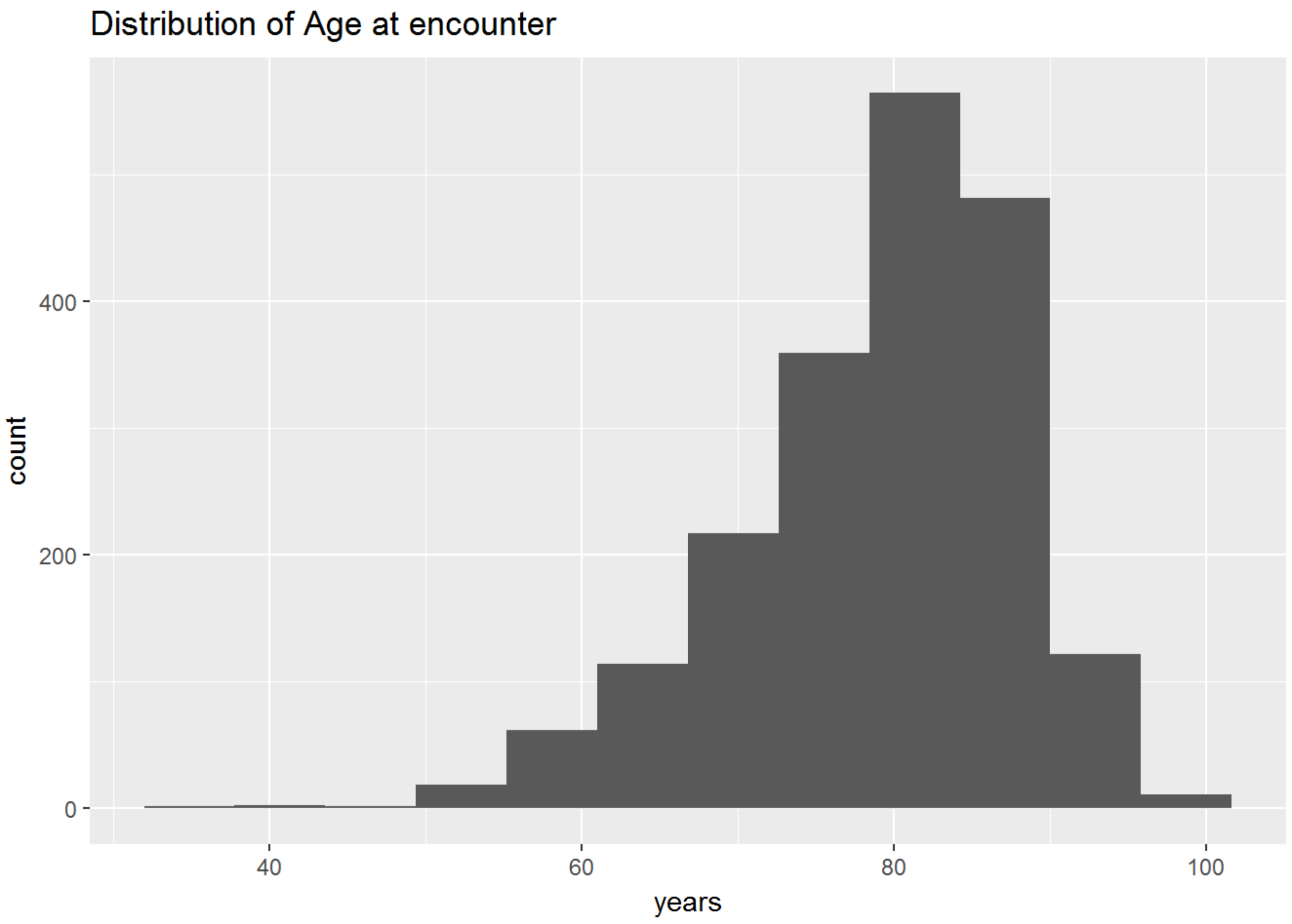
Distribution of Age at encounter
count
400
200
0
40
60
80
100
years

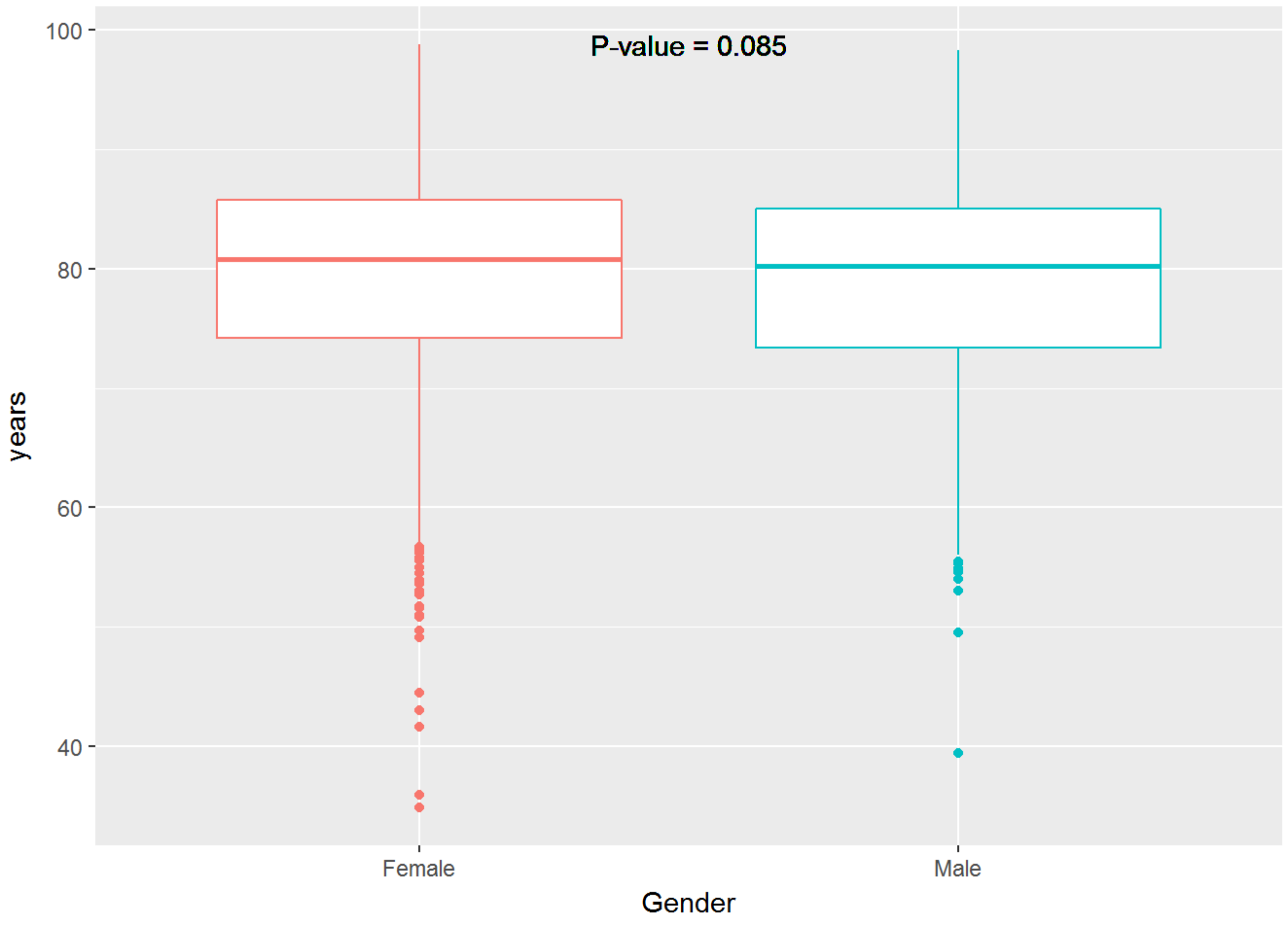
P-value = 0.085
years
100
80
60
40
Female
Male
Gender

Age at Onset

Min	Median	Mean	Max	St.Dev	No.Data
32.08	77.05	75.5	96.68	9.382	150

Distribution of Age at Onset

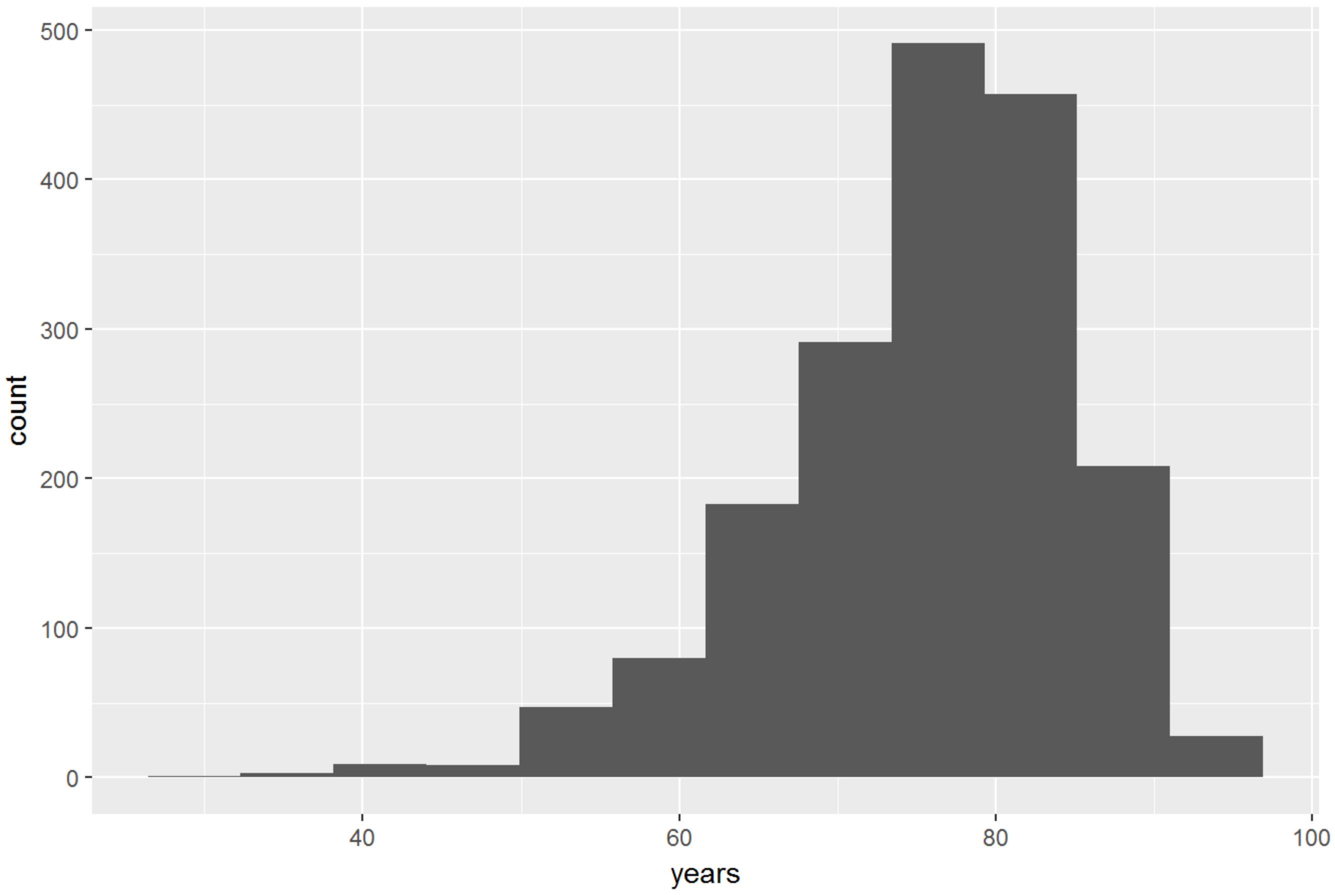

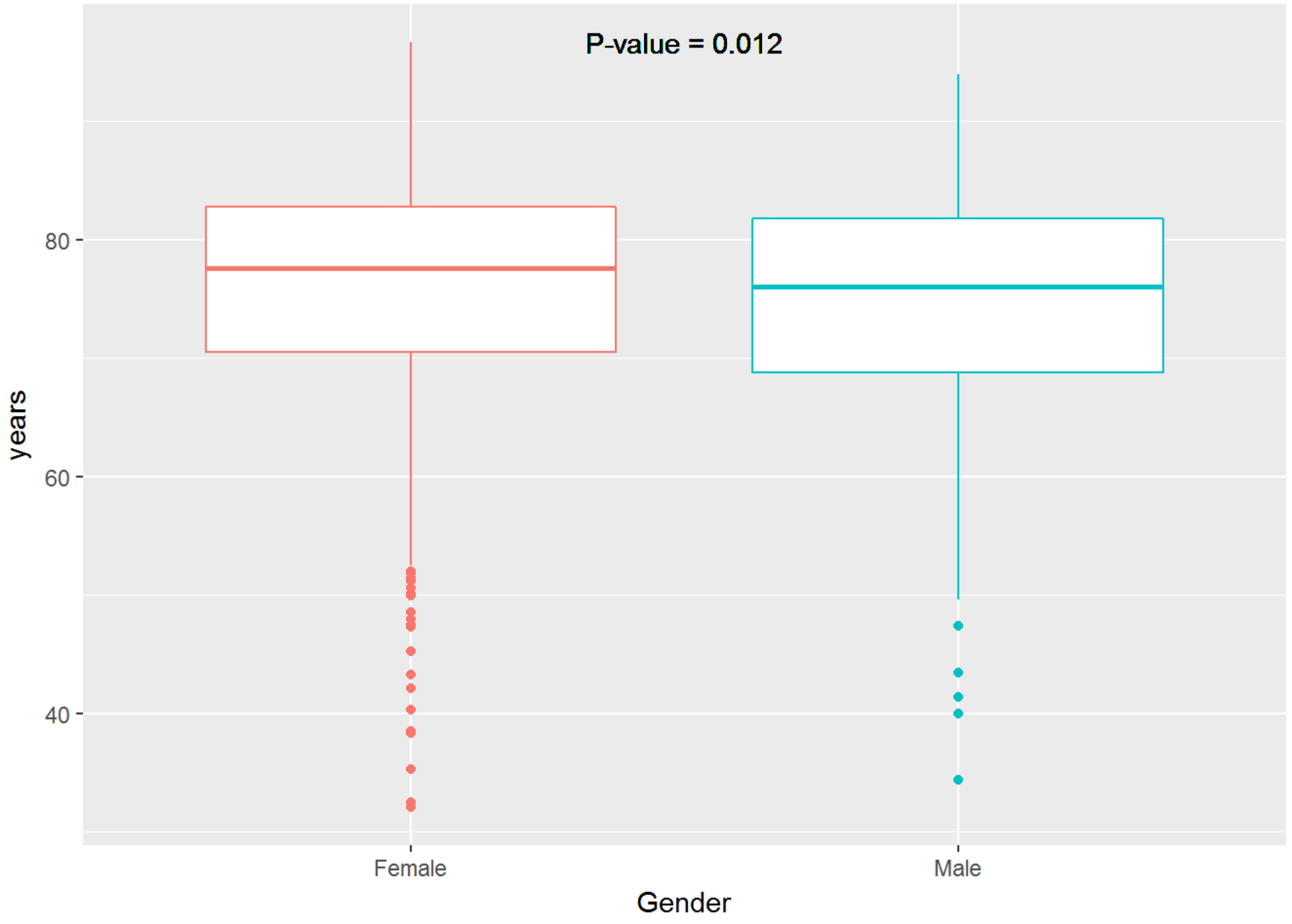

Disease Duration

Min	Median	Mean	Max	St.Dev	No.Data
0.04654	2.793	3.493	17.52	2.605	150

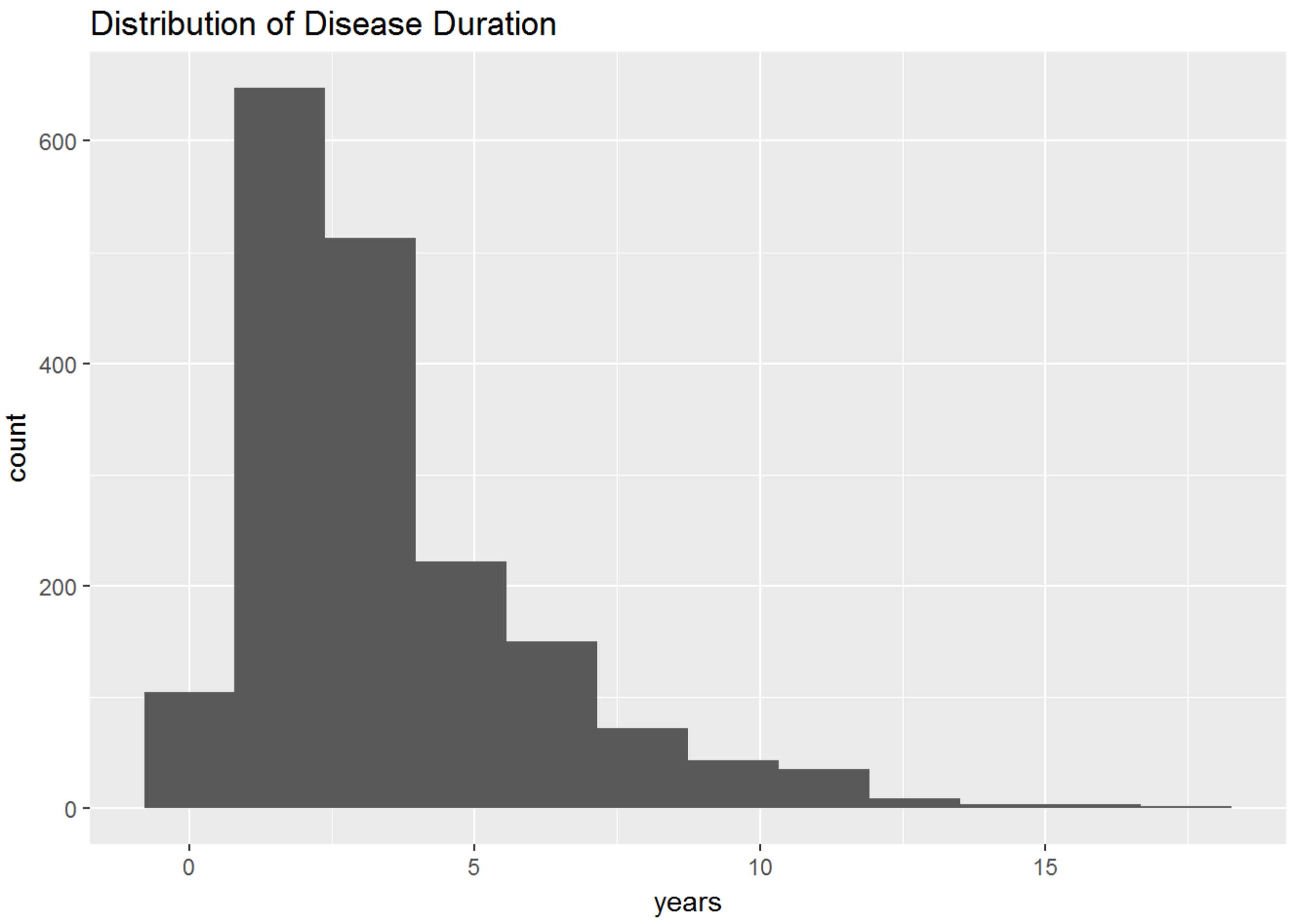
Distribution of Disease Duration
count
600
400
200
0
0
5
10
15
years

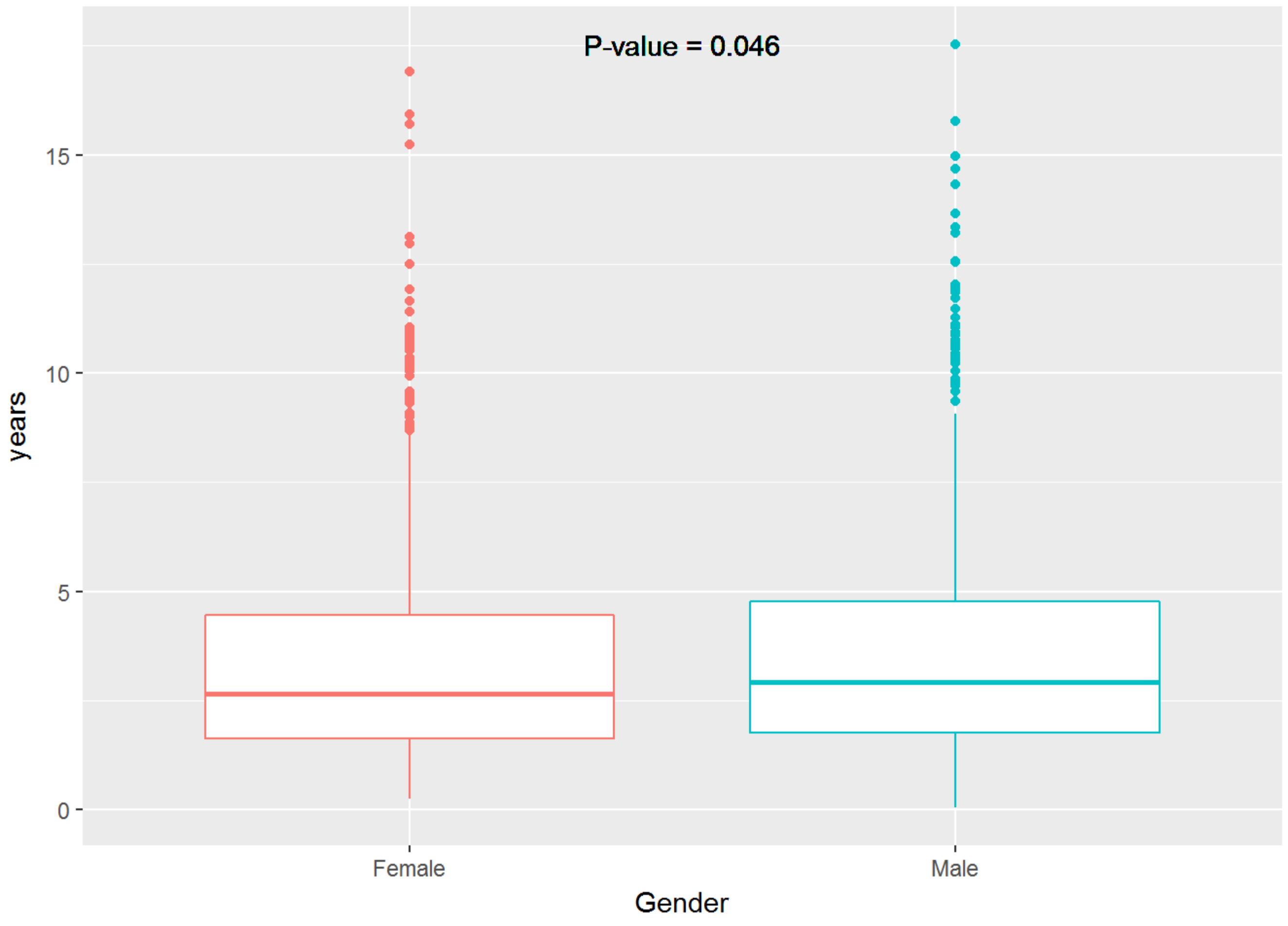
P-value = 0.046
years
15
10
5
0
Female
Male
Gender

Initial Symptoms

Value	Percent	Count	Male	Female
Memory difficulty	85.4	1671	698	973
Word finding difficulty	14.5	283	121	162
Behavioral change	5.4	106	53	53
Other cognitive difficulty	4.6	89	44	45
Gait disorder/falls	1.7	33	17	16
Other (specify)	0.5	10	6	4
Vision change	0.5	10	6	4
Urinary incontinence	0.2	4	3	1
Apraxia	0.1	2	1	1

Current Symptoms

Value	Percent	Count	Male	Female
Recent (“short term”) memory loss	93.3	1824	765	1059
Repeating questions, comments	62.3	1218	478	740
Losing, misplacing items	57.1	1116	449	667
Remote memory loss	6.9	134	55	79
Procedural memory loss	6.7	132	59	73
Semantic memory loss	5.6	110	42	68
None	3.1	61	26	35

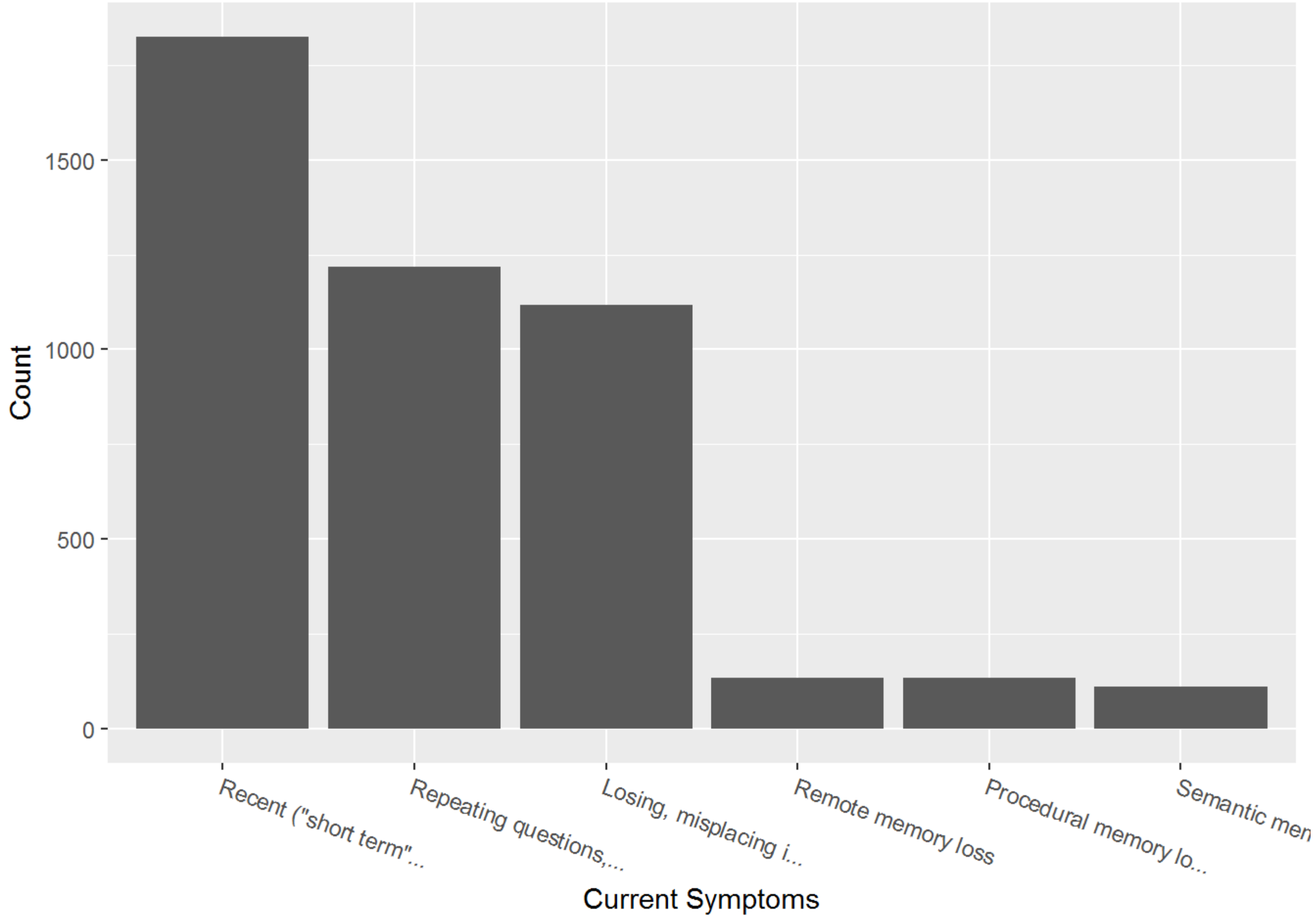

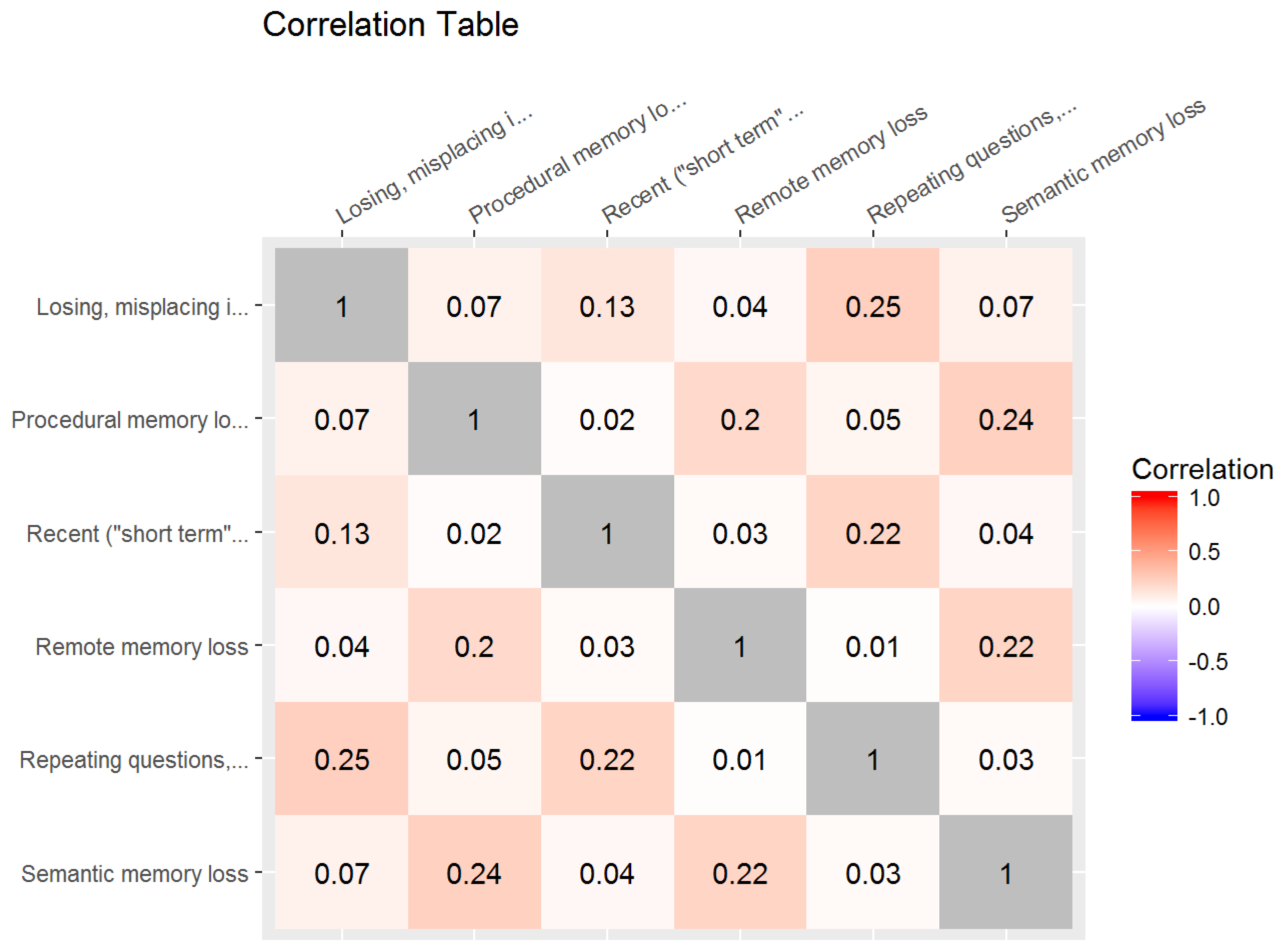

Current Language Symptoms

Value	Percent	Count	Male	Female
Word finding difficulties	47.6	932	409	523
None	41.6	814	321	493
Name recall	33.5	655	292	363
Global paraphasias	6.9	135	46	89
Reading/writing	5.2	102	52	50
Phonemic paraphasias	2.1	42	16	26

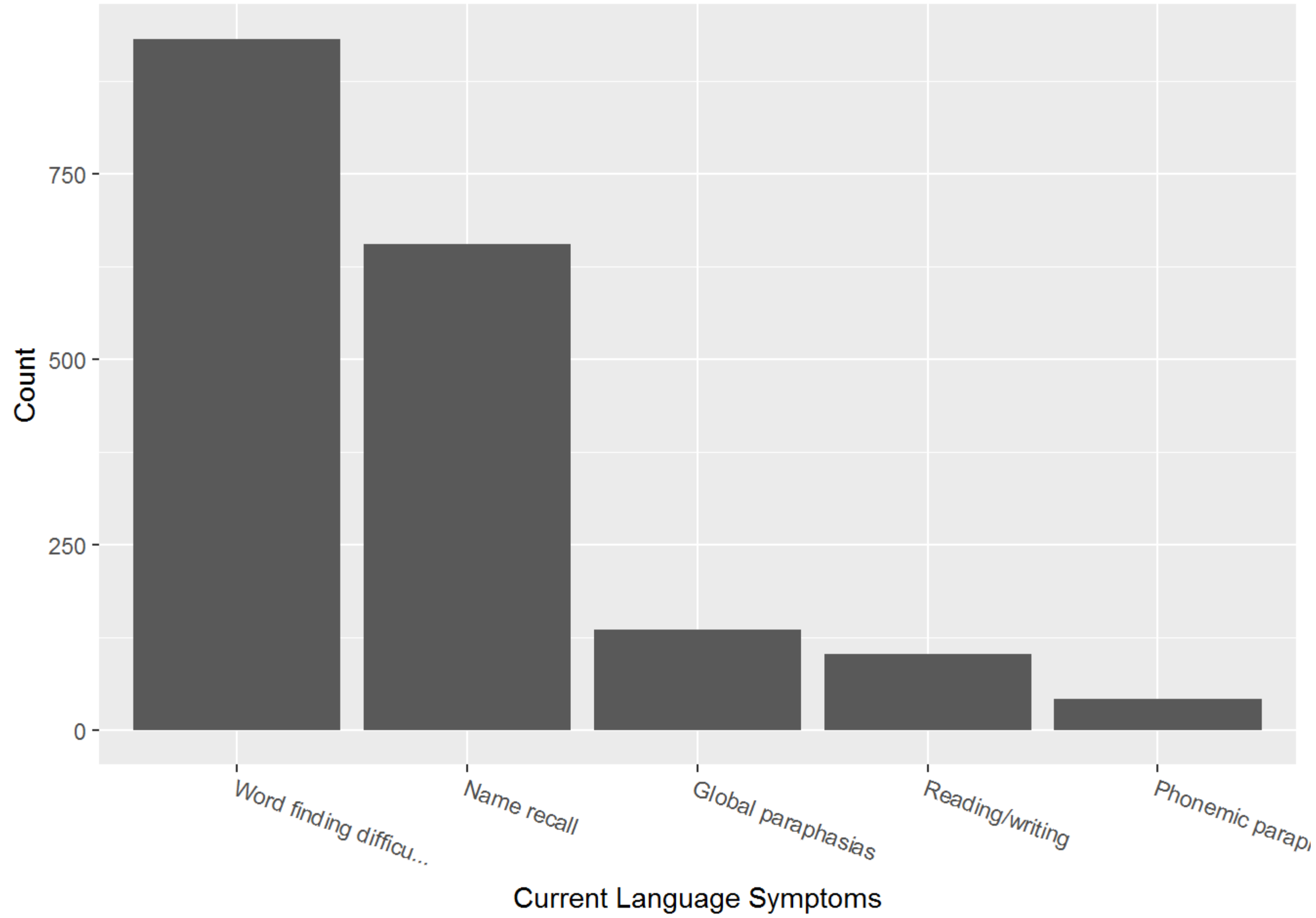
Count
750
500
250
0
Word finding difficu...
Name recall
Global paraphasias
Reading/writing
Phonemic parap
Current Language Symptoms

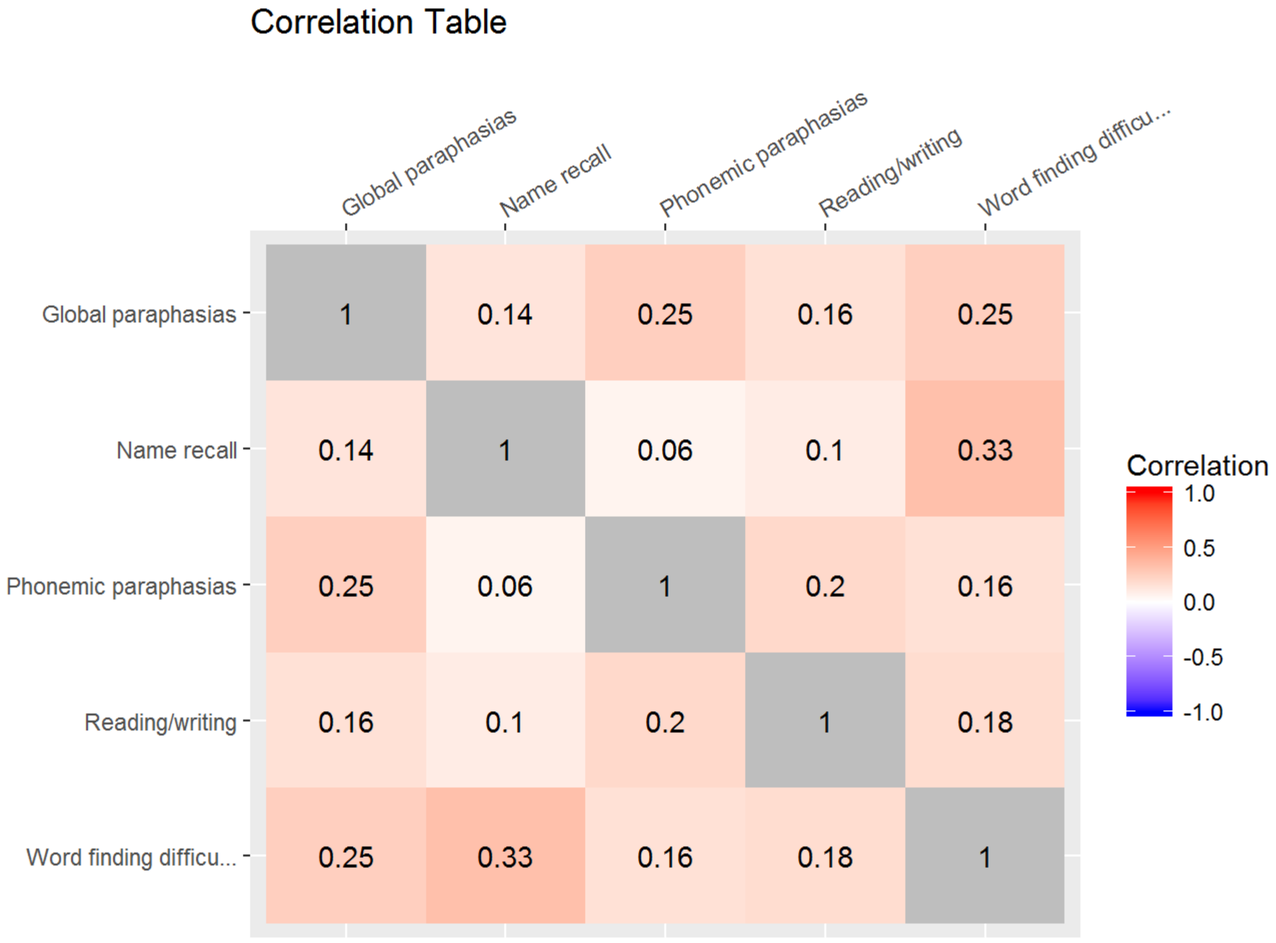
Correlation Table
Global paraphasias
Name recall
Phonemic paraphasias
Reading/writing
Word finding difficu...
1
0.14
0.25
0.16
0.25
0.14
1
0.06
0.1
0.33
0.25
0.06
1
0.2
0.16
0.16
0.1
0.2
1
0.18
0.25
0.33
0.16
0.18
1
Correlation
1.0
0.5
0.0
-0.5
-1.0

Current Visual Spatial Symptoms

Value	Percent	Count	Male	Female
None	61.7	1207	461	746
Navigation difficulties, getting lost	30.6	599	301	298
Visual spatial deficit	10.3	201	86	115
Facial recognition deficit	2.8	55	18	37

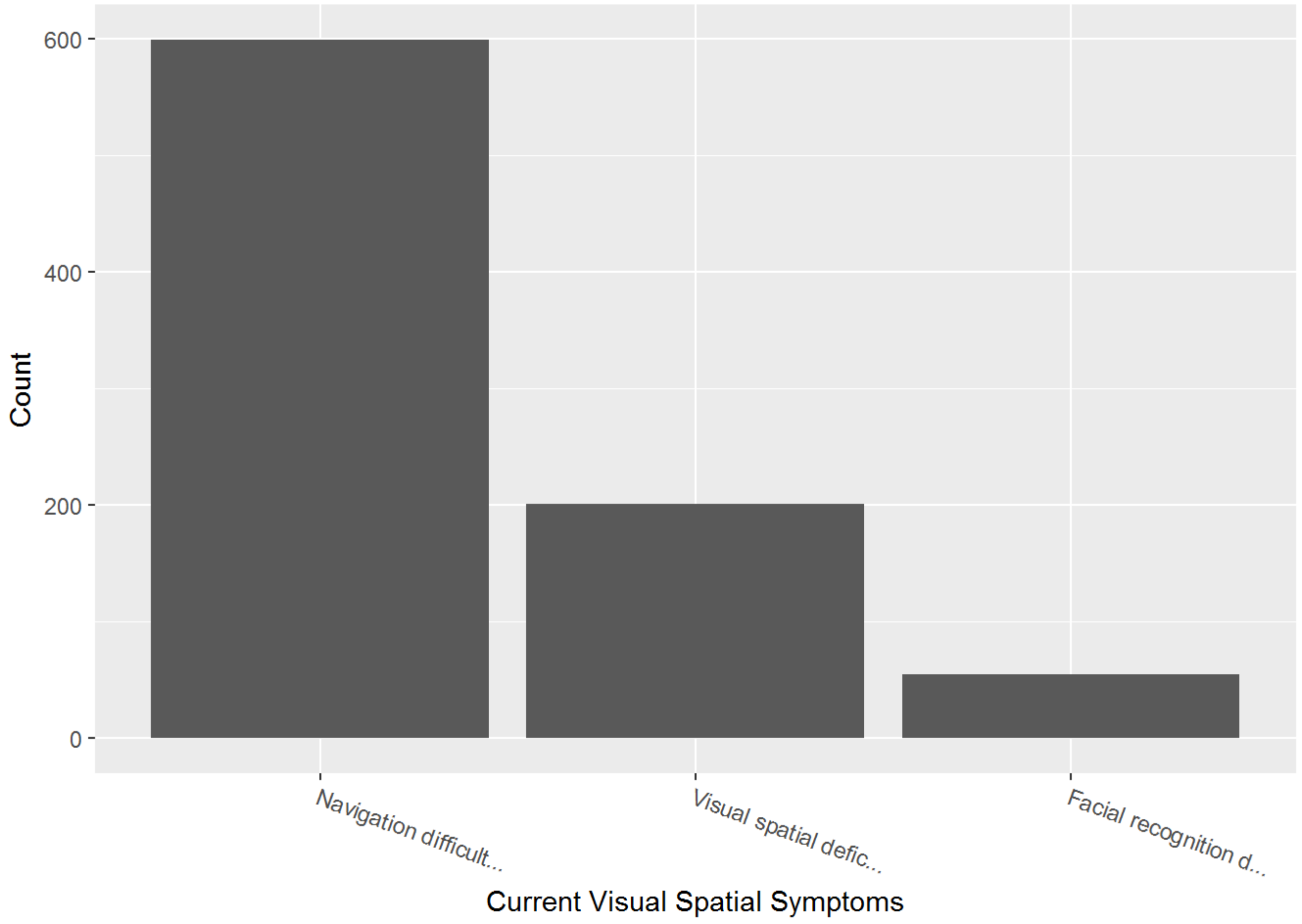

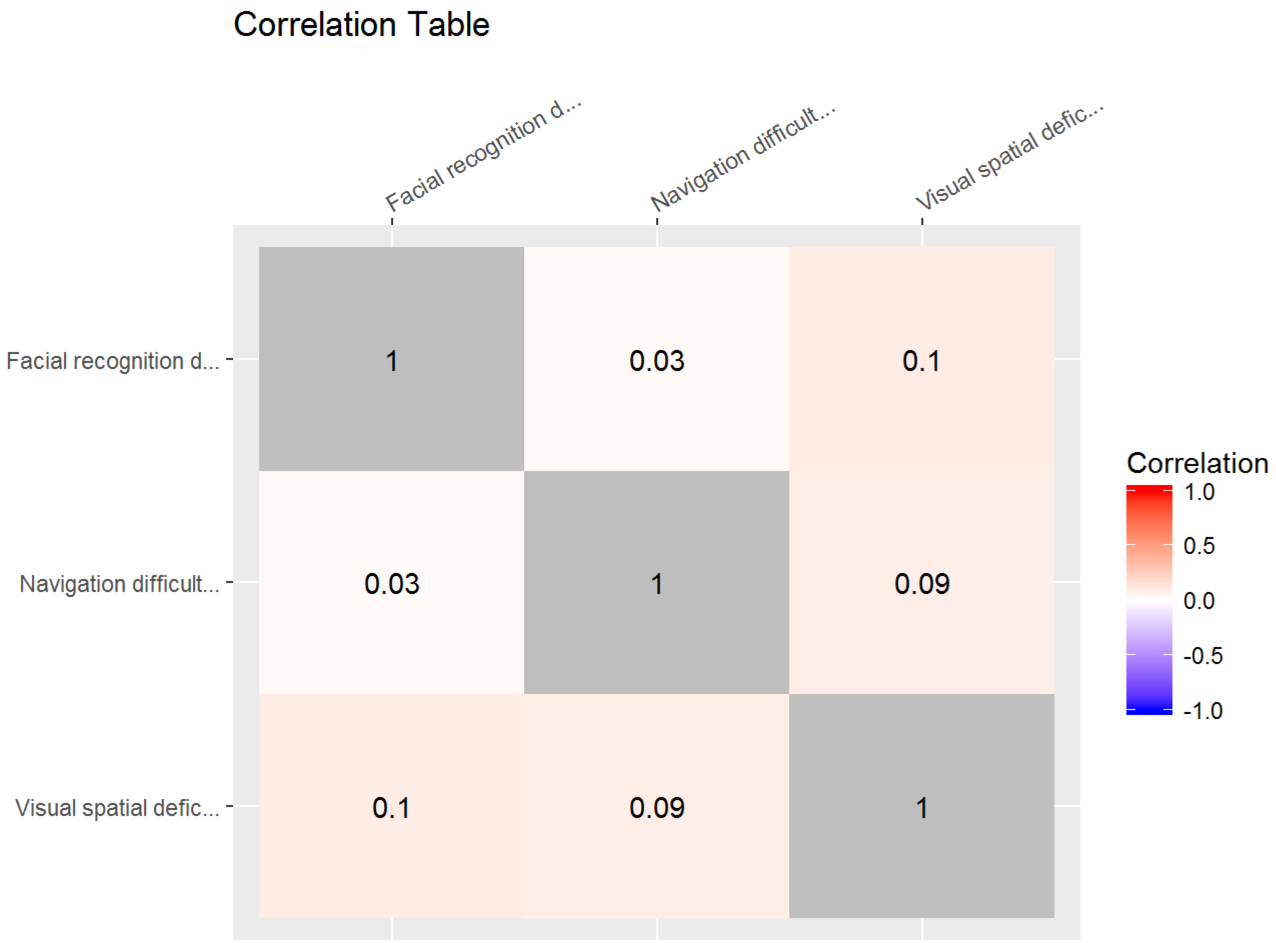

Current Executive Symptoms

Value	Percent	Count	Male	Female
Organization difficulties/problem solving deficit	57.2	1119	492	627
None	30.7	601	232	369
Distractibility, inattention	24.9	488	221	267
Disorientation	24.2	473	177	296
Judgment impaired	5	97	49	48
Dyscalculia	4.3	84	42	42

Memory

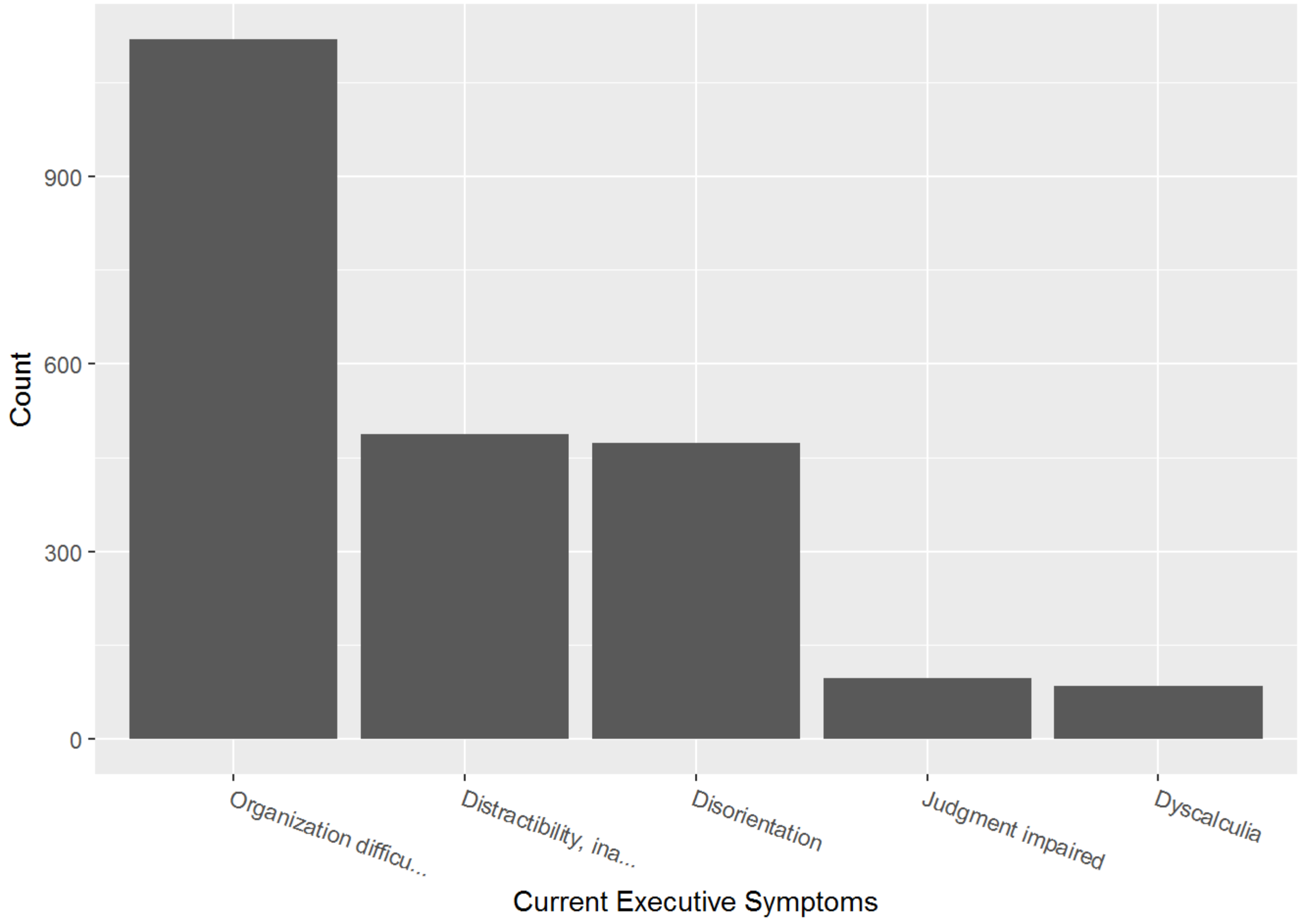

Correlation Table

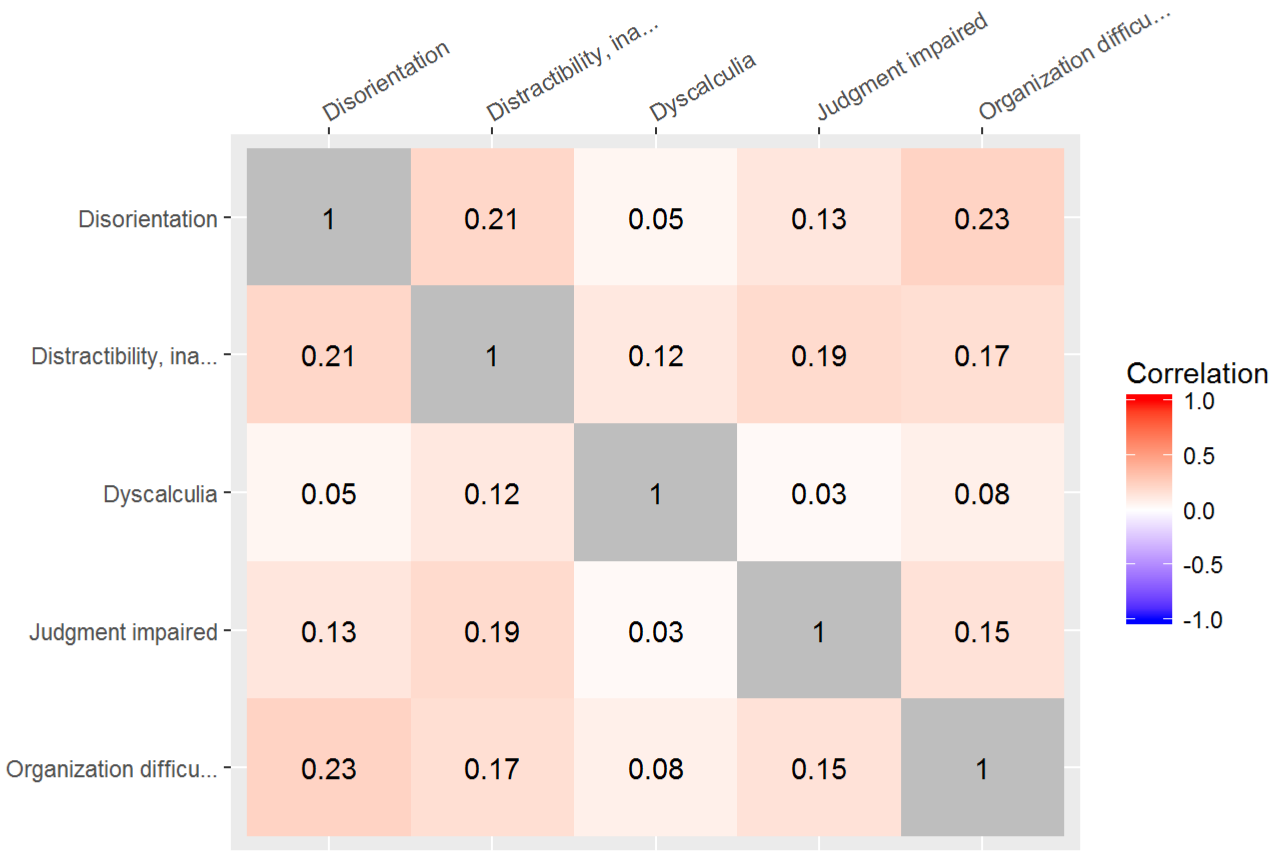

Current Gait Disorder Symptoms

Value	Percent	Count	Male	Female
None	53.6	1048	426	622
Decreased balance	21.1	412	179	233
Uses walker	11.9	232	73	159
Shuffling	11.3	222	119	103
Other (specify)	10.5	206	101	105
Uses cane	8.6	169	74	95
Uses wheelchair	4.5	88	28	60

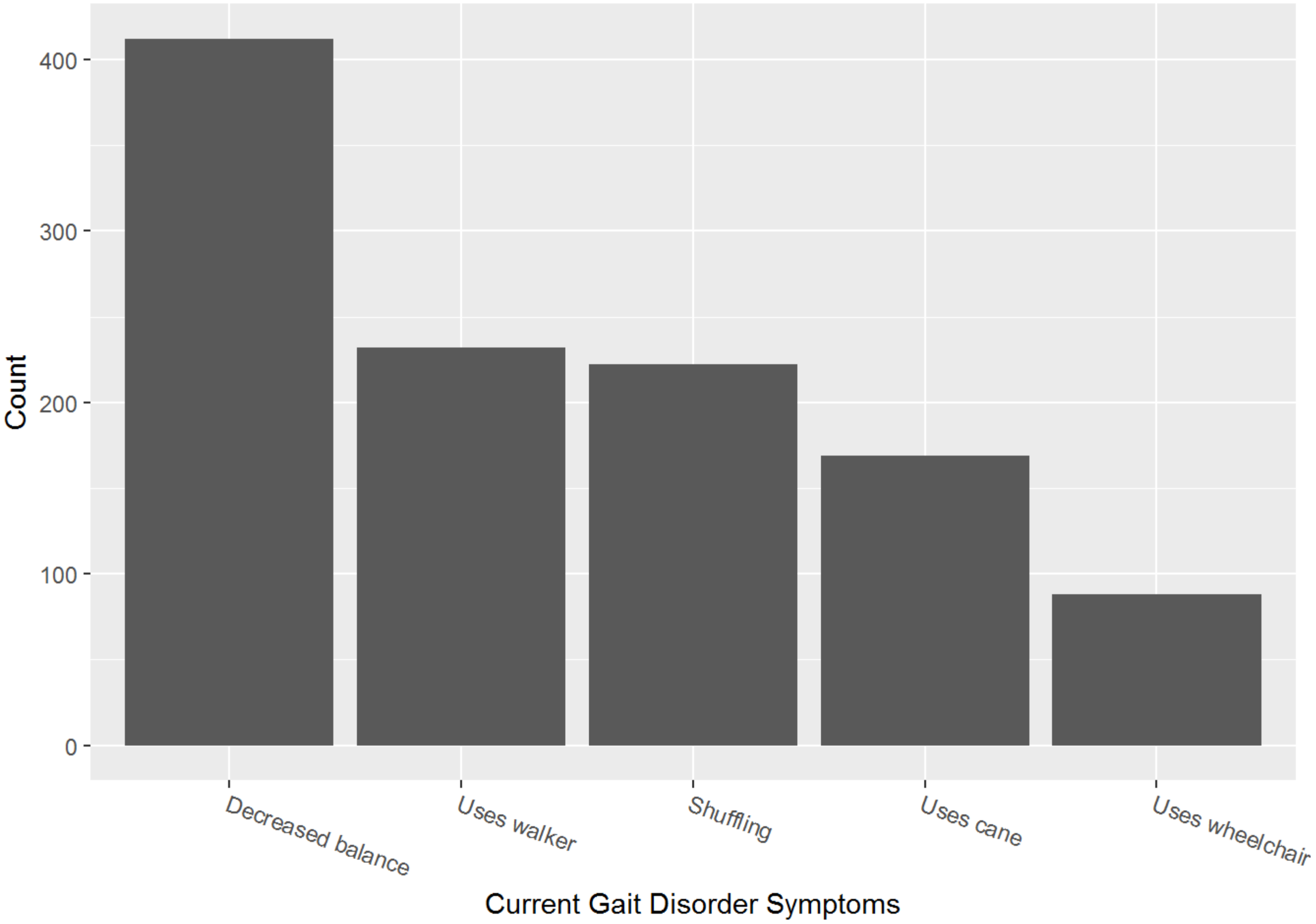

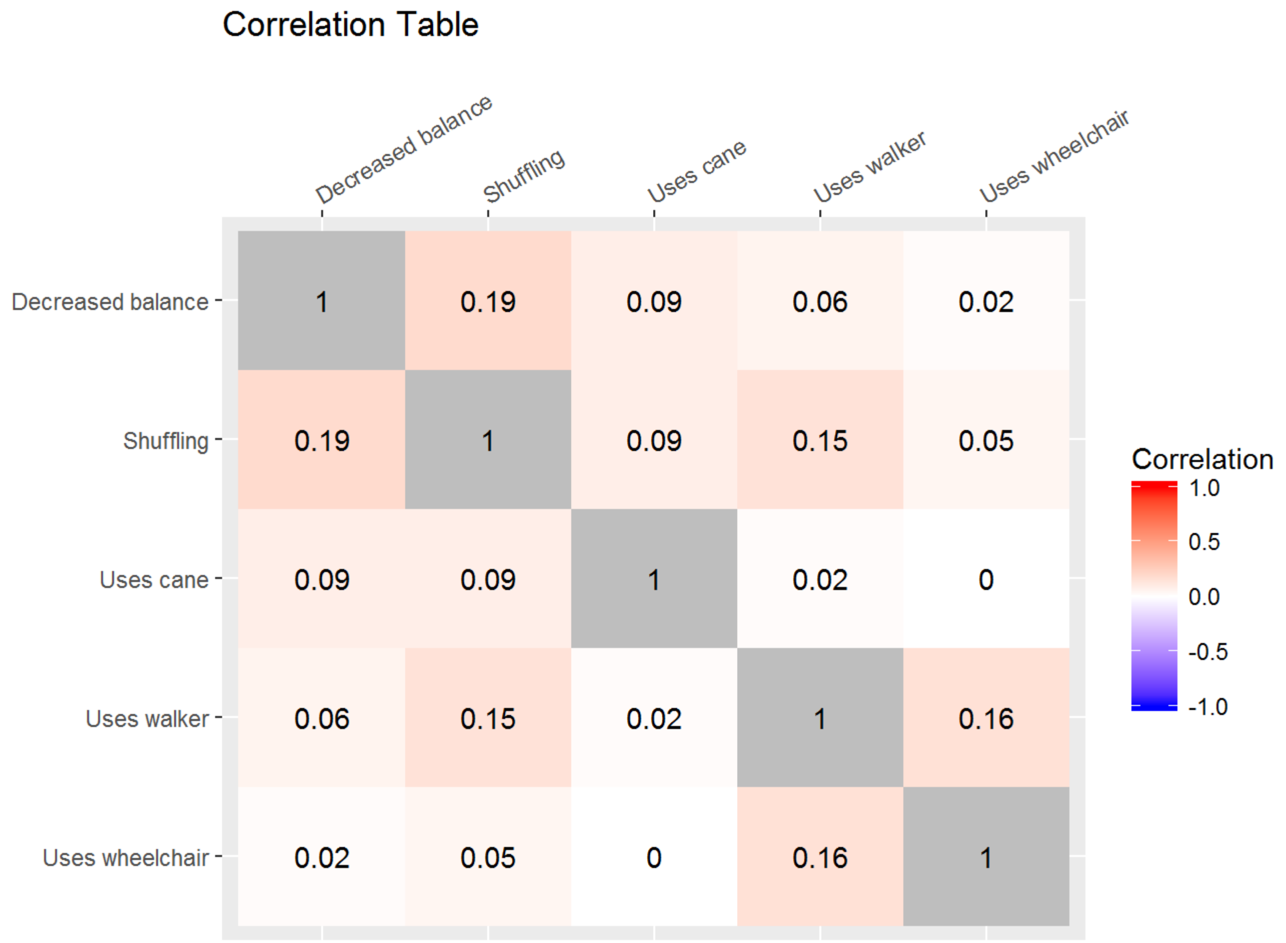

Current Fall Symptoms

Value	Percent	Count	Male	Female
None	65.3	1277	555	722
Mechanical	33	645	247	398
Associated injuries	5.9	116	27	89
Orthostatic lightheadedness	1.5	30	17	13
Other (specify)	0.7	13	4	9

Memory

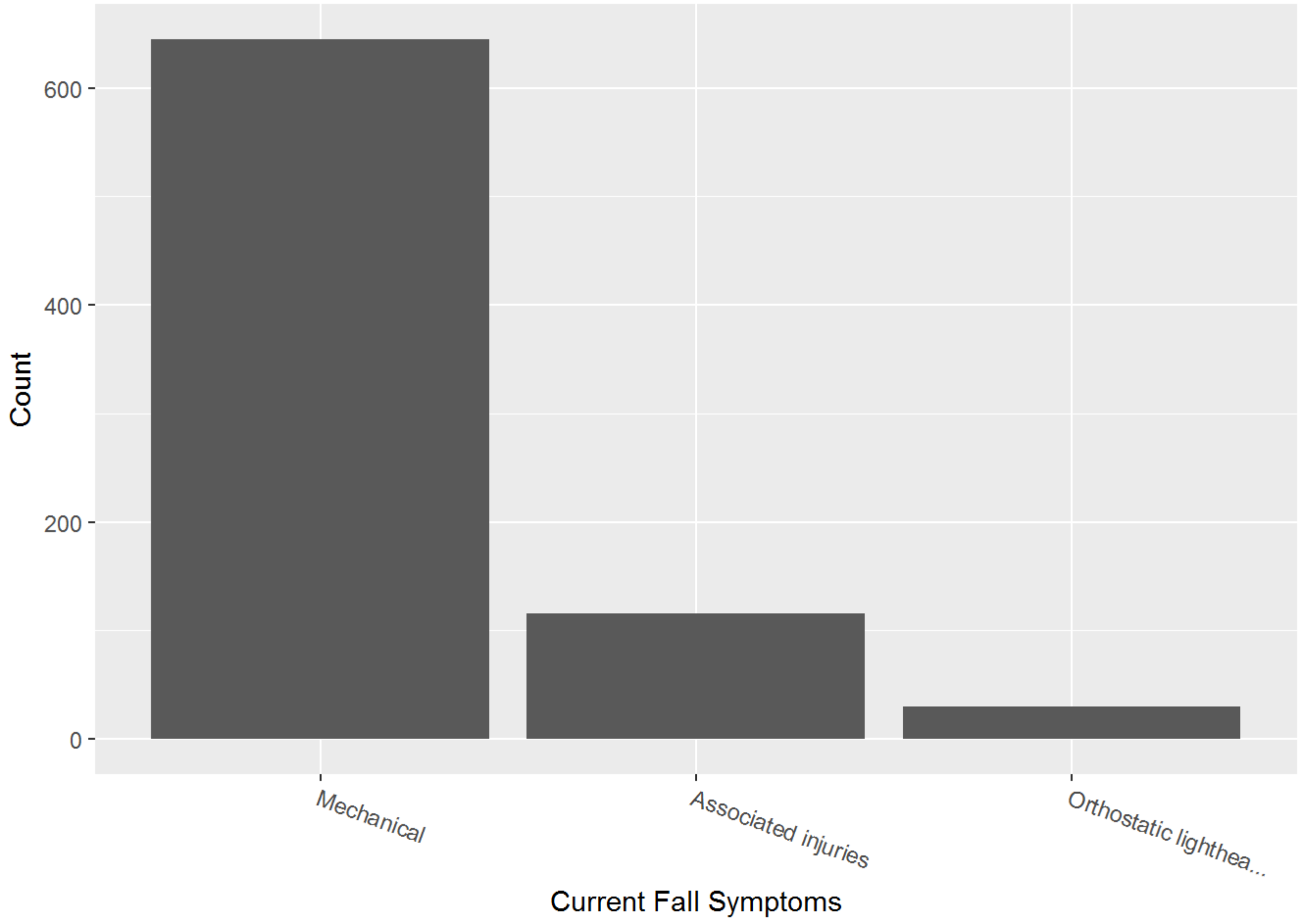

600
400
200
0
Count
Mechanical
Associated injuries
Orthostatic lighthea...
Current Fall Symptoms

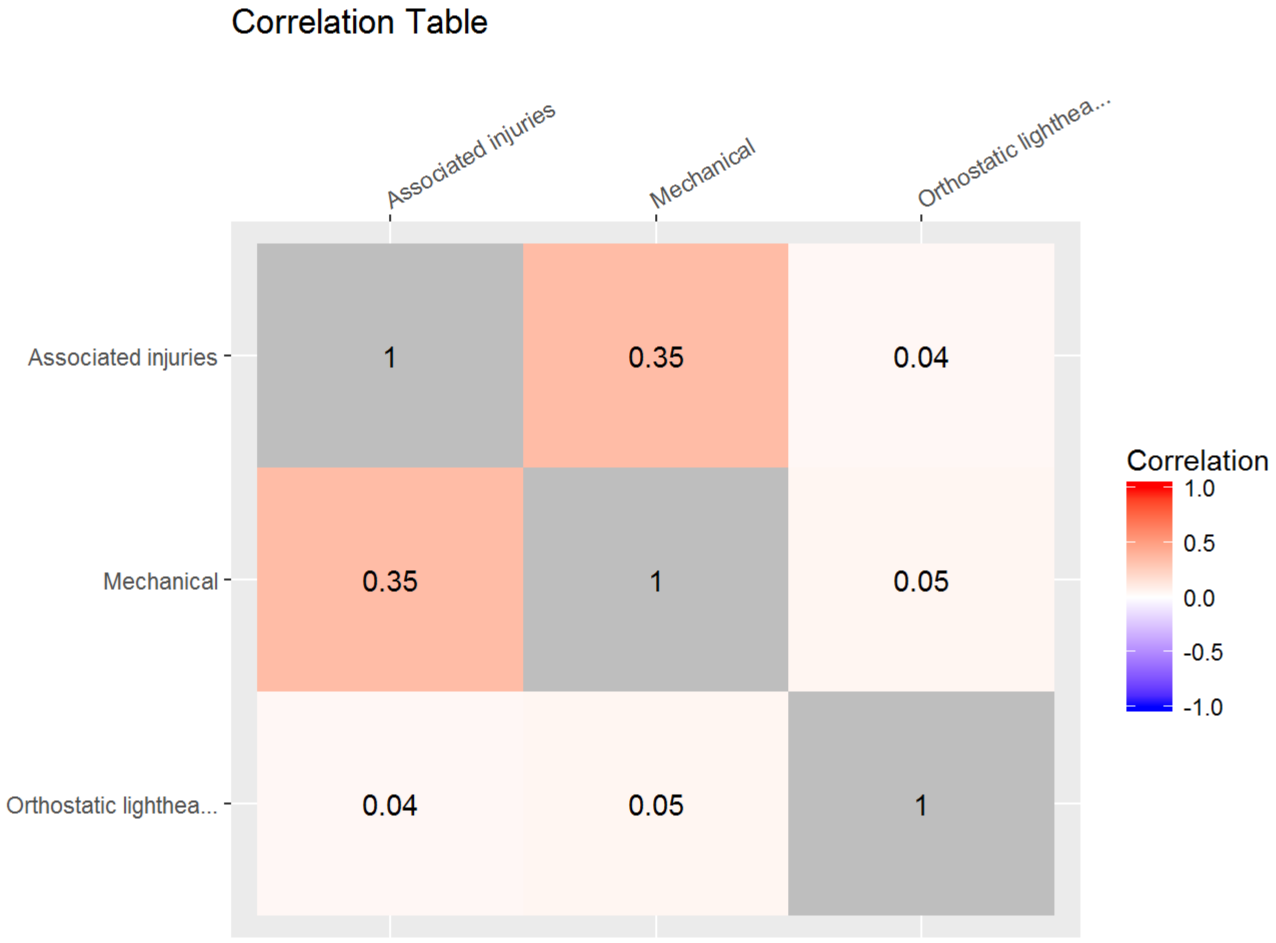

Correlation Table
Associated injuries
Mechanical
Orthostatic lighthea...
1
0.35
0.04
0.35
1
0.05
0.04
0.05
1
Correlation
1.0
0.5
0.0
-0.5
-1.0

Current Involuntary Movements Symptoms

Value	Percent	Count	Male	Female
None	84.9	1660	680	980
Tremor	12.8	251	111	140
Myoclonus	1.9	37	20	17
Other (specify)	1	20	12	8
Chorea	0.1	1	1	0

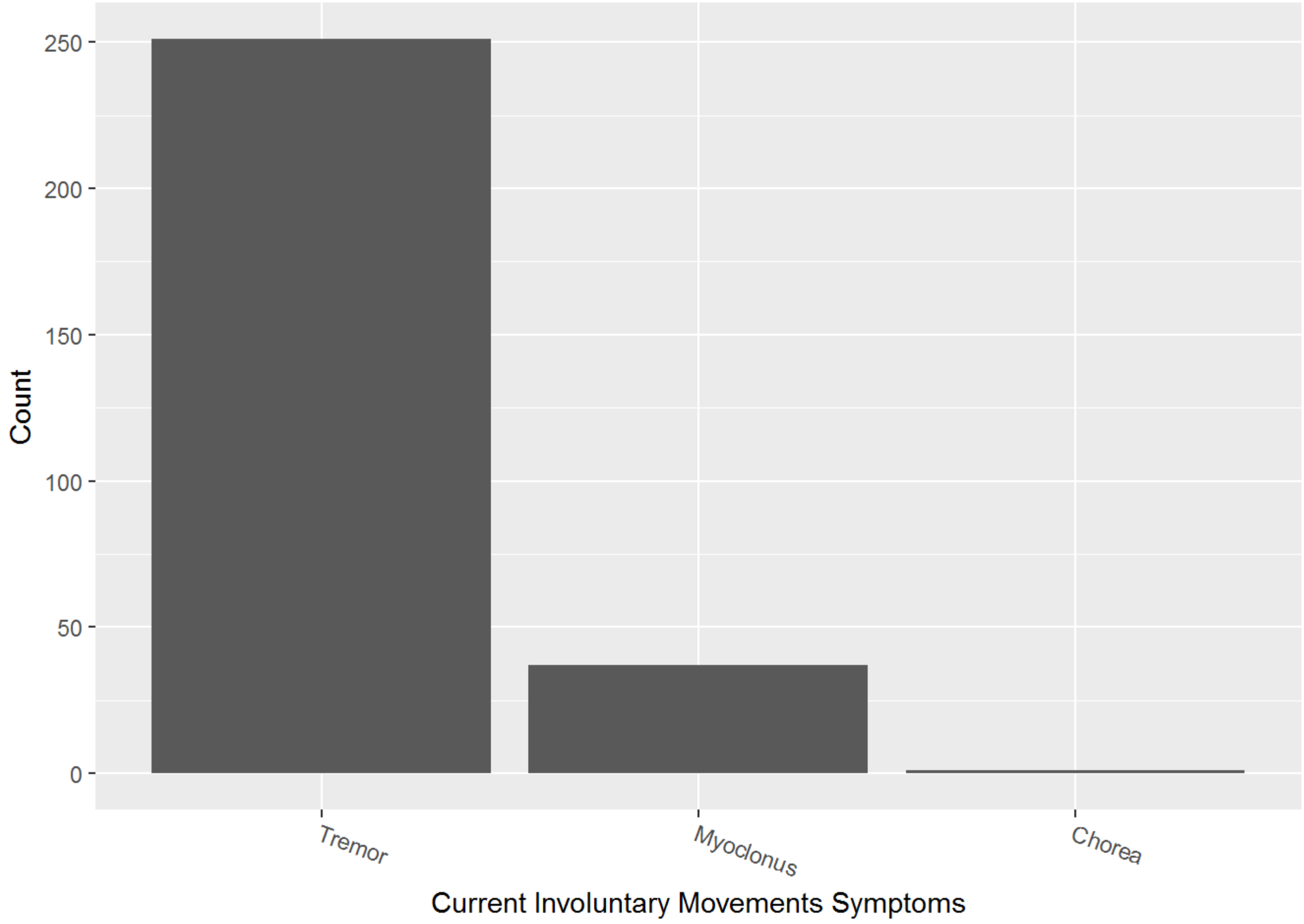

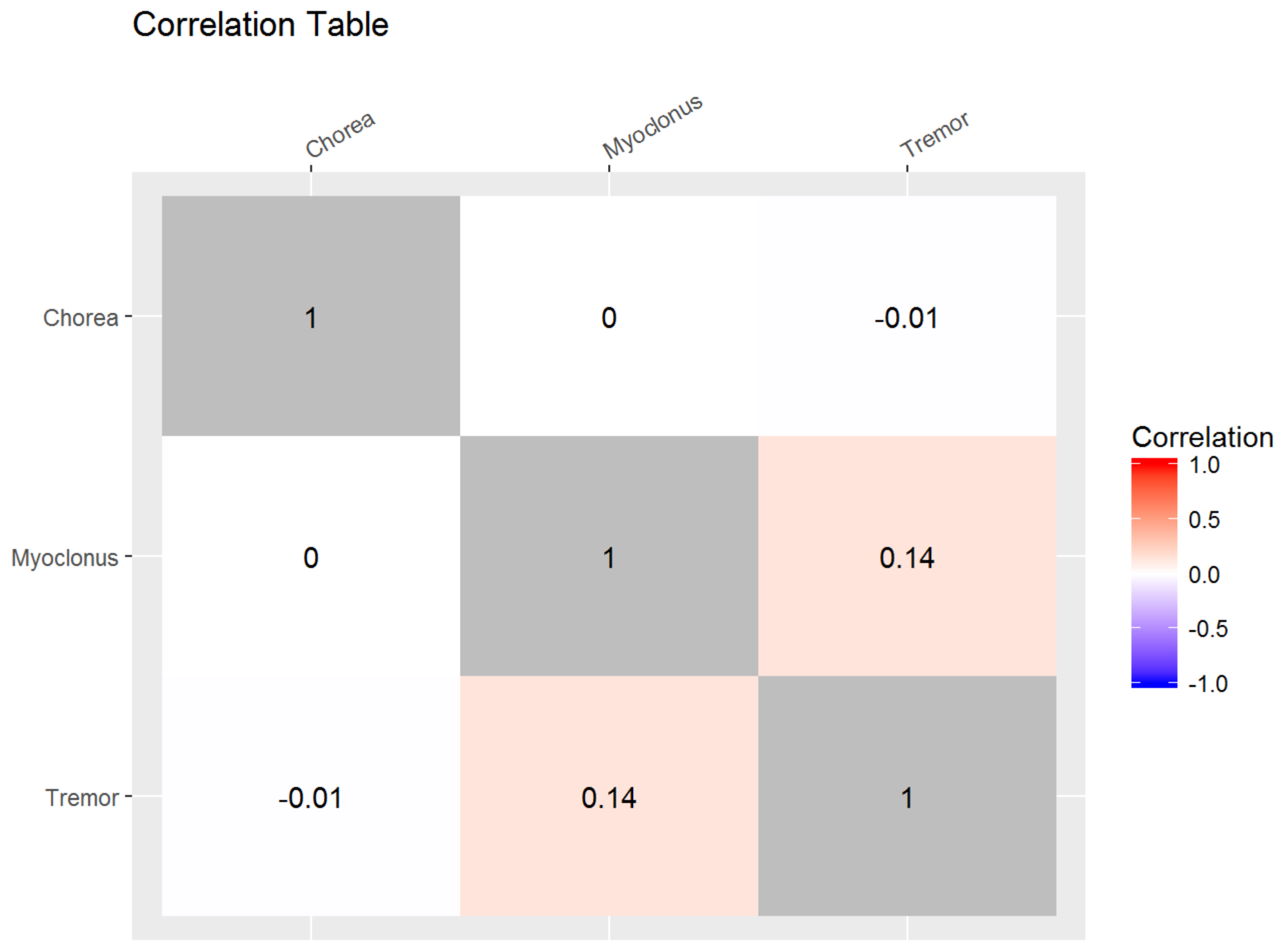

Current Apraxia Symptoms

Value	Percent	Count	Male	Female
None	96.2	1881	785	1096
No predominance	2.7	53	24	29
Right side predominant	0.5	9	3	6
NA	0.4	7	2	5
Left side predominant	0.3	6	2	4

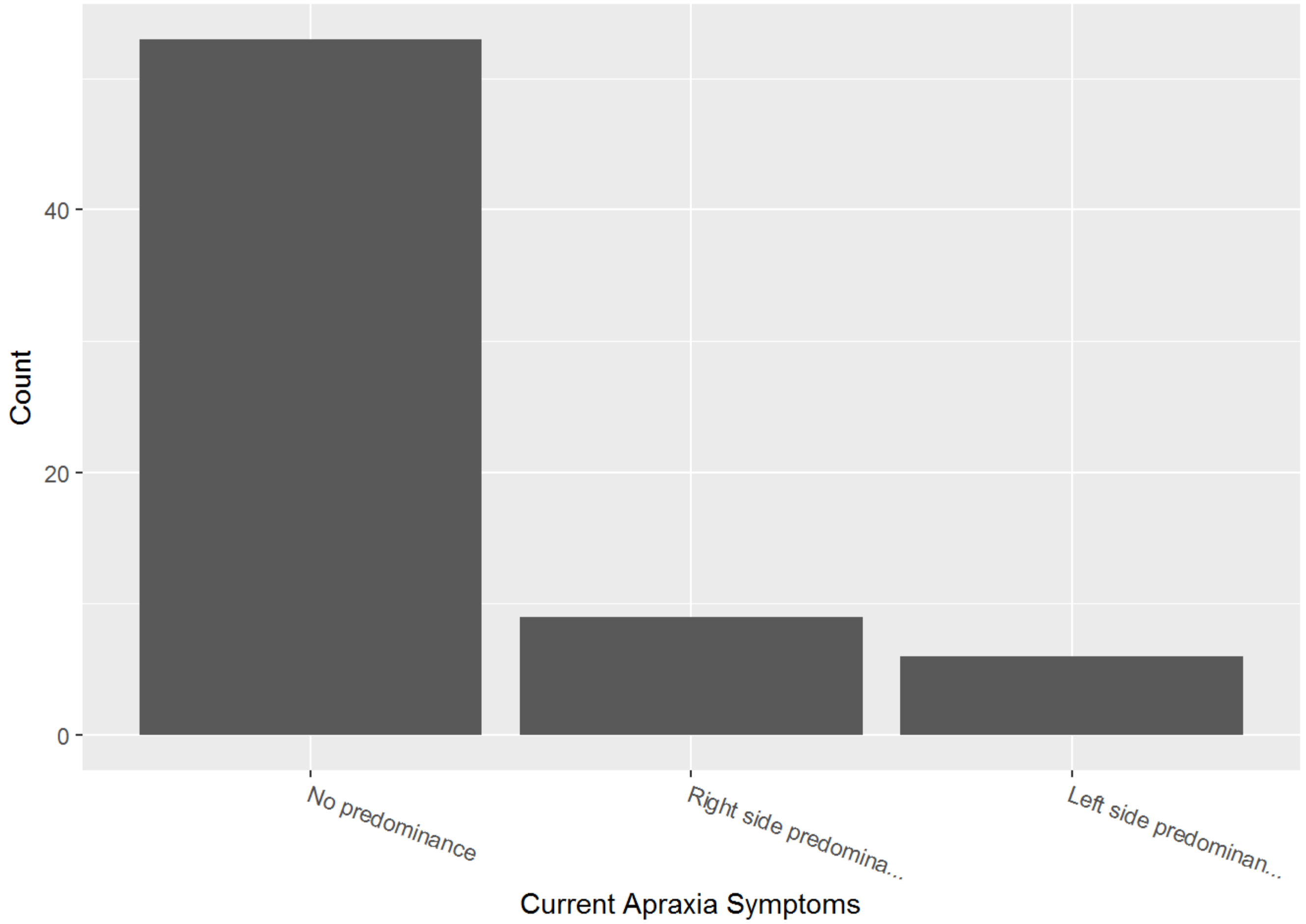

Current Behavioral Symptoms

Value	Percent	Count	Male	Female
Depression/dysphoria	34.5	674	235	439
Anxiety	33.5	656	233	423
Sleep disturbance	31.1	608	283	325
Irritability/lability	31	607	290	317
None	23.5	460	192	268
Delusions (persecution, misidentification, reduplication)	17.4	341	106	235
Apathy/indifference	16.5	323	150	173
Agitation/aggression	10.7	209	85	124
Hallucinations (visual, auditory)	8.8	173	71	102
Psychomotor disturbance (perseveration, obsessive-compulsive)	6.5	127	54	73
Appetite	3	59	21	38
Disinhibition	2.5	48	25	23
Hoarding	1.9	38	9	29
Other (specify)	0.9	17	12	5
Elation/euphoria	0.5	9	4	5

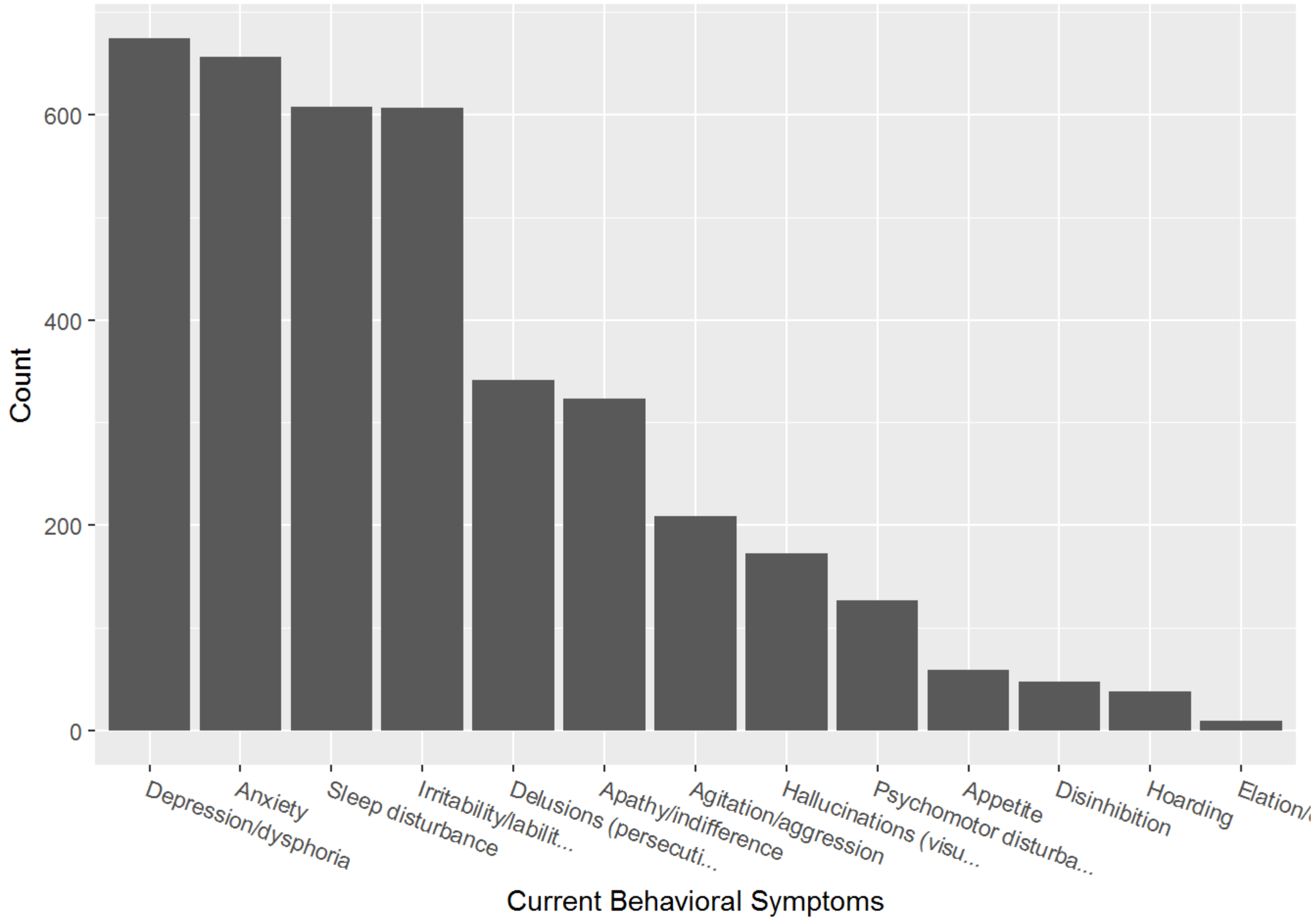
Count
600
400
200
0
Depression/dysphoria
Anxiety
Sleep disturbance
Irritability/labilit...
Delusions (persecuti...
Apathy/indifference
Agitation/aggression
Hallucinations (visu...
Psychomotor disturba...
Appetite
Disinhibition
Hoarding
Elation/
Current Behavioral Symptoms

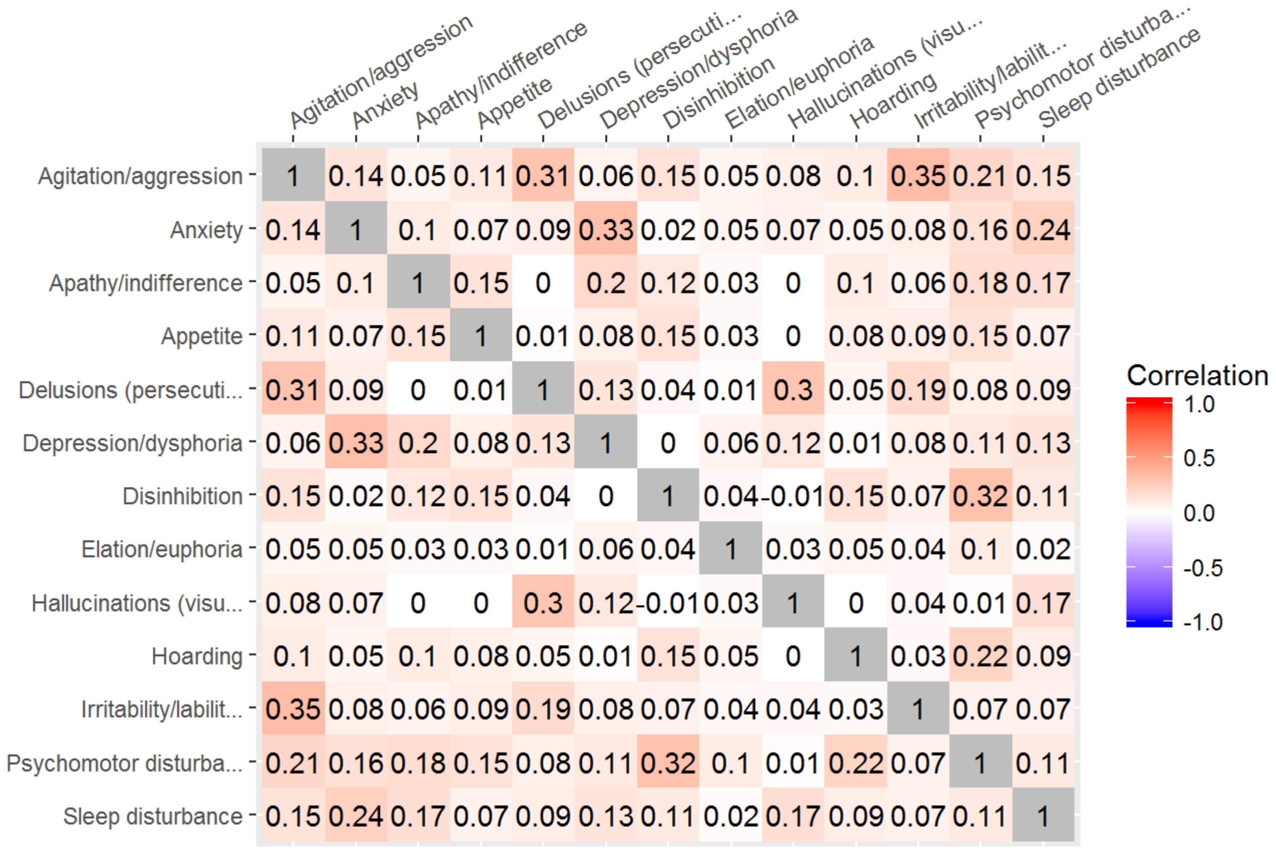
Correlation Table
Agitation/aggression Anxiety Apathy/indifference Appetite Delusions (persecuti... Depression/dysphoria Disinhibition Elation/euphoria Hallucinations (visu... Hoarding Irritability/labilit... Psychomotor disturba... Sleep disturbance
Agitation/aggression 1 0.14 0.05 0.11 0.31 0.06 0.15 0.05 0.08 0.1 0.35 0.21 0.15
Anxiety 0.14 1 0.1 0.07 0.09 0.33 0.02 0.05 0.07 0.05 0.08 0.16 0.24
Apathy/indifference 0.05 0.1 1 0.15 0 0.2 0.12 0.03 0 0.1 0.06 0.18 0.17
Appetite 0.11 0.07 0.15 1 0.01 0.08 0.15 0.03 0 0.08 0.09 0.15 0.07
Delusions (persecuti... 0.31 0.09 0 0.01 1 0.13 0.04 0.01 0.3 0.05 0.19 0.08 0.09
Depression/dysphoria 0.06 0.33 0.2 0.08 0.13 1 0 0.06 0.12 0.01 0.08 0.11 0.13
Disinhibition 0.15 0.02 0.12 0.15 0.04 0 1 0.04 -0.01 0.15 0.07 0.32 0.11
Elation/euphoria 0.05 0.05 0.03 0.03 0.01 0.06 0.04 1 0.03 0.05 0.04 0.1 0.02
Hallucinations (visu... 0.08 0.07 0 0 0.3 0.12 -0.01 0.03 1 0 0.04 0.01 0.17
Hoarding 0.1 0.05 0.1 0.08 0.05 0.01 0.15 0.05 0 1 0.03 0.22 0.09
Irritability/labilit... 0.35 0.08 0.06 0.09 0.19 0.08 0.07 0.04 0.04 0.03 1 0.07 0.07
Psychomotor disturba... 0.21 0.16 0.18 0.15 0.08 0.11 0.32 0.1 0.01 0.22 0.07 1 0.11
Sleep disturbance 0.15 0.24 0.17 0.07 0.09 0.13 0.11 0.02 0.17 0.09 0.07 0.11 1
Correlation
1.0
0.5
0.0
-0.5
-1.0

Current Additional Symptoms

Value	Percent	Count	Male	Female
None	87.4	1710	695	1015
Other (specify)	5.3	103	58	45
Anosmia	3.4	66	31	35
Wandering	2	39	16	23
Seizures	1.8	36	19	17
Autonomic	0.7	14	10	4

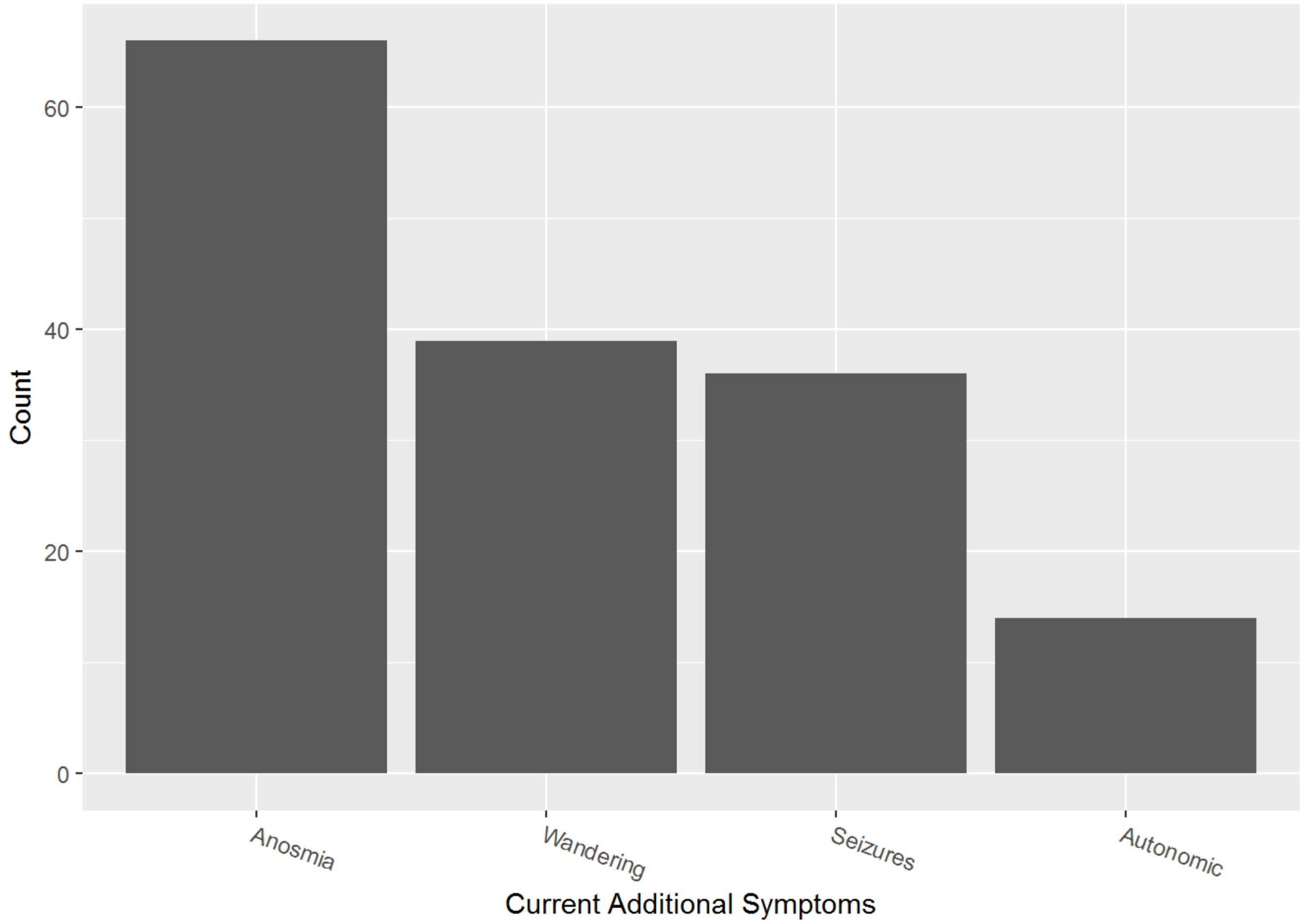

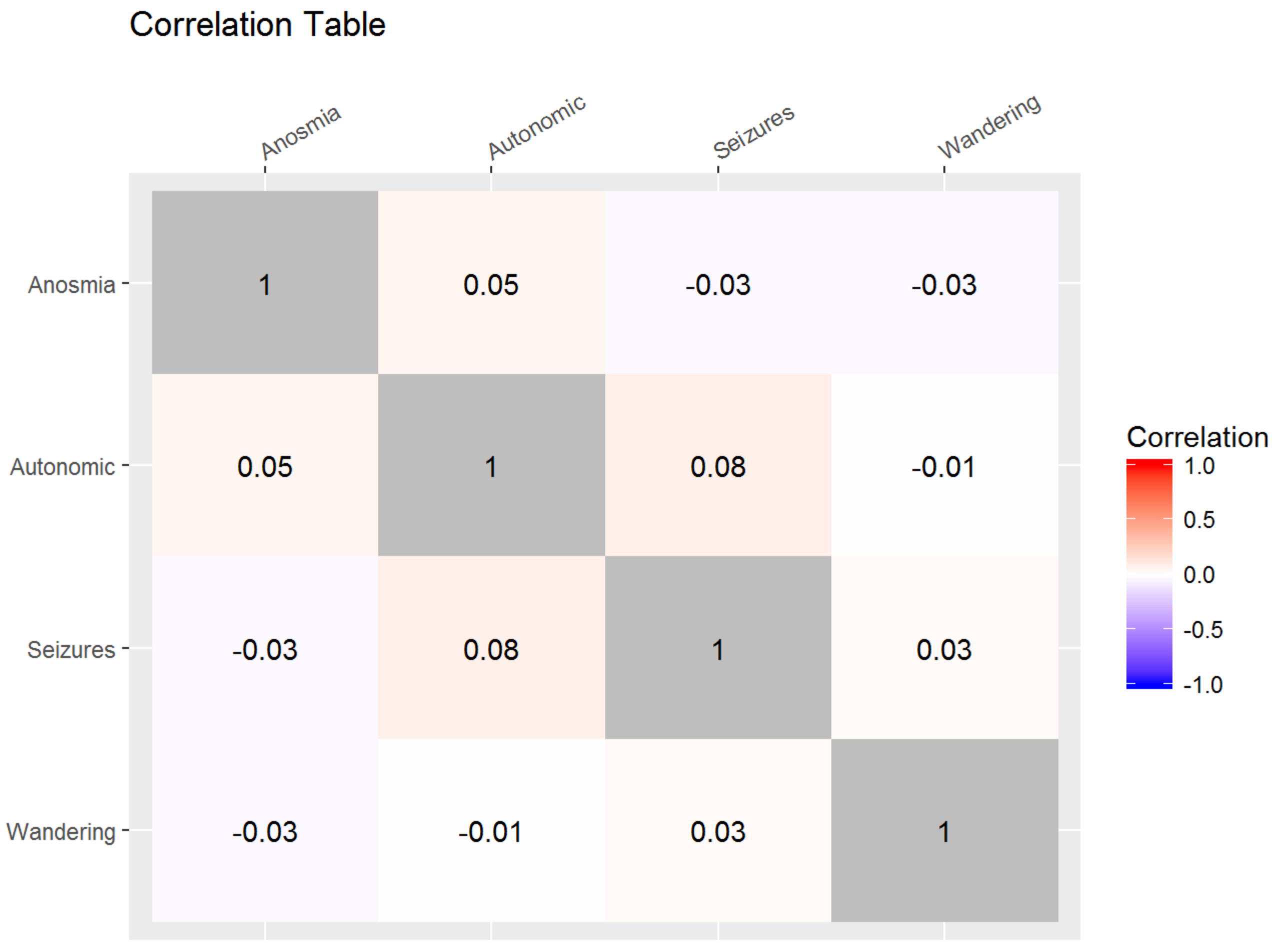

Current Rx

Value	Percent	Count	Male	Female
None	50.3	984	445	539
Antidepressants	29.3	573	197	376
Donepezil	17.8	348	144	204
Anxiolytics	9	176	62	114
Antipsychotics	6.5	127	42	85
Memantine	6.4	125	51	74
Memantine XR	2.4	46	16	30
Other	1.5	30	16	14
Rivastigmine Transdermal	1.1	22	9	13
Galantamine ER	0.7	14	6	8
Rivastigmine oral	0.5	9	6	3
Rivastigmine trasdermal	0.4	8	5	3
Galantamine	0.3	6	4	2

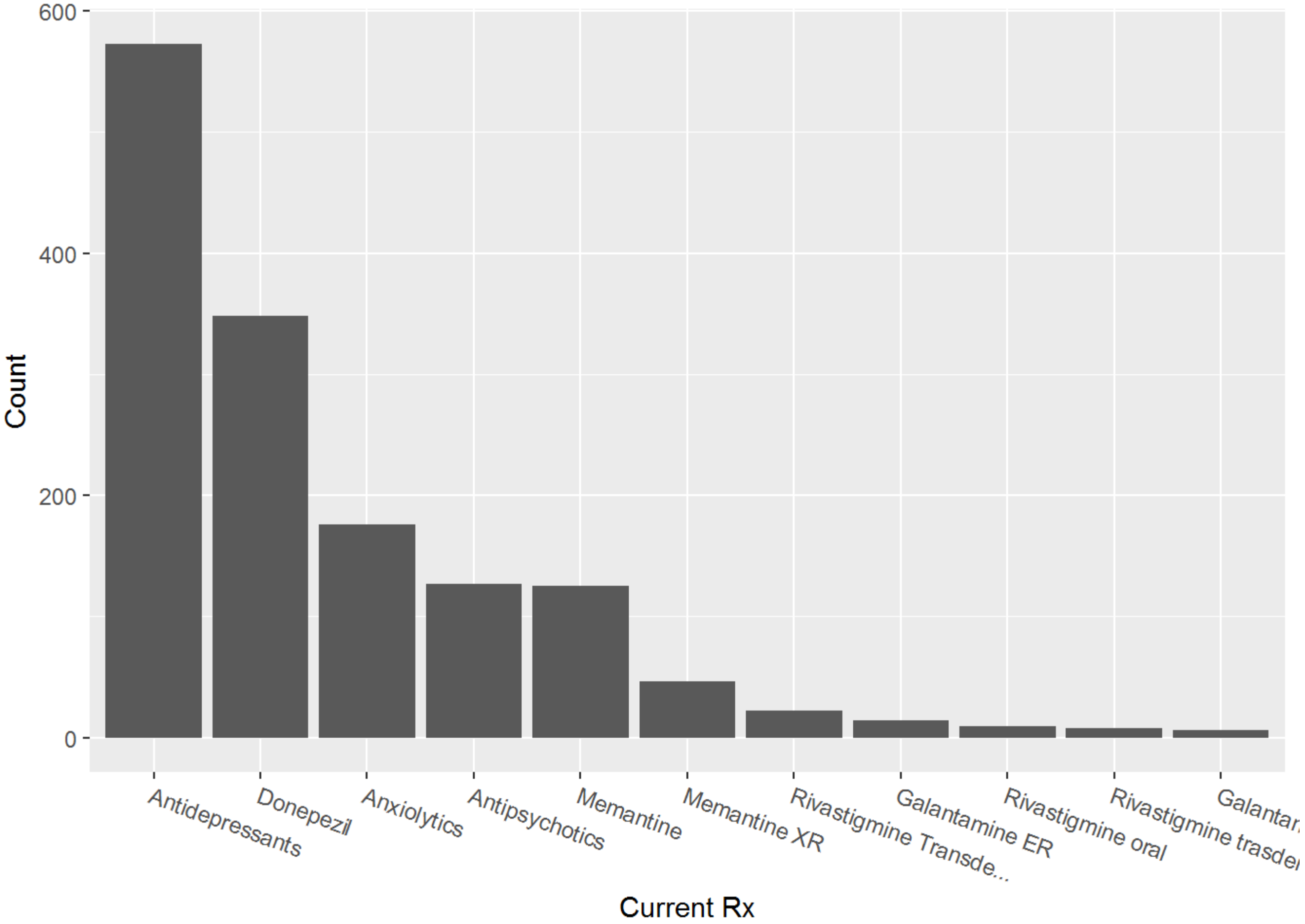

Correlation Table

	Antidepressants	Antipsychotics	Anxiolytics	Donepezil	Galantamine	Galantamine ER	Memantine	Memantine XR	Rivastigmine oral	Rivastigmine Transde...	Rivastigmine trasder ...
Antidepressants	1	0.15	0.21	0.05	0	0	0.02	0.03	-0.01	0.01	0.03
Antipsychotics	0.15	1	0.08	0.09	0.02	0.1	0.05	0.04	0.01	0.03	0.05
Anxiolytics	0.21	0.08	1	-0.05	-0.02	0.02	-0.01	0	-0.02	0.05	0.04
Donepezil	0.05	0.09	-0.05	1	0.05	-0.04	0.29	0.14	-0.03	-0.05	-0.03
Galantamine	0	0.02	-0.02	0.05	1	0	-0.01	0.05	0	-0.01	0
Galantamine ER	0	0.1	0.02	-0.04	0	1	0.1	0.03	-0.01	0.05	-0.01
Memantine	0.02	0.05	-0.01	0.29	-0.01	0.1	1	-0.04	0.04	0.03	0.08
Memantine XR	0.03	0.04	0	0.14	0.05	0.03	-0.04	1	0.04	0.05	0.1
Rivastigmine oral	-0.01	0.01	-0.02	-0.03	0	-0.01	0.04	0.04	1	-0.01	0
Rivastigmine Transde...	0.01	0.03	0.05	-0.05	-0.01	0.05	0.03	0.05	-0.01	1	-0.01
Rivastigmine trasder...	0.03	0.05	0.04	-0.03	0	-0.01	0.08	0.1	0	-0.01	1

Correlation: 1.0, 0.5, 0.0, -0.5, -1.0

Prior Rx

Value	Percent	Count	Male	Female
None	79	1545	657	888
Antidepressants	9.1	178	60	118
Donepezil	6.8	133	55	78
Benzodiazepines	3.1	61	16	45
Antipsychotics	2.5	49	16	33
Memantine	2.2	43	19	24
Other	1	20	11	9
Rivastigmine Transdermal	0.8	15	8	7
Memantine XR	0.5	9	6	3
Anxiolytics	0.4	8	2	6
Galantamine	0.4	7	1	6
Galantamine ER	0.3	6	2	4
Rivastigmine oral	0.3	6	2	4
Rivastigmine trasdermal	0.3	5	3	2
Axona	0.1	2	1	1

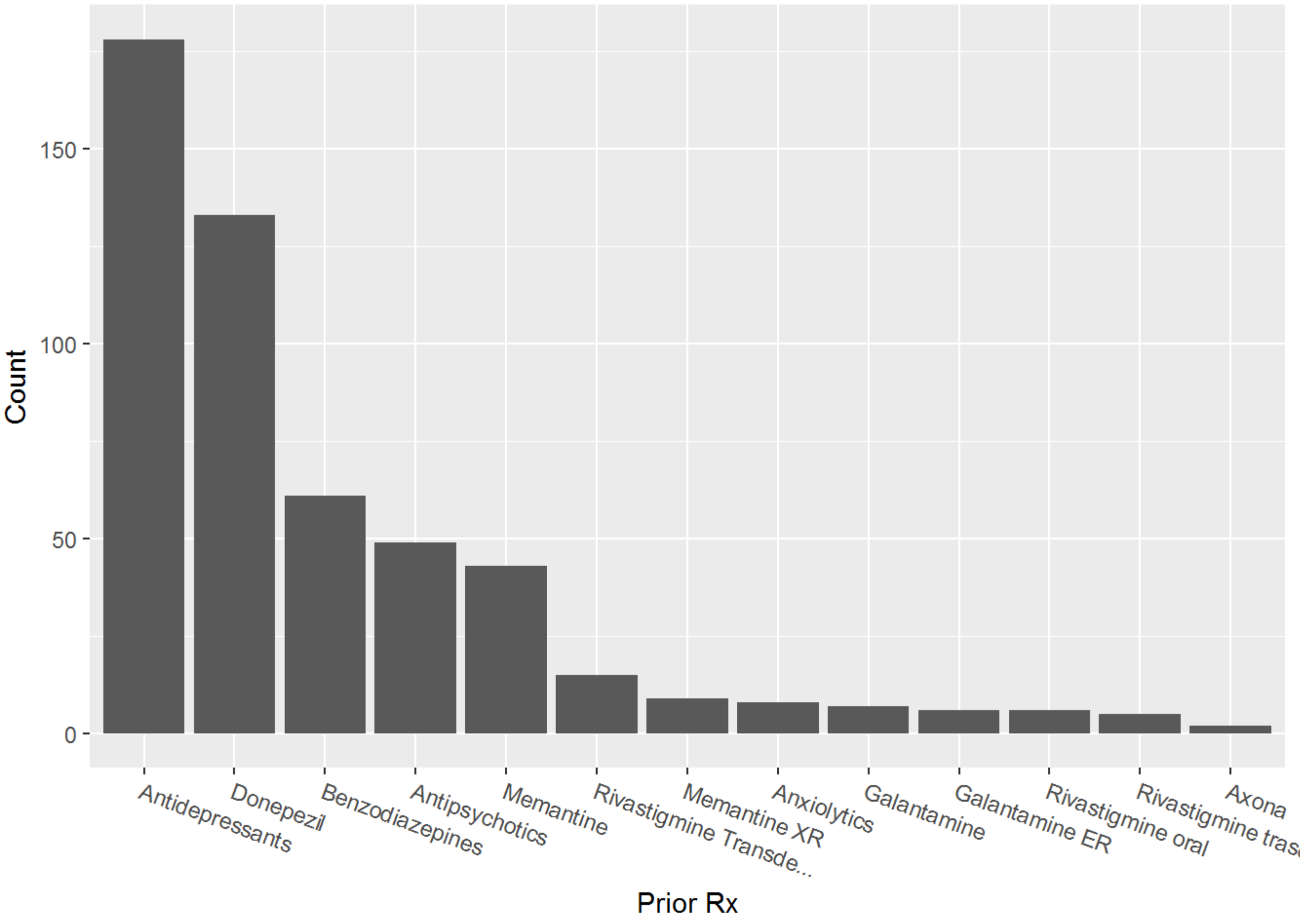

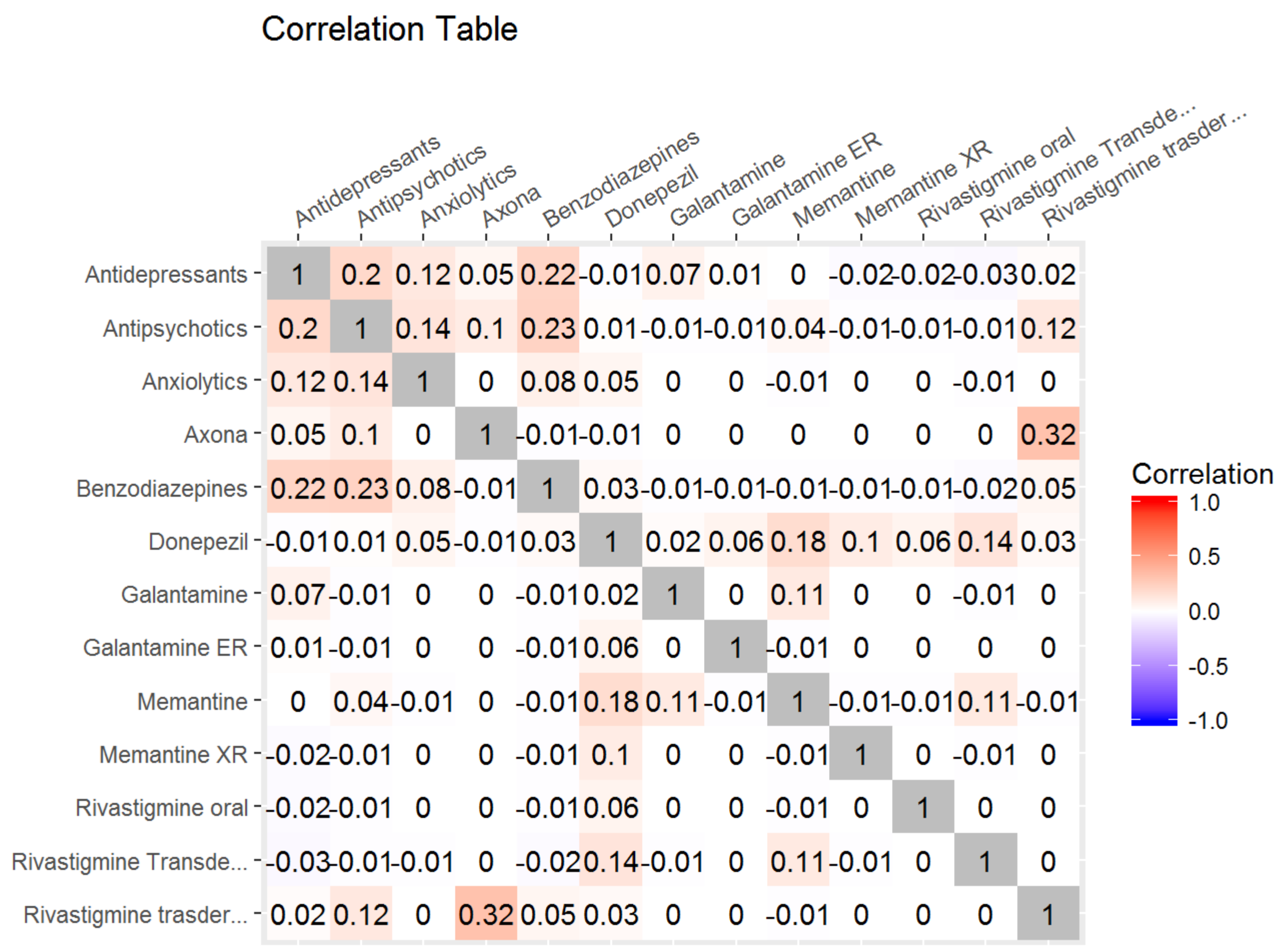

Live at Location

Value	Percent	Count	Male	Female
At home	89.6	1752	769	983
Assisted living/retirement home	8.9	174	40	134
Nursing home	1.2	23	7	16
NA	0.3	6	0	6
Religous community	0.1	1	0	1

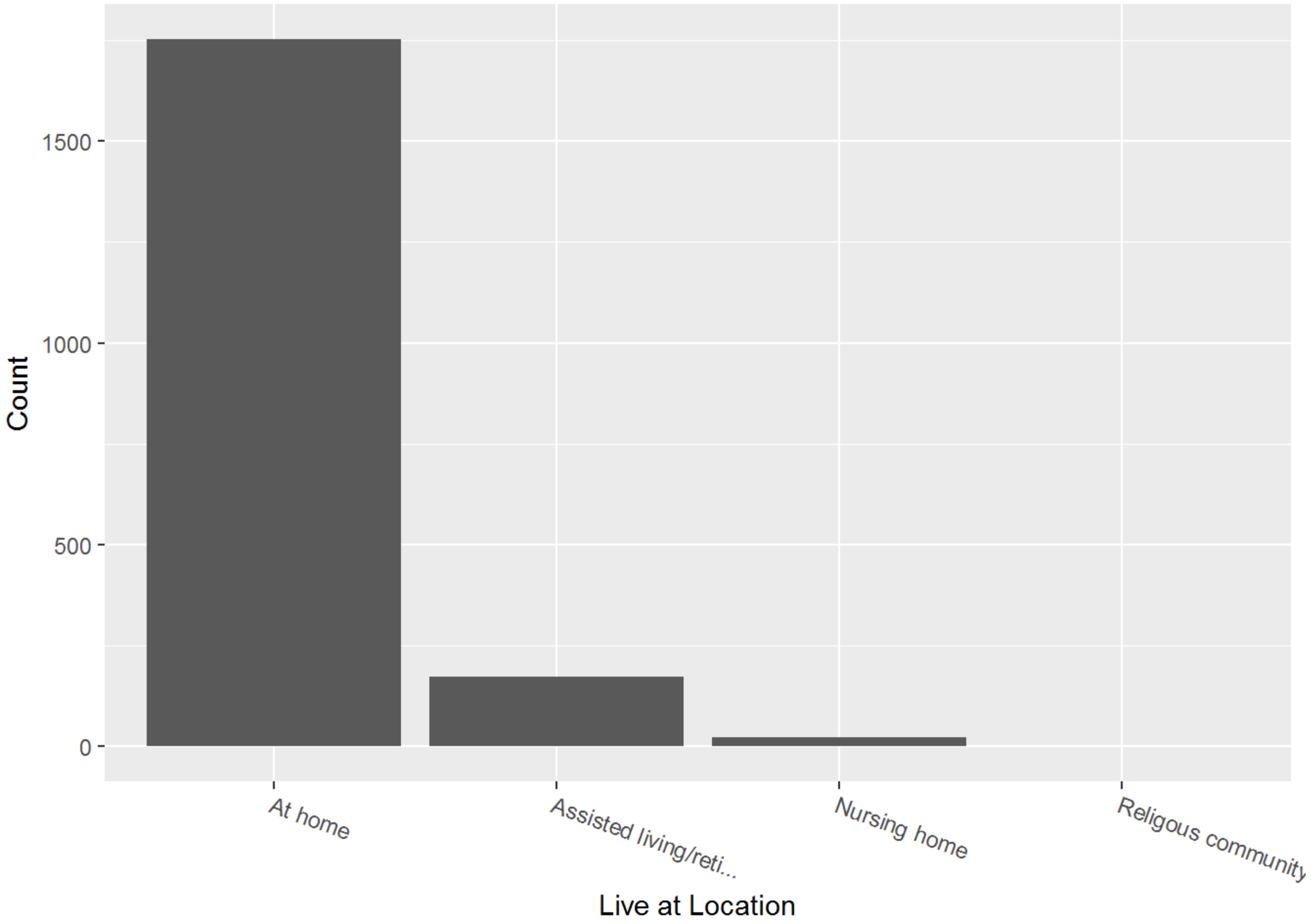

Caregiver

Value	Percent	Count	Male	Female
No	61.5	1202	481	721
Yes	38.3	749	333	416
NA	0.3	5	2	3

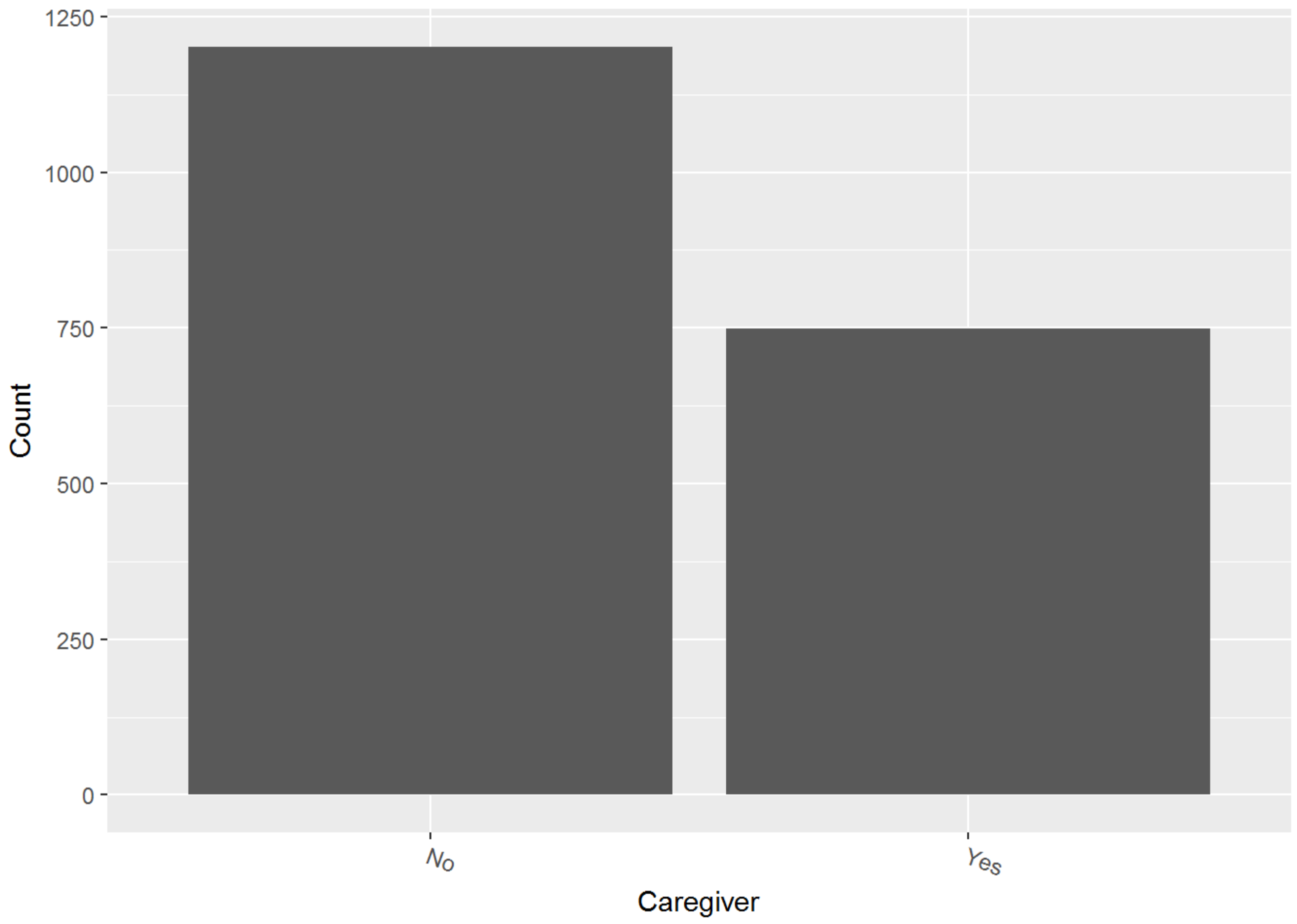

CT Head

Value	Percent	Count	Male	Female
No	53.3	1043	458	585
Yes	46.4	907	356	551
NA	0.3	6	2	4

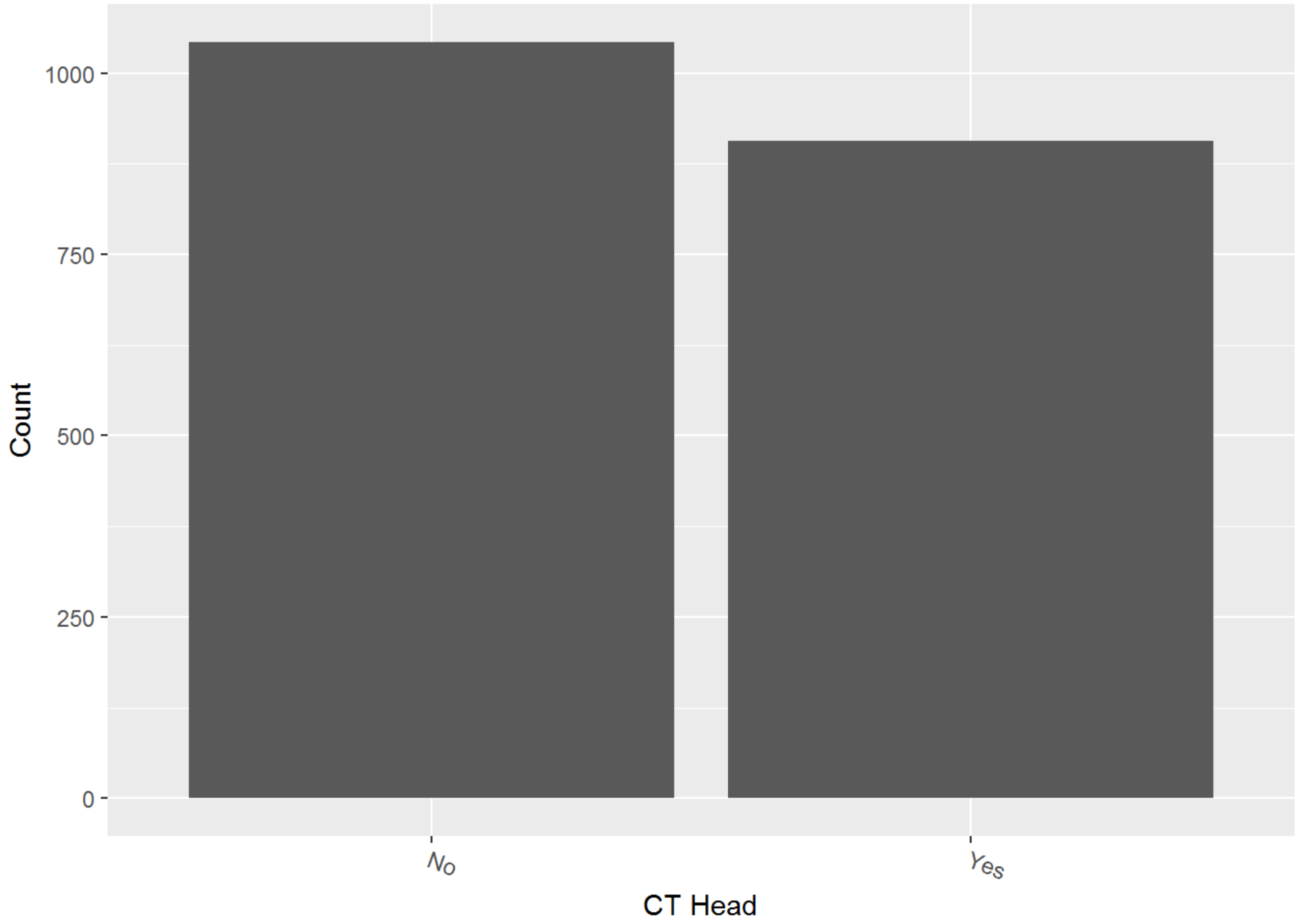

MRI Brain

Value	Percent	Count	Male	Female
Yes	50.2	981	420	561
No	49.5	968	393	575
NA	0.4	7	3	4

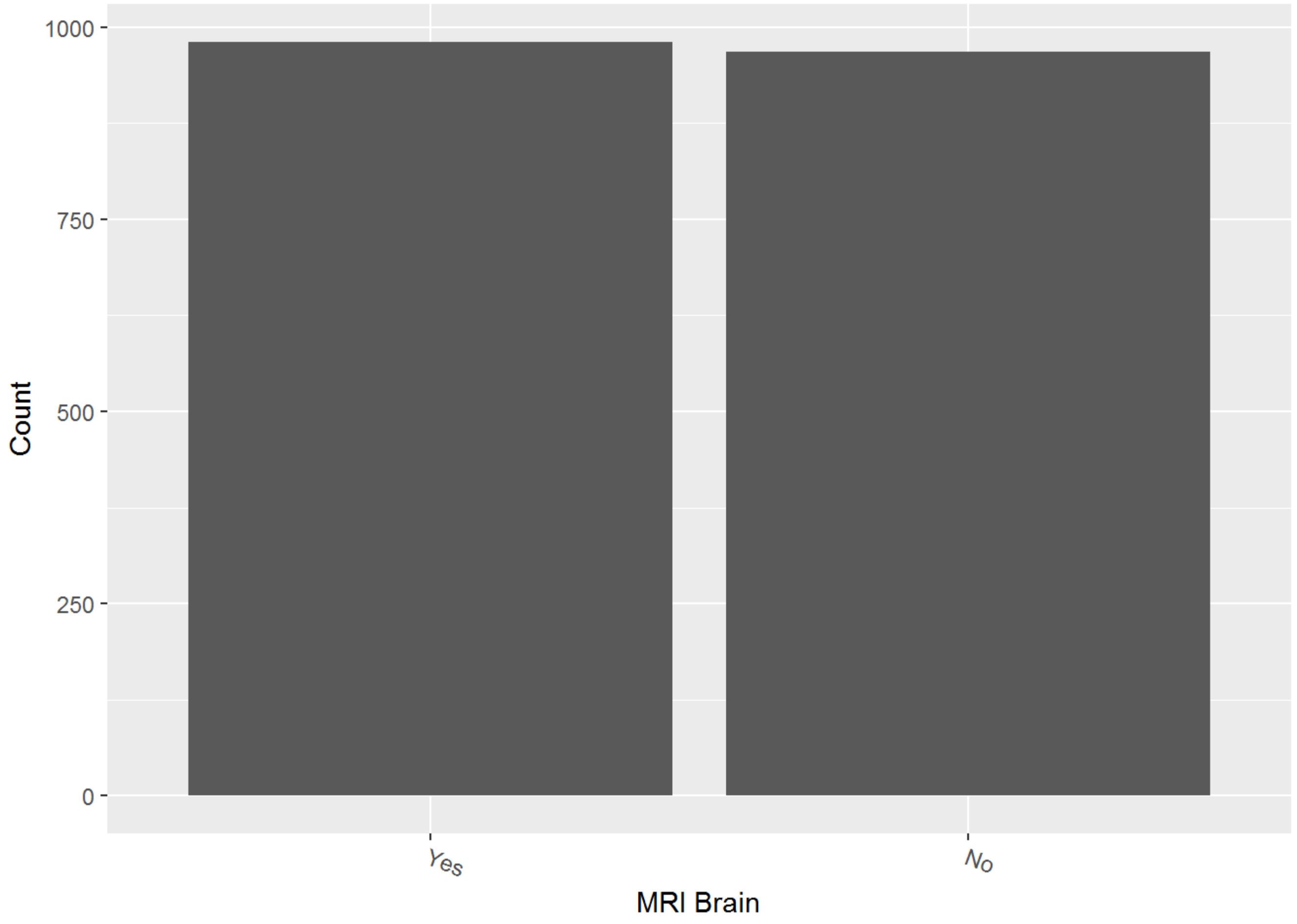

PET

Value	Percent	Count	Male	Female
No	98.7	1930	804	1126
Yes	1	20	10	10
NA	0.3	6	2	4

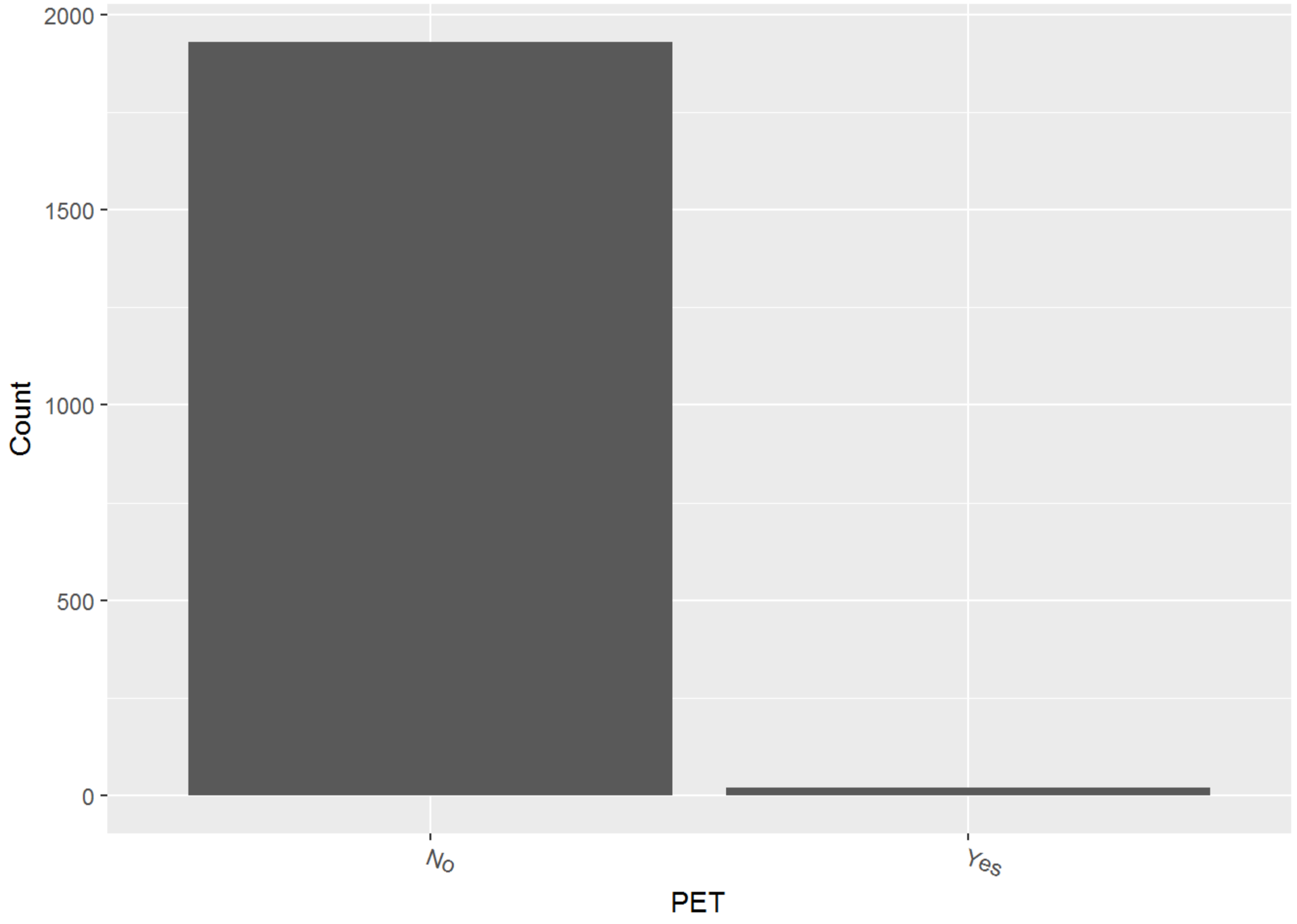

EEG

Value	Percent	Count	Male	Female
No	91.1	1782	727	1055
Yes	8.6	168	87	81
NA	0.3	6	2	4

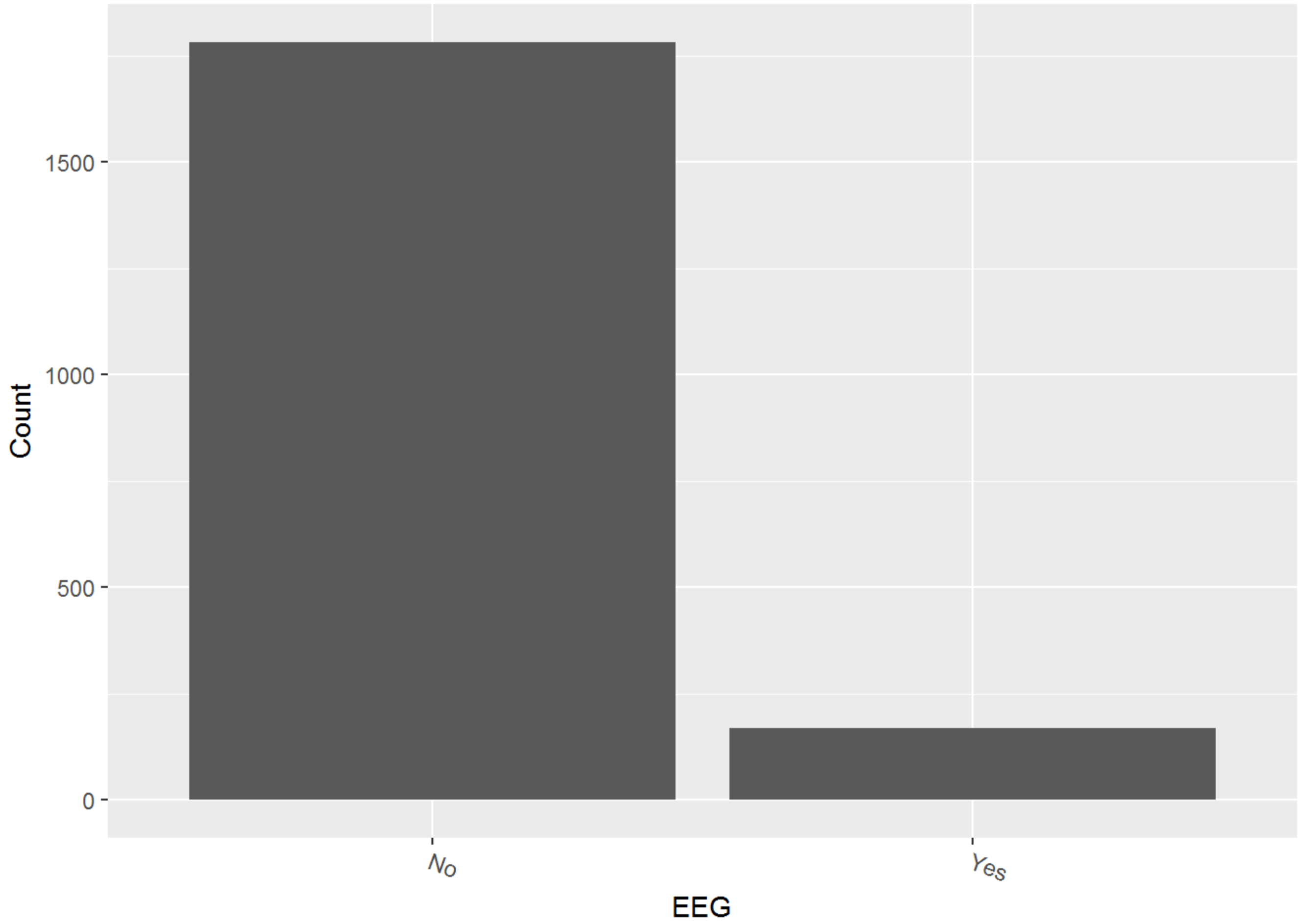

Neuropsych

Value	Percent	Count	Male	Female
No	73.6	1440	562	878
Yes	26.1	510	252	258
NA	0.3	6	2	4

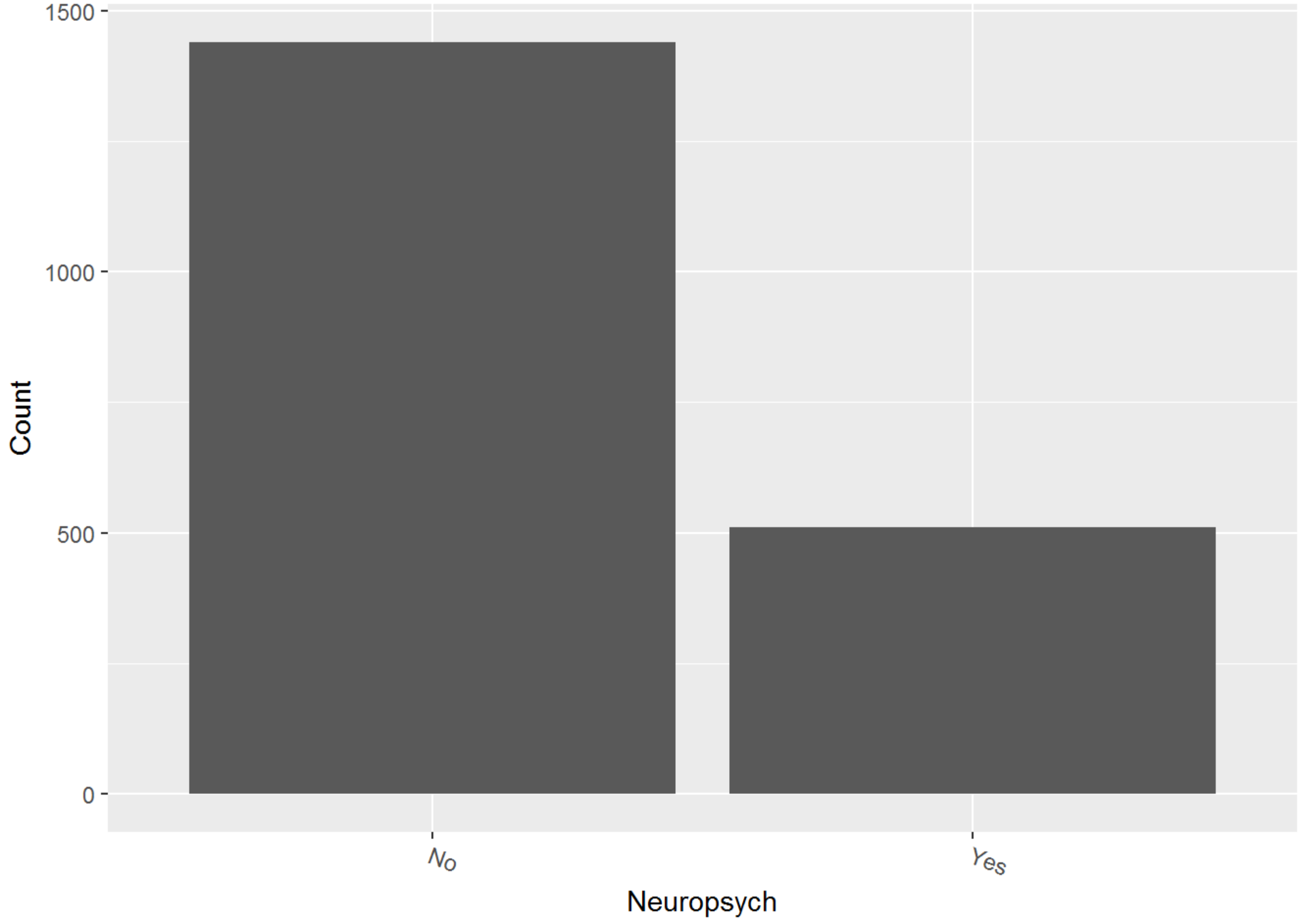

Cognitive Impairment Impression

Value	Percent	Count	Male	Female
Yes	99.6	1948	812	1136
NA	0.3	6	2	4
No	0.1	2	2	0

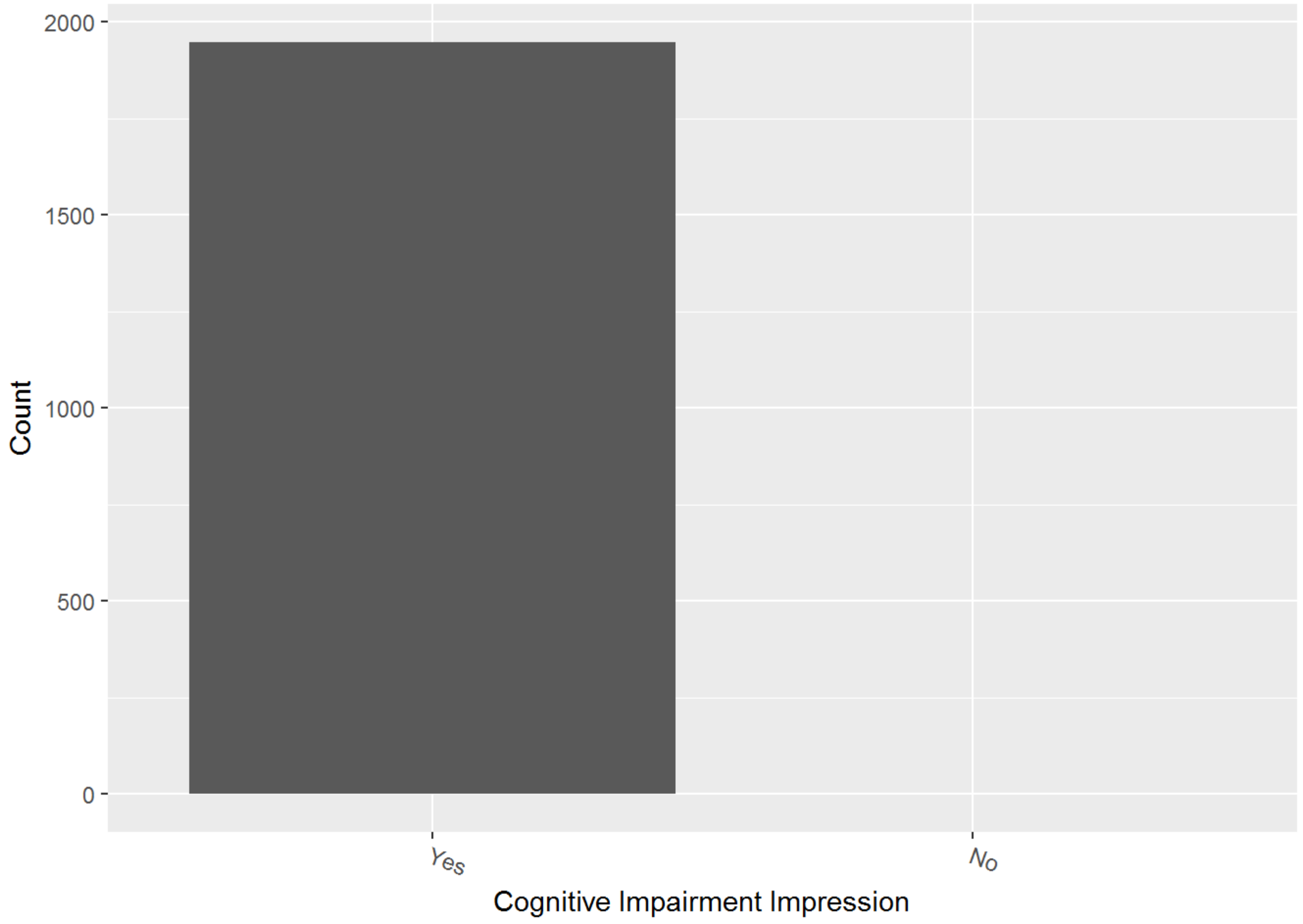

DSM-IV Criteria for Dementia Impression

Value	Percent	Count	Male	Female
Yes	59.8	1170	456	714
No	39.8	778	356	422
NA	0.4	8	4	4

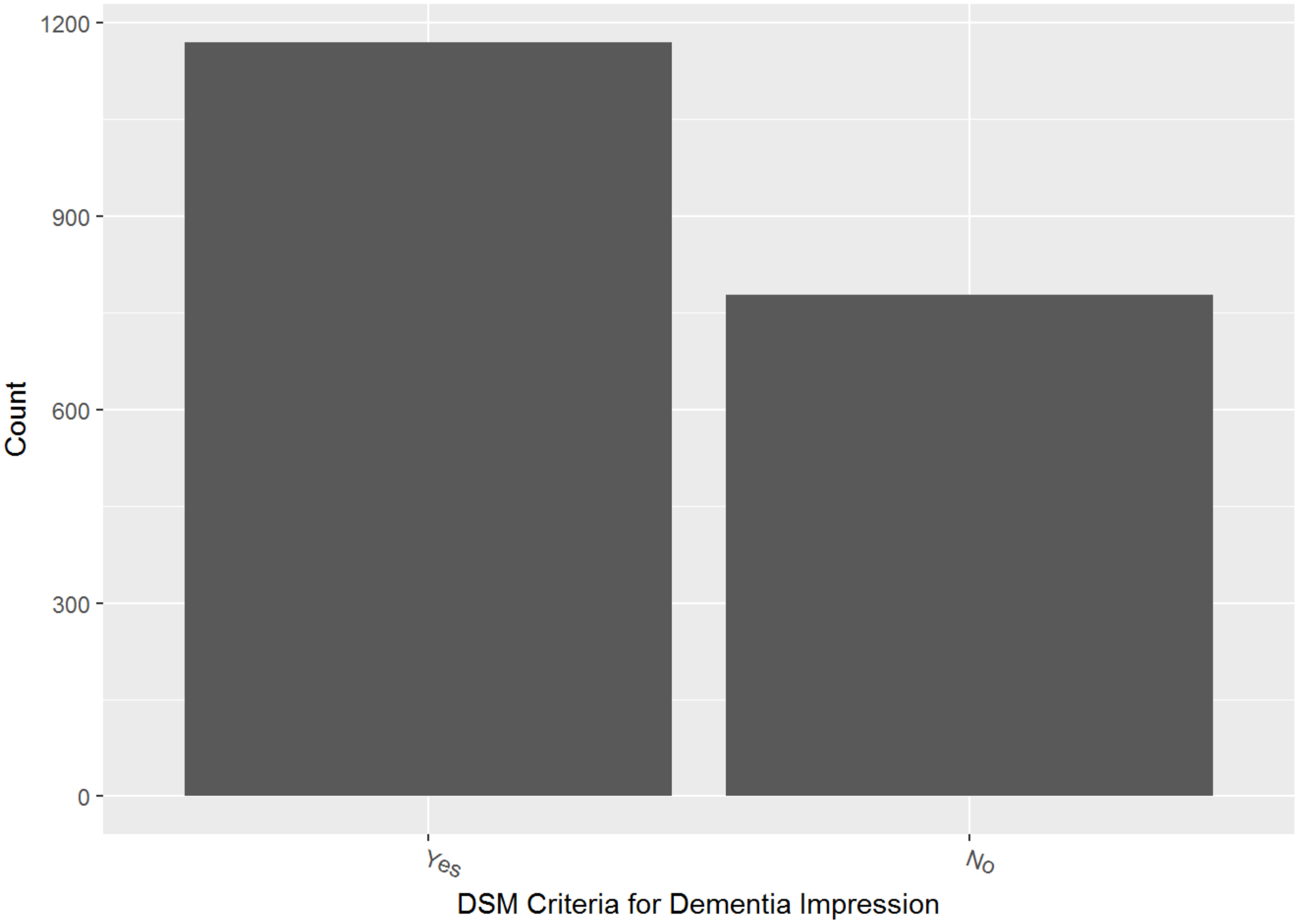

Dementia Cause

Value	Percent	Count	Male	Female
NA	40.5	793	364	429
Alzheimer’s disease	26	509	169	340
Not otherwise specified	17.3	339	145	194
Other (specify)	3.7	73	29	44
Vascular dementia	3.1	60	21	39
Alzheimer’s disease,Vascular dementia	2.6	51	22	29
Frontotemporal dementia	2.5	49	25	24
Lewy body dementia	1.7	33	22	11
Vascular dementia,Alzheimer’s disease	0.9	18	6	12
Alzheimer’s disease,Other (specify)	0.5	9	3	6
Vascular dementia,Other (specify)	0.5	9	5	4
Frontotemporal dementia,Other (specify)	0.2	3	0	3
Vascular dementia,Lewy body dementia	0.2	3	2	1
Alzheimer’s disease,Lewy body dementia	0.1	1	1	0
Alzheimer’s disease,Vascular dementia,Other (specify)	0.1	2	1	1
Frontotemporal dementia,Alzheimer’s disease	0.1	1	1	0
Not otherwise specified,Other (specify)	0.1	1	0	1
Other (specify),Alzheimer’s disease	0.1	1	0	1
Other (specify),Vascular dementia	0.1	1	0	1

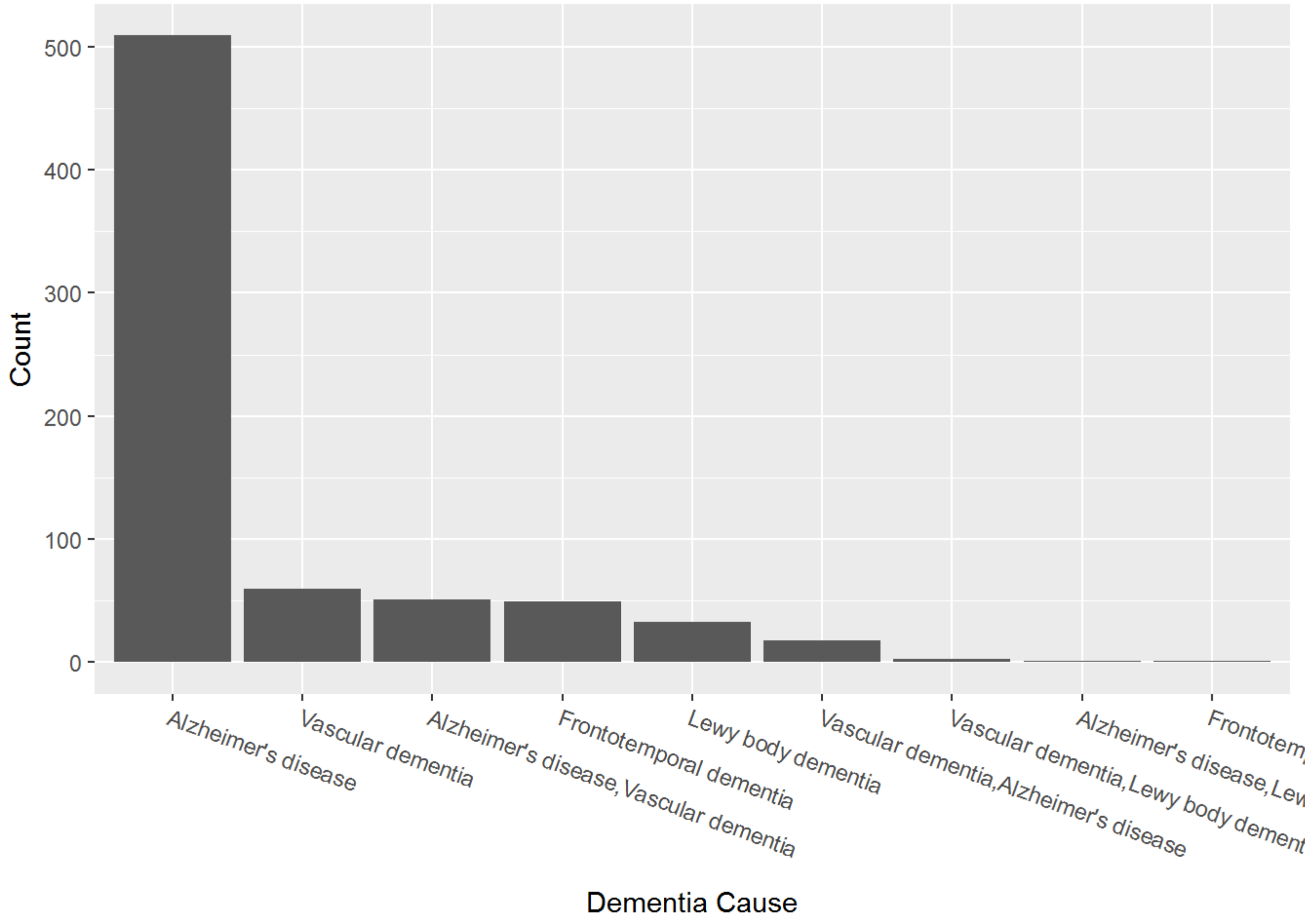

Criteria for Mild Cognitive Impairment

Value	Percent	Count	Male	Female
NA	60.2	1178	460	718
Yes	39.6	774	354	420
No	0.2	4	2	2

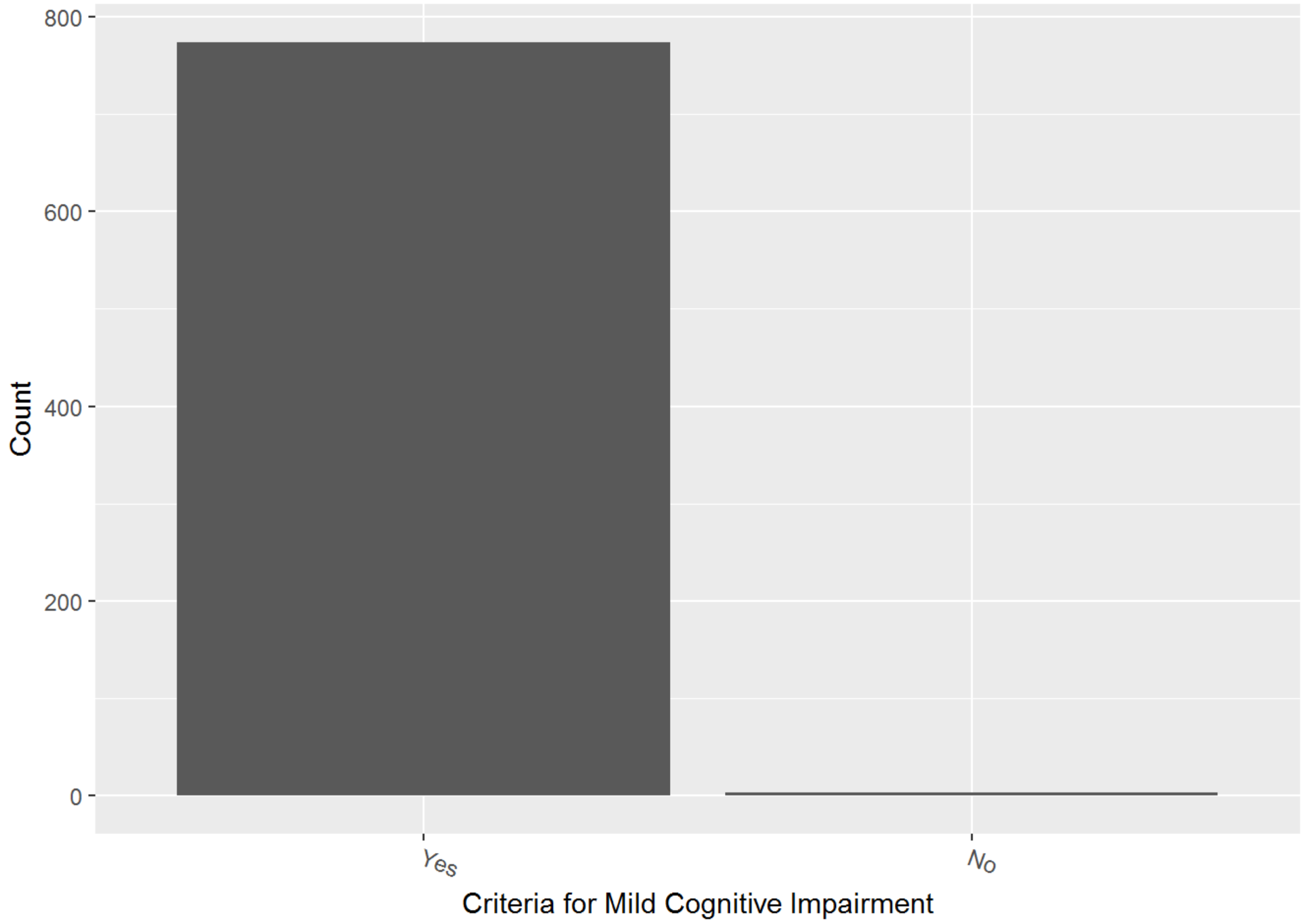

Mild Cognitive Impairment Subtype

Value	Percent	Count	Male	Female
Amnestic type	27.8	543	253	290
multi-domain	19.5	382	180	202
single domain	19.3	378	166	212
Non-amnestic type	11.1	217	93	124
Executive impairment	10.4	204	85	119
Visual Spatial impairment	6.5	127	59	68
Language impairment	4.3	84	28	56

Memory

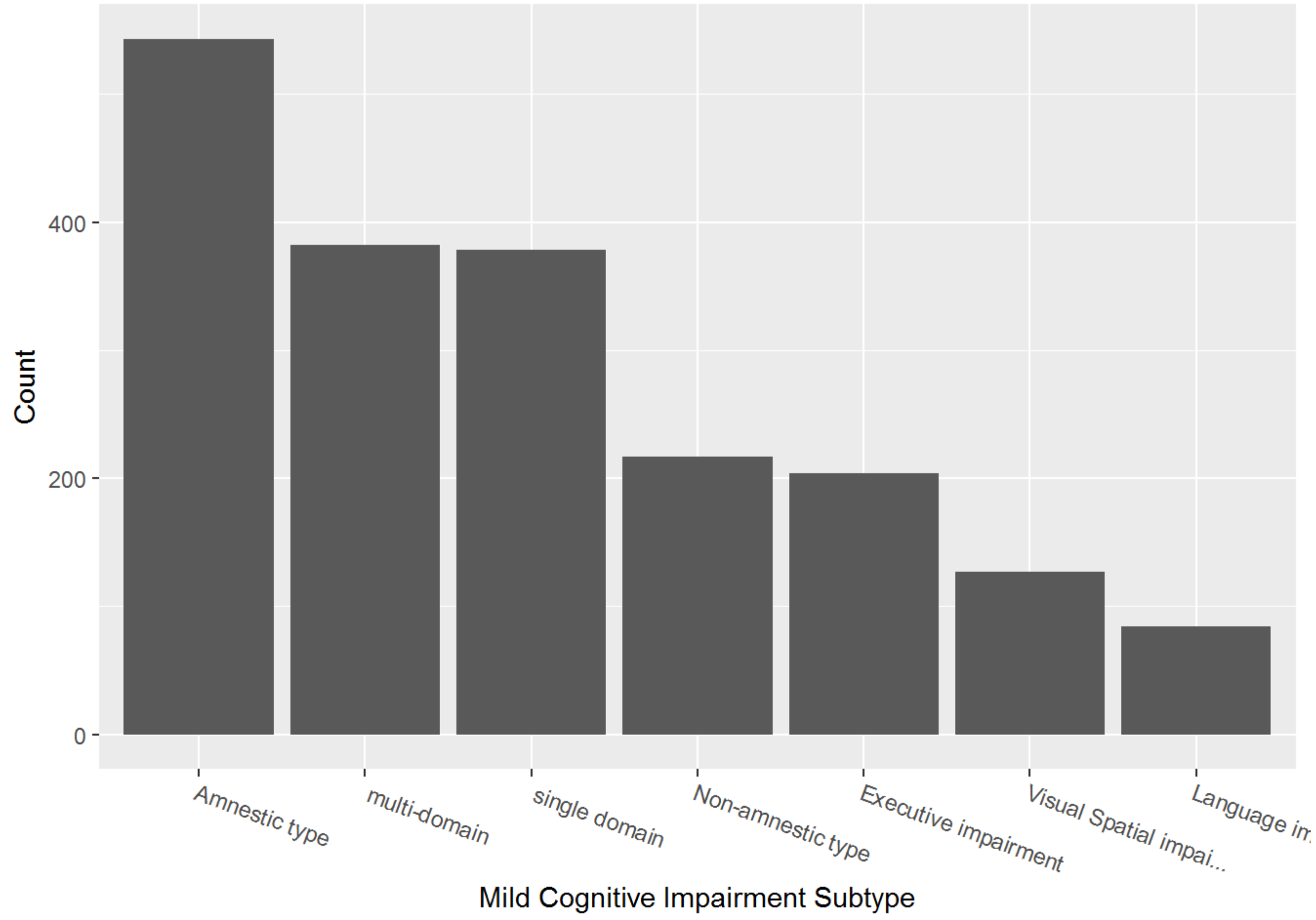
400
200
0
Count
Amnestic type
multi-domain
single domain
Non-amnestic type
Executive impairment
Visual Spatial impai...
Language im
Mild Cognitive Impairment Subtype

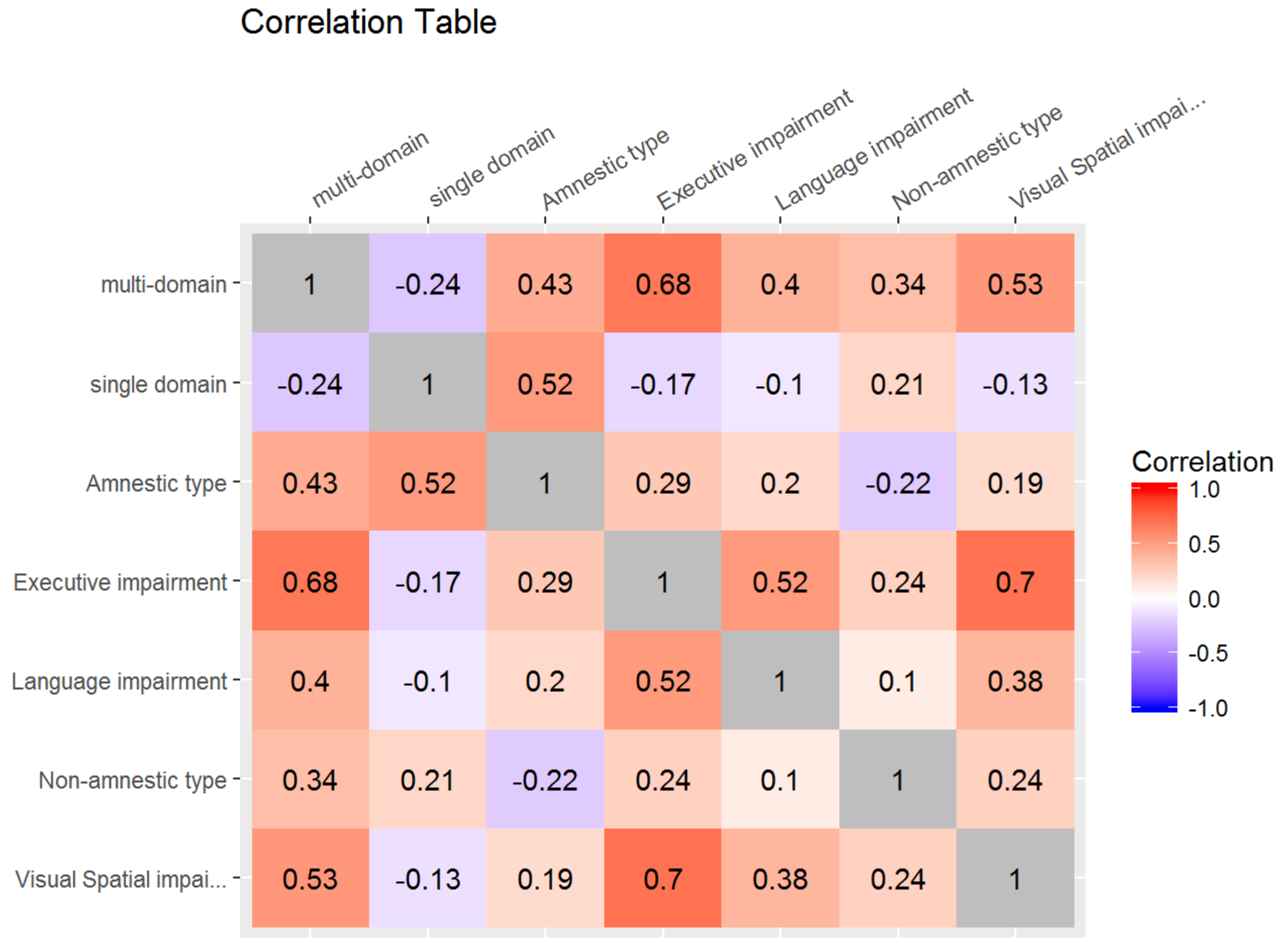
Correlation Table
multi-domain
single domain
Amnestic type
Executive impairment
Language impairment
Non-amnestic type
Visual Spatial impai...
multi-domain 1 -0.24 0.43 0.68 0.4 0.34 0.53
single domain -0.24 1 0.52 -0.17 -0.1 0.21 -0.13
Amnestic type 0.43 0.52 1 0.29 0.2 -0.22 0.19
Executive impairment 0.68 -0.17 0.29 1 0.52 0.24 0.7
Language impairment 0.4 -0.1 0.2 0.52 1 0.1 0.38
Non-amnestic type 0.34 0.21 -0.22 0.24 0.1 1 0.24
Visual Spatial impai... 0.53 -0.13 0.19 0.7 0.38 0.24 1
Correlation
1.0
0.5
0.0
-0.5
-1.0

Functional ASMT Staging Tool

Value	Percent	Count	Male	Female
Stage 1	0.4	8	3	5
Stage 2	12.6	247	110	137
Stage 3	27.5	537	248	289
Stage 4	40.5	793	321	472
Stage 5	6.9	134	48	86
Stage 6a	1.7	33	10	23
Stage 6b	3.5	69	16	53
Stage 6c	1.8	36	14	22
Stage 6d	1.1	22	10	12
Stage 6e	1.6	31	12	19
Stage 7a	0.8	15	7	8
Stage 7c	0.4	8	4	4
Stage 7f	0.1	1	0	1
NA	1.1	22	13	9

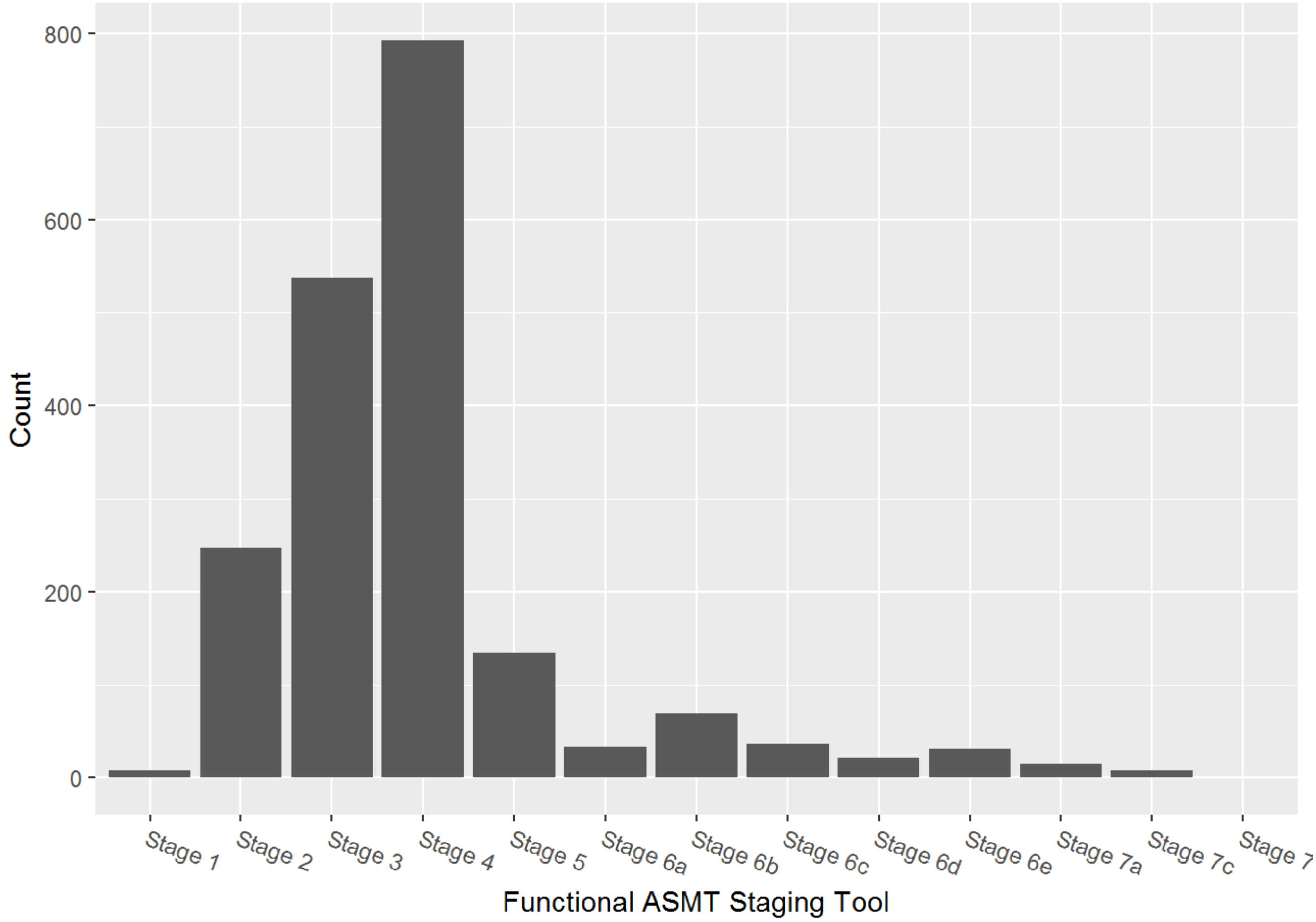

Barthel Interpretation

Value	Count	Male	Female
Independent	1594	686	908
Needs minimal help with ADL	181	65	116
Partially dependent	86	28	58

Memory

Very dependent	42	12	30
Totally dependent	11	4	7
NA	42	21	21

**Min**	**Median**	**Mean**	**Max**	**St.Dev**	**No.Data**
5	95	89.25	100	17.45	42

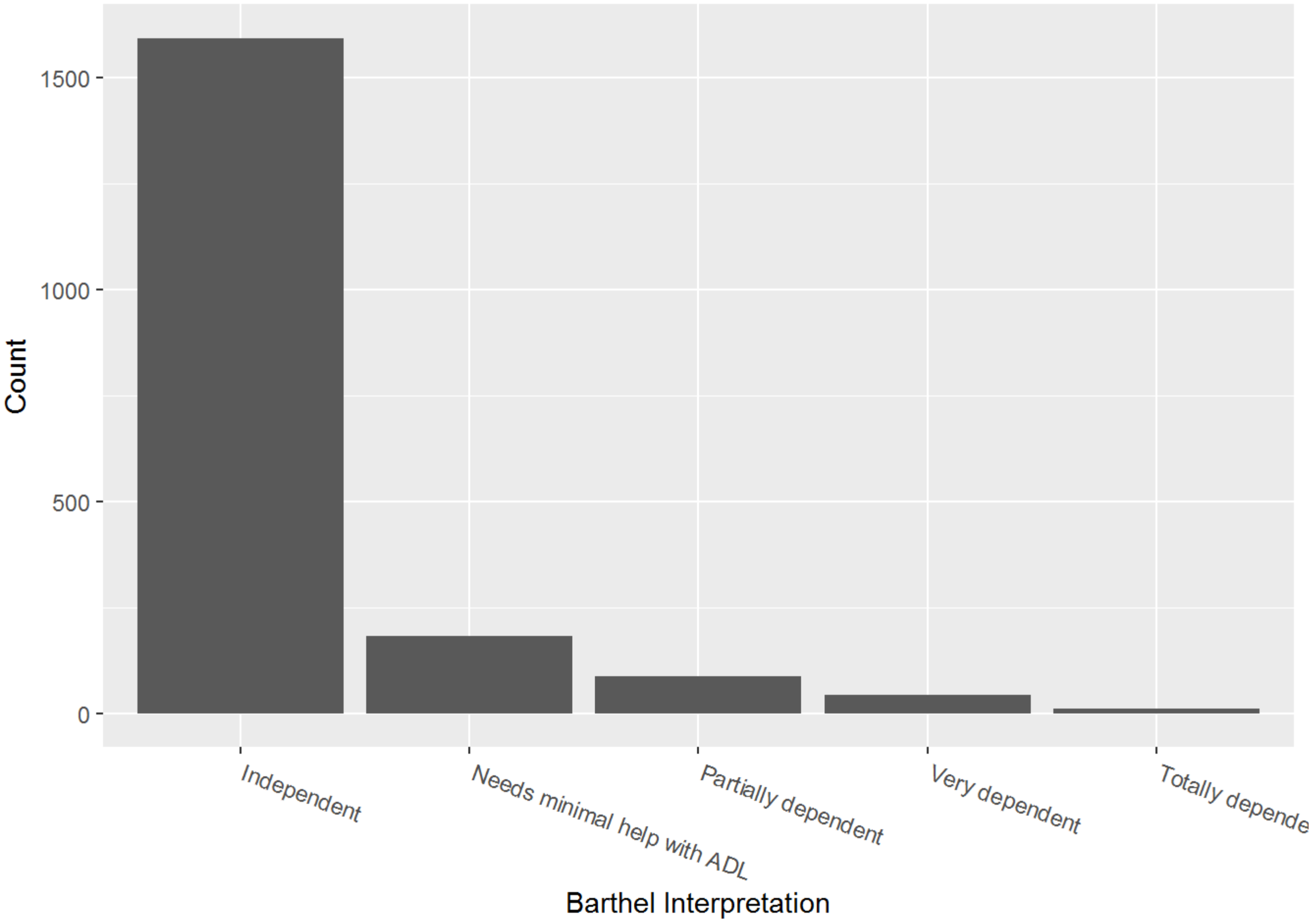

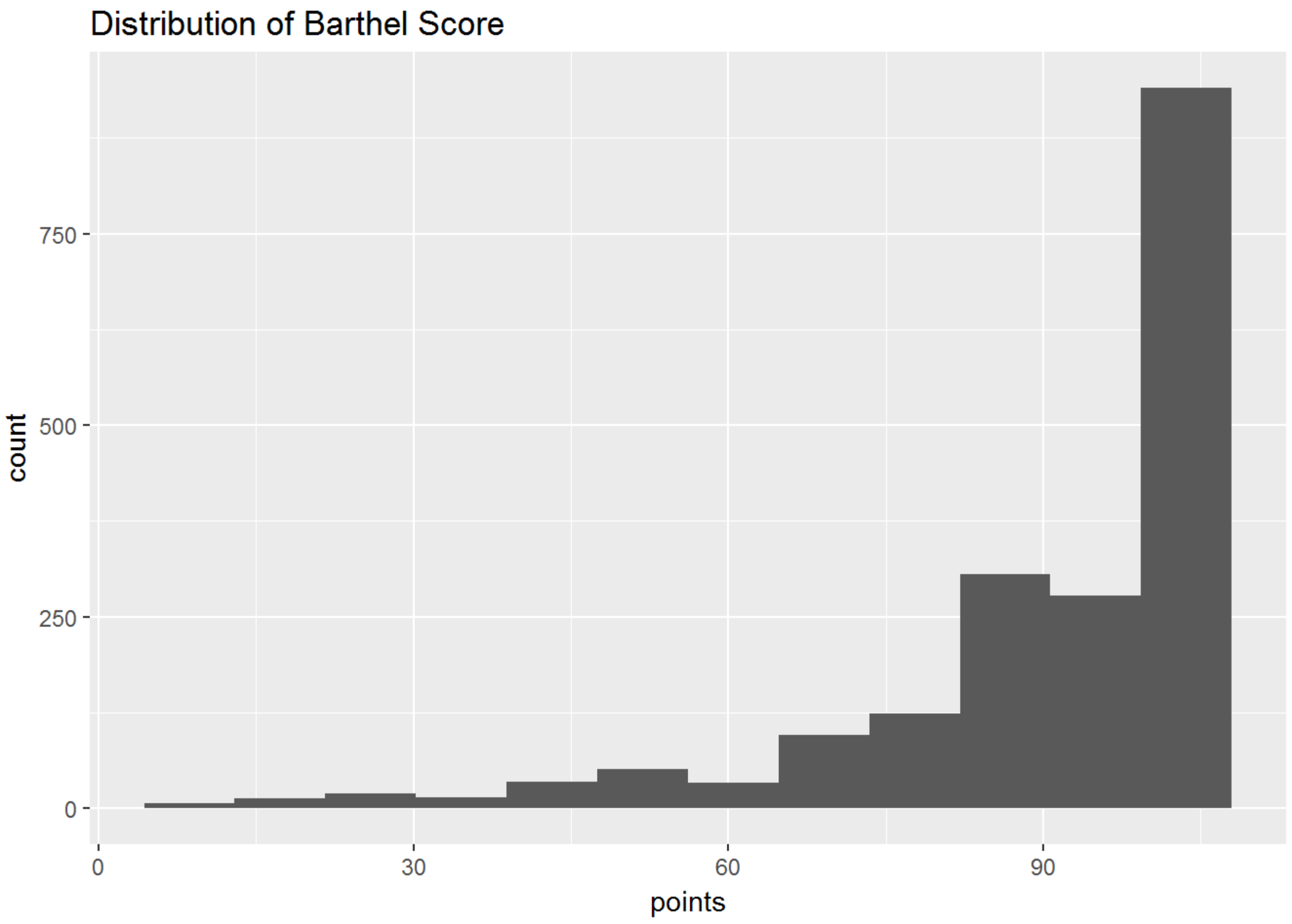
Distribution of Barthel Score
count
750
500
250
0
0
30
60
90
points

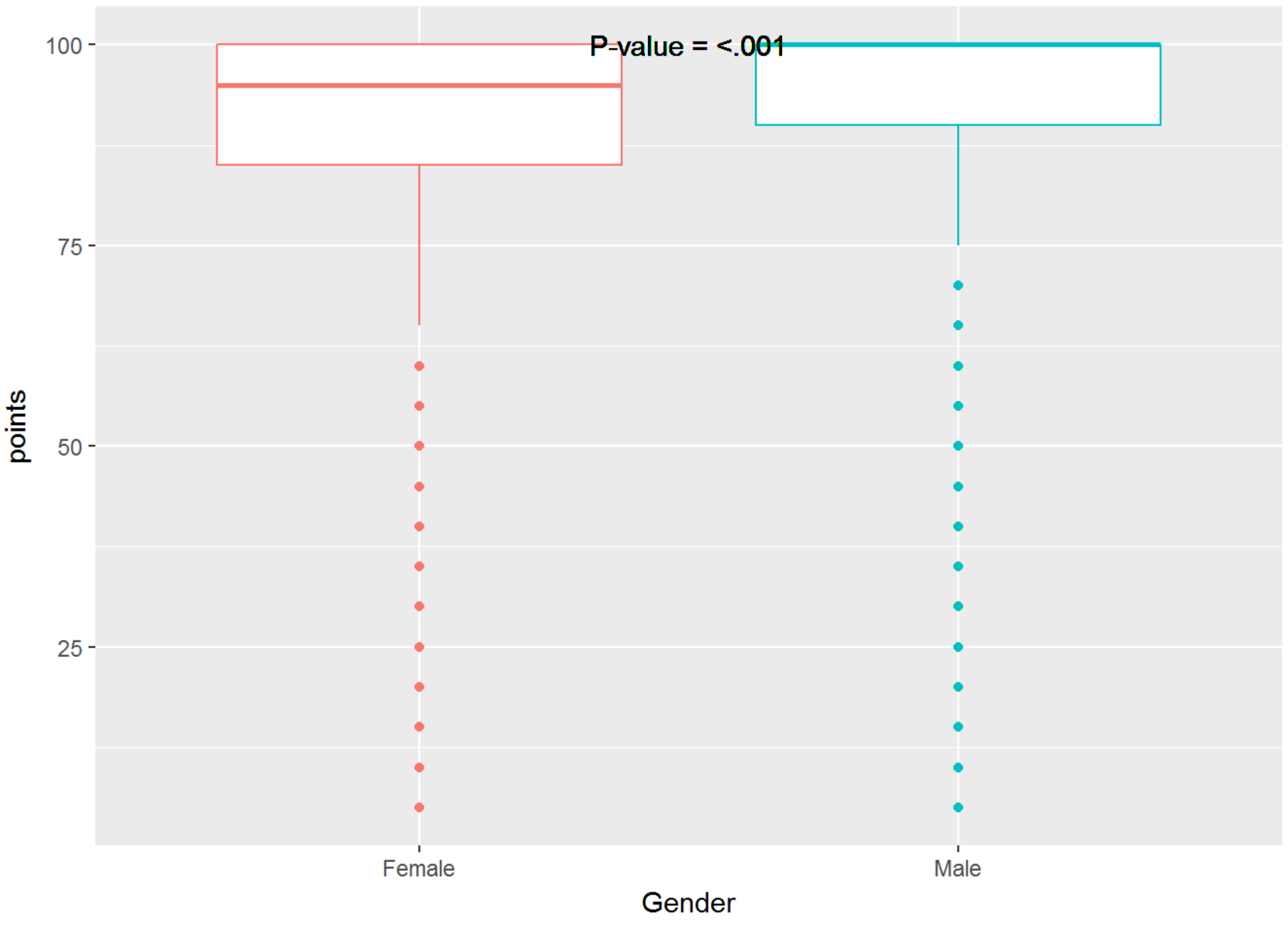
P-value = <.001
100
75
50
25
points
Female
Male
Gender

FAQ Interpretation

Value	Count	Male	Female
Does NOT indicate functional impairment	224	101	123
Does NOT indicate impaired function or possible cognitive impairment	640	291	349
Possible impaired function	296	112	184
Impaired function and possible cognitive impairment	737	288	449
NA	59	24	35

Min	Median	Mean	Max	St.Dev	No.Data
0	10	11.21	30	8.969	59

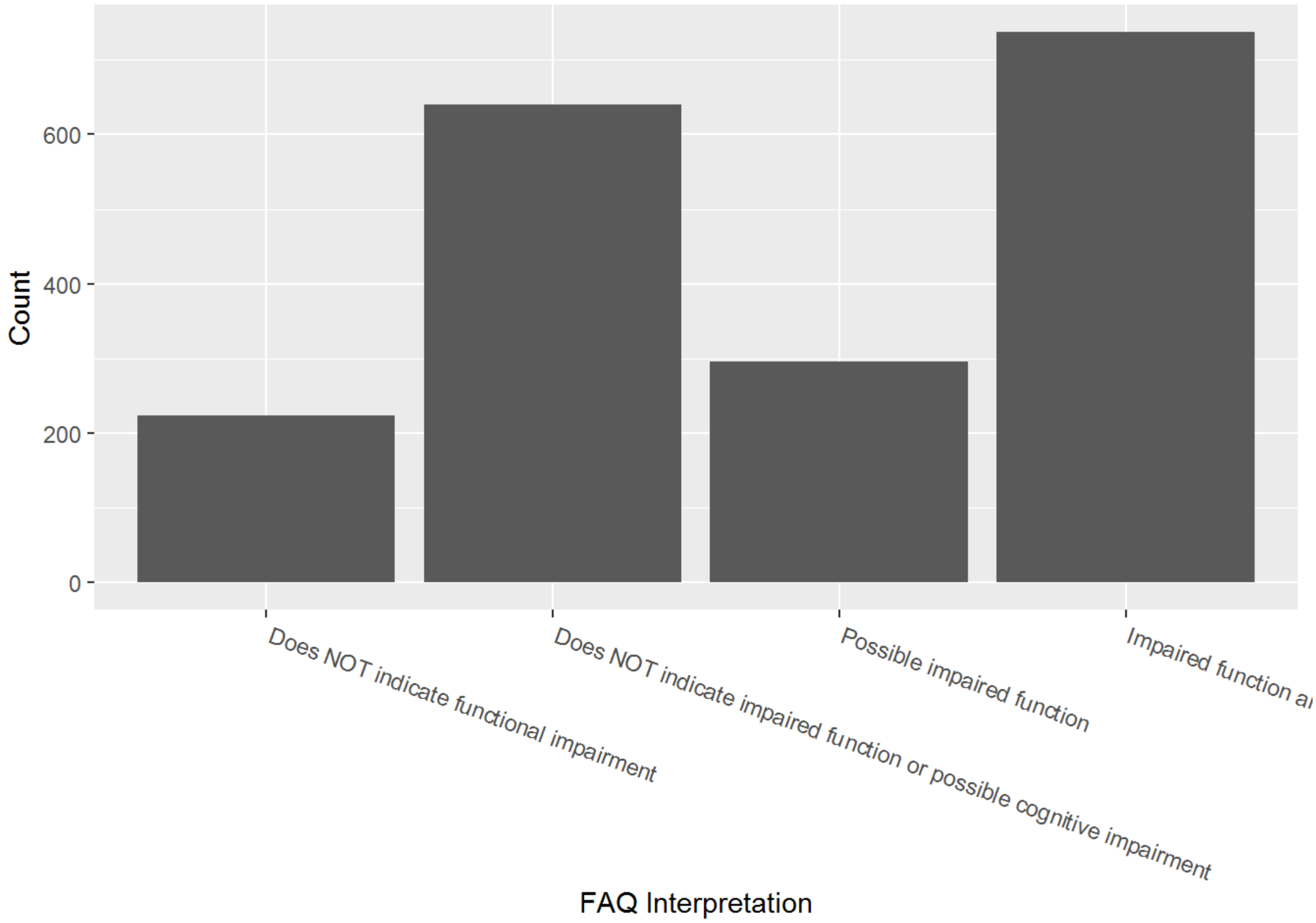

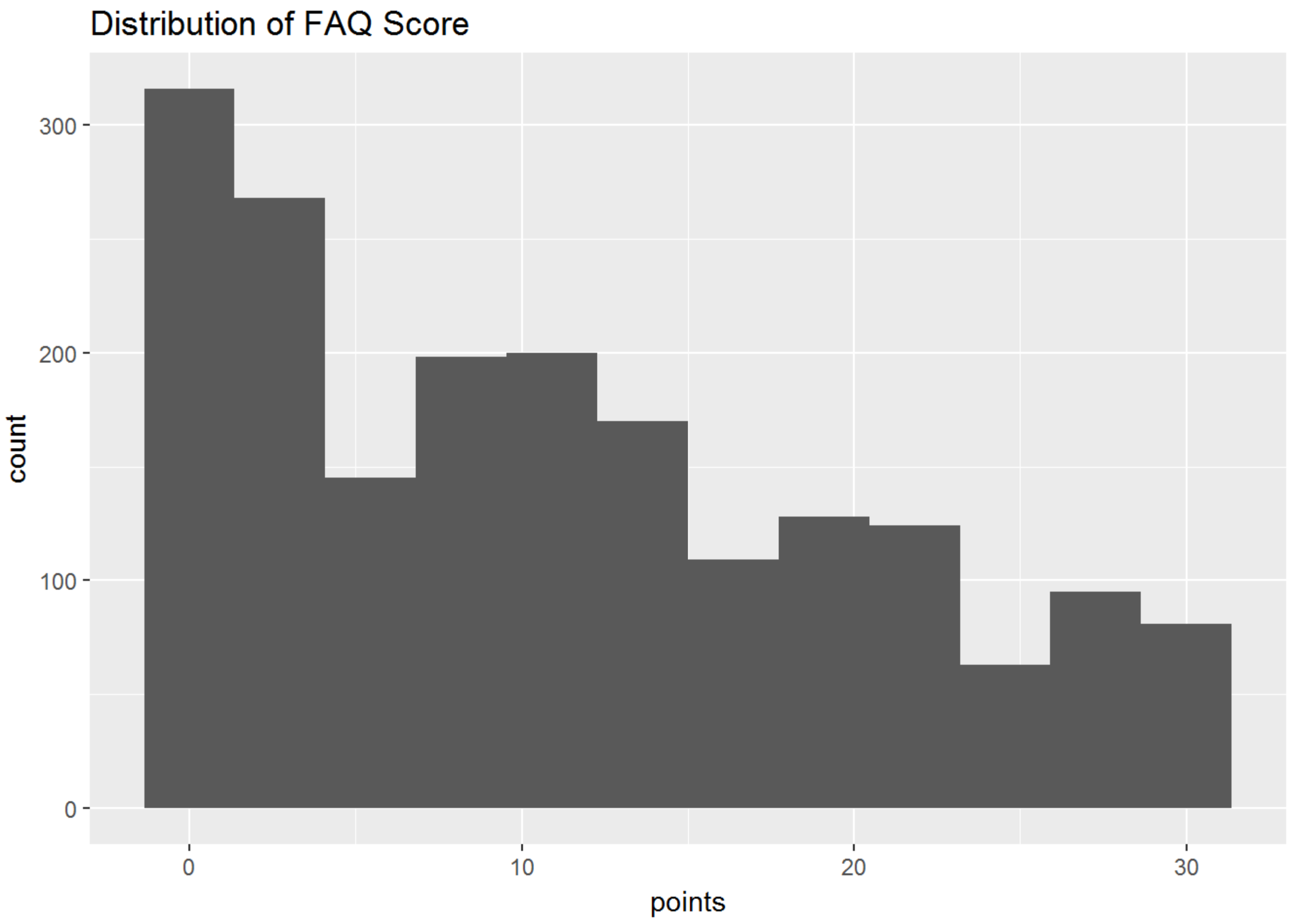
Distribution of FAQ Score
count
300
200
100
0
0
10
20
30
points

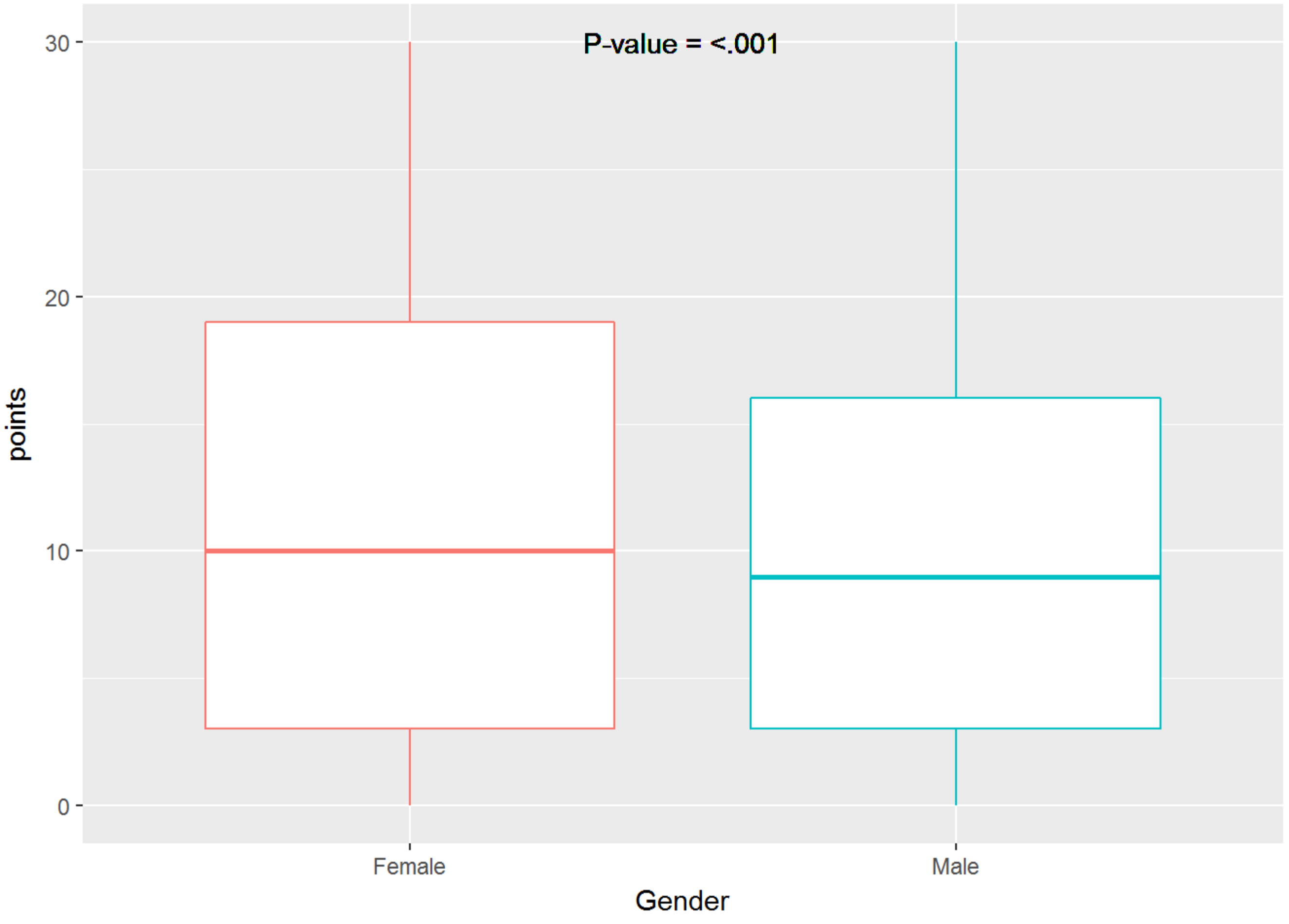
P-value = <.001
points
30
20
10
0
Female
Male
Gender

GDS Interpretation

Value	Count	Male	Female
Screens Negative for Depression	1378	604	774
Screens Positive for Depression	478	175	303
NA	100	37	63

Min	Median	Mean	Max	St.Dev	No.Data
0	3	3.917	15	3.491	100

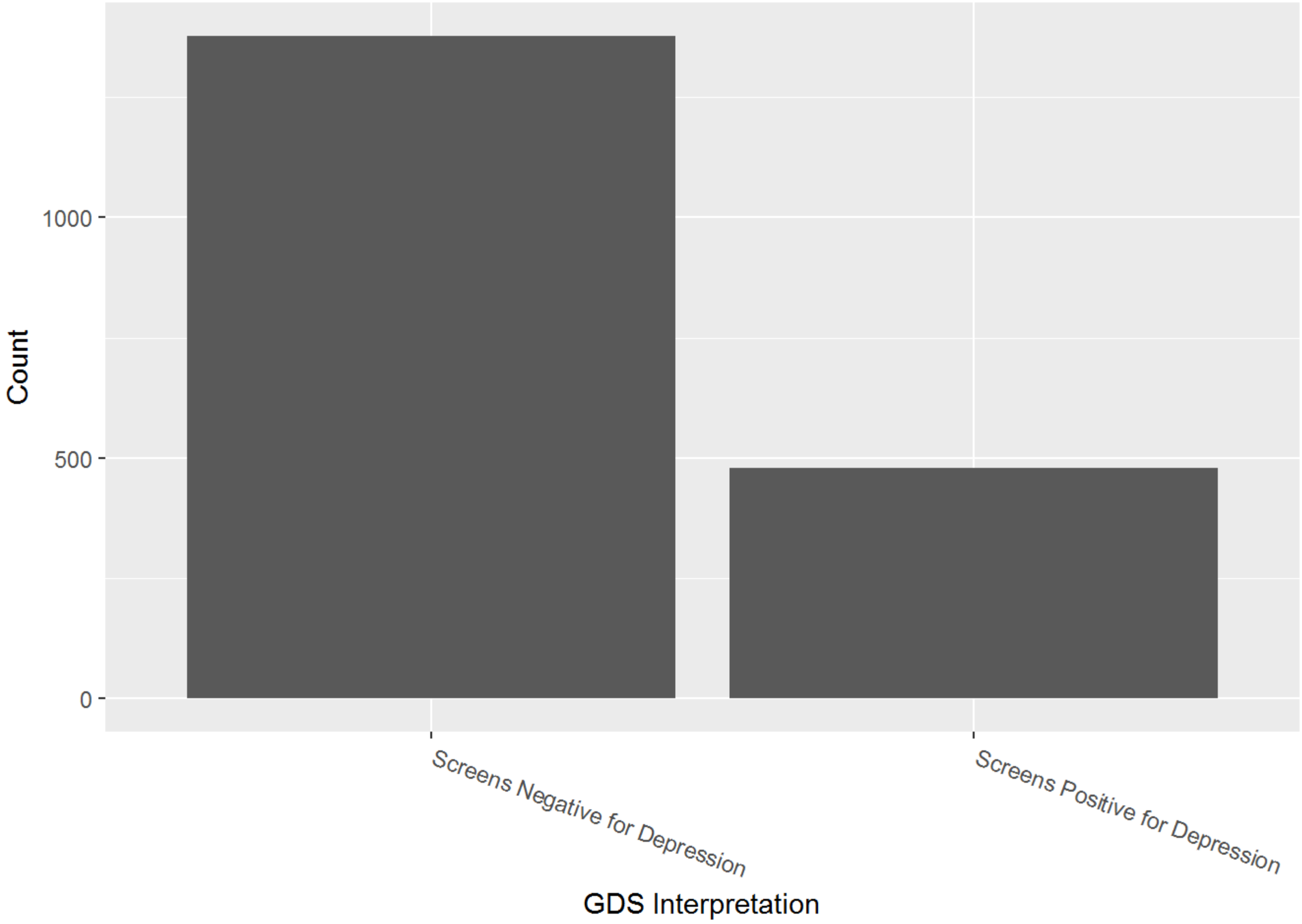

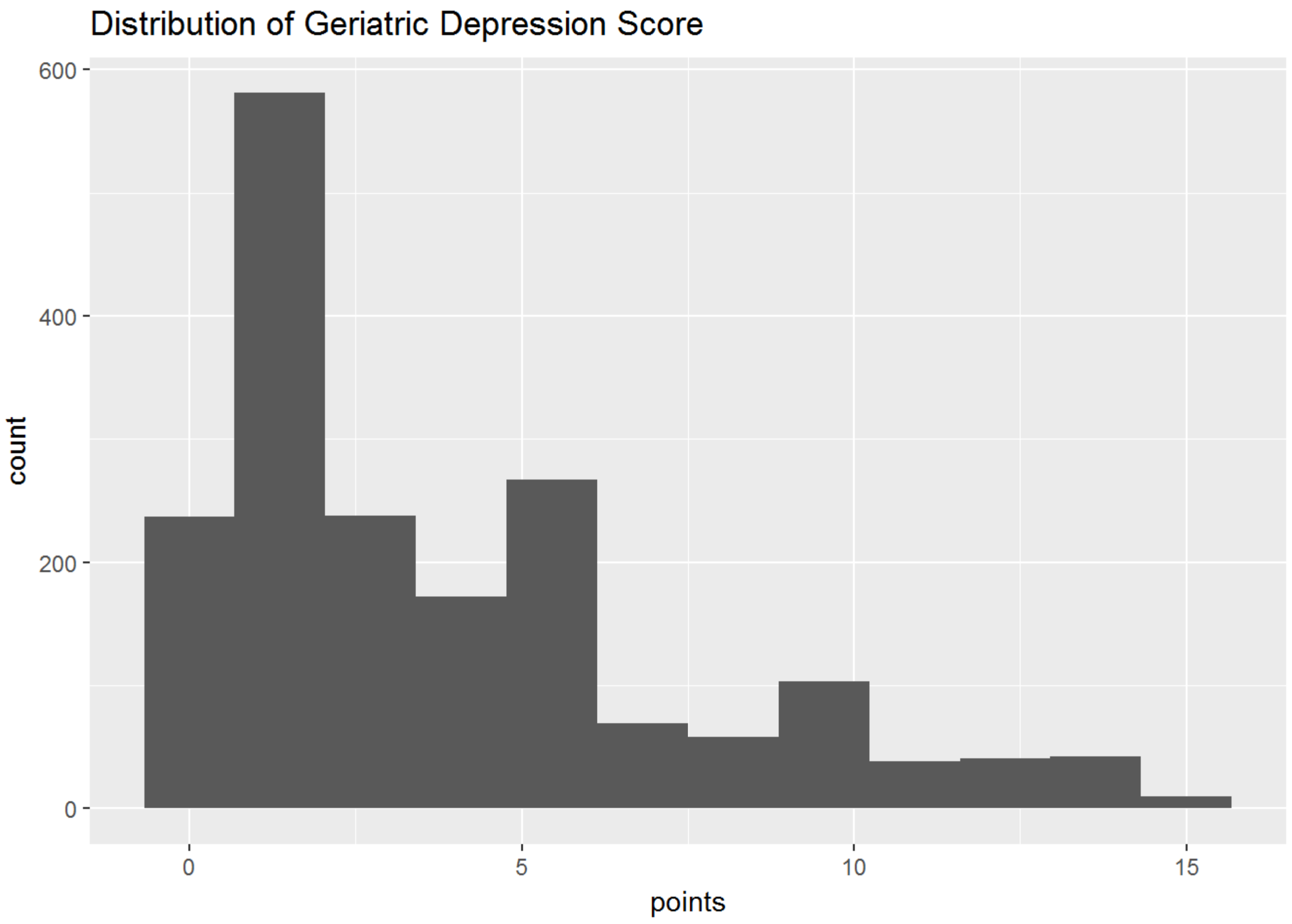
Distribution of Geriatric Depression Score
count
points
600
400
200
0
0
5
10
15

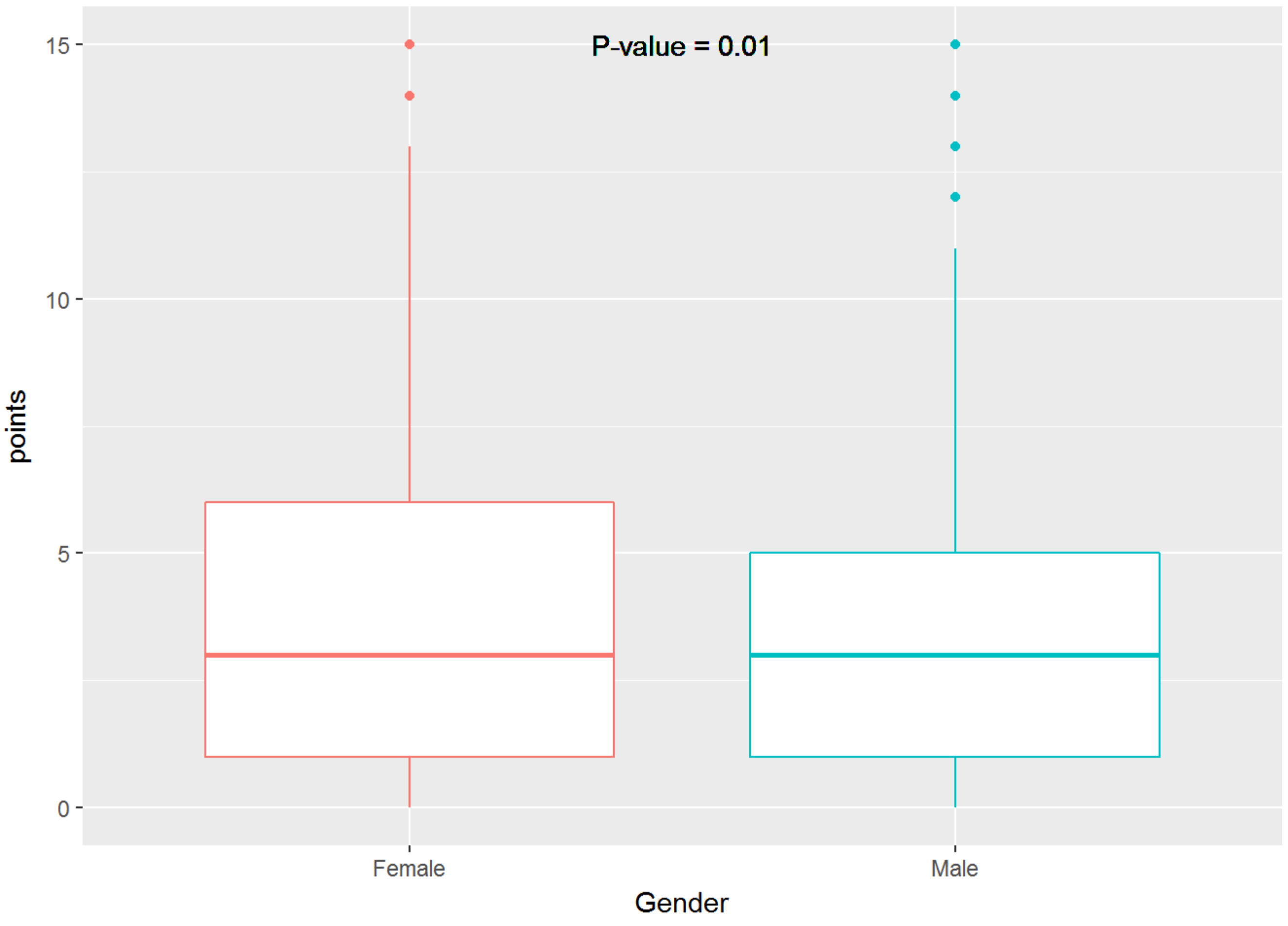
P-value = 0.01
points
Gender
Female
Male
15
10
5
0

MMSE Interpretation

Value	Count	Male	Female
Normal	860	402	458
Possible Cognitive Impairment	833	319	514
NA	263	95	168

Min	Median	Mean	Max	St.Dev	No.Data
6	24	22.78	29	4.372	263

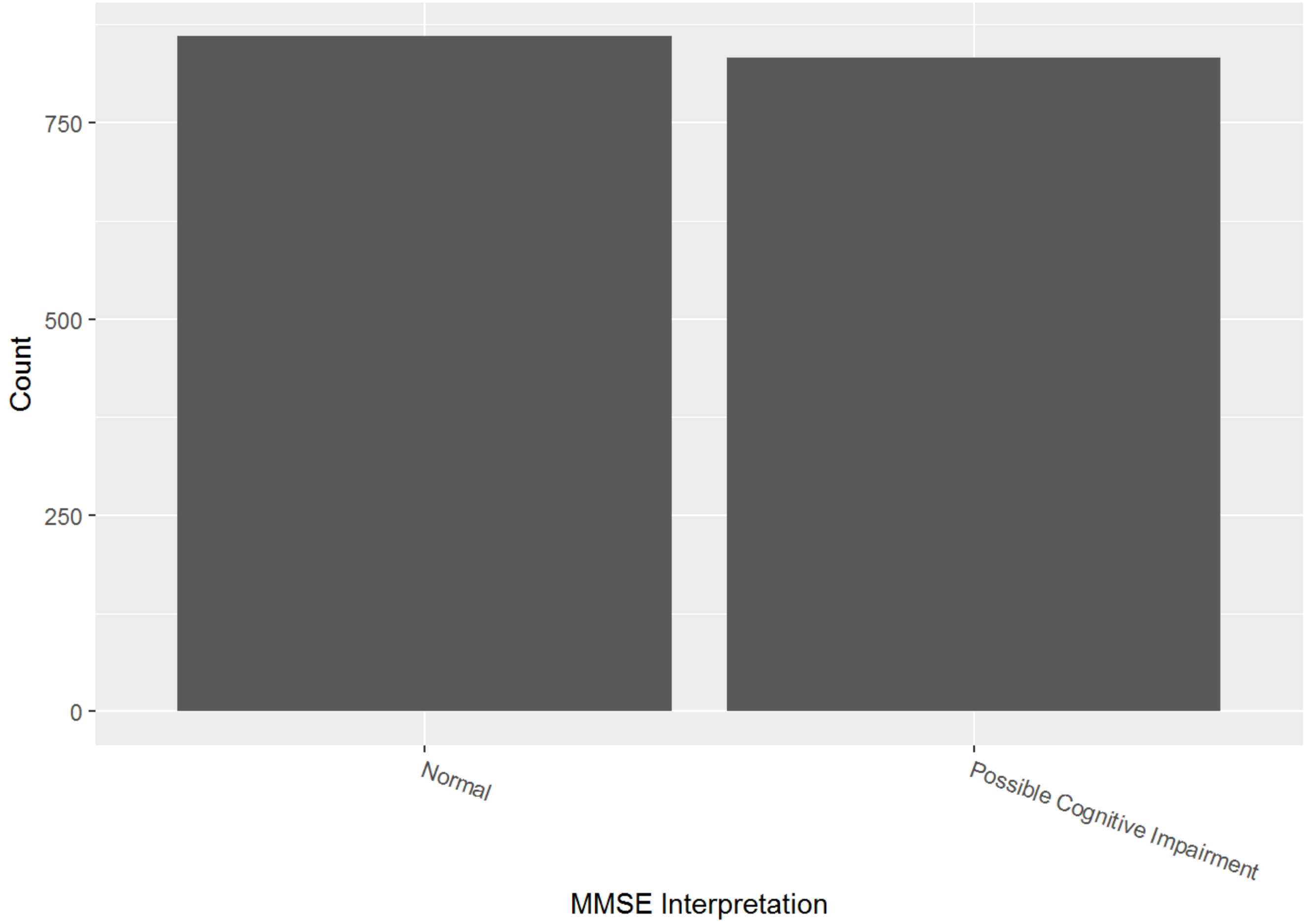

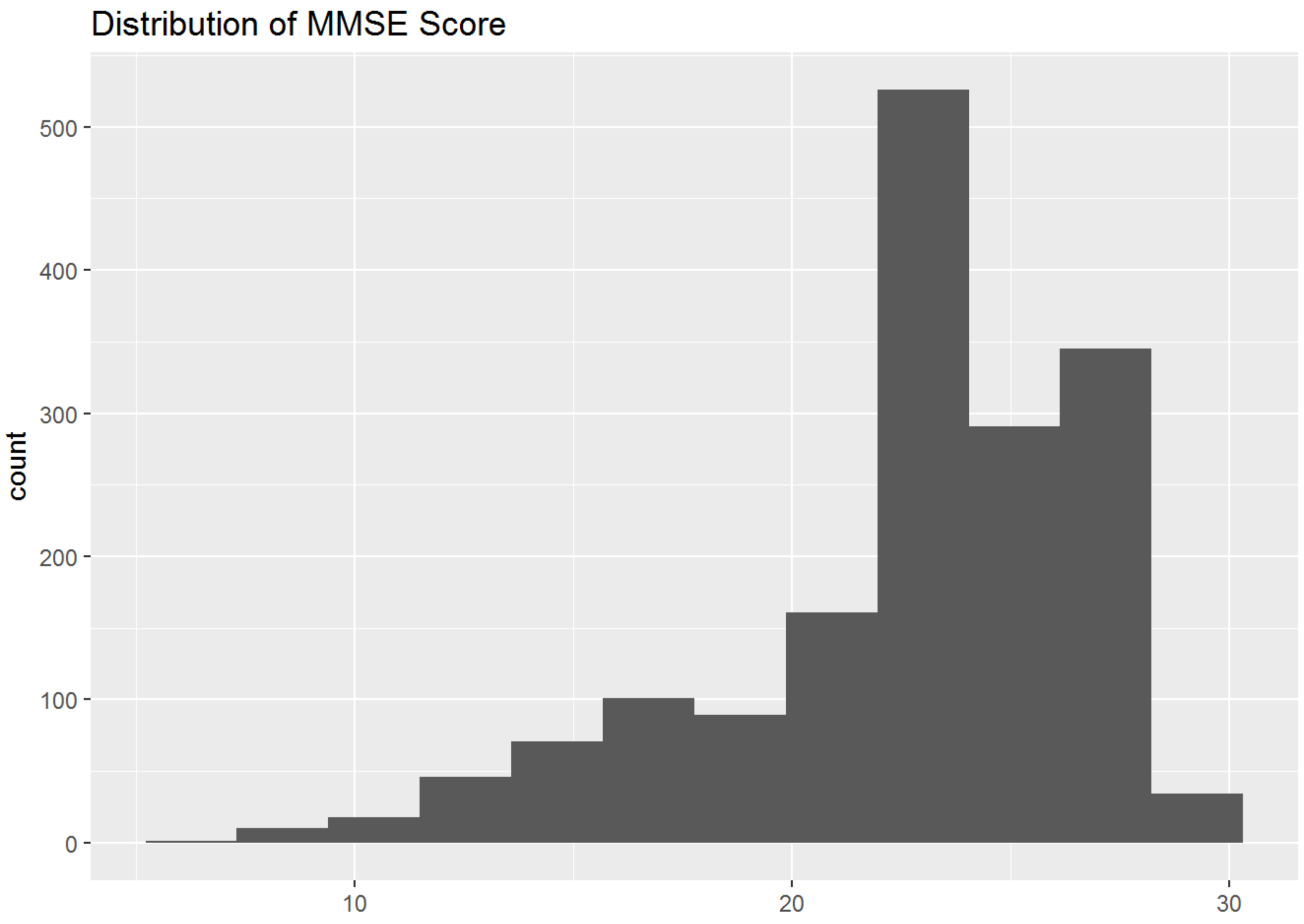

Distribution of MMSE Score
count
500
400
300
200
100
0
10
20
30

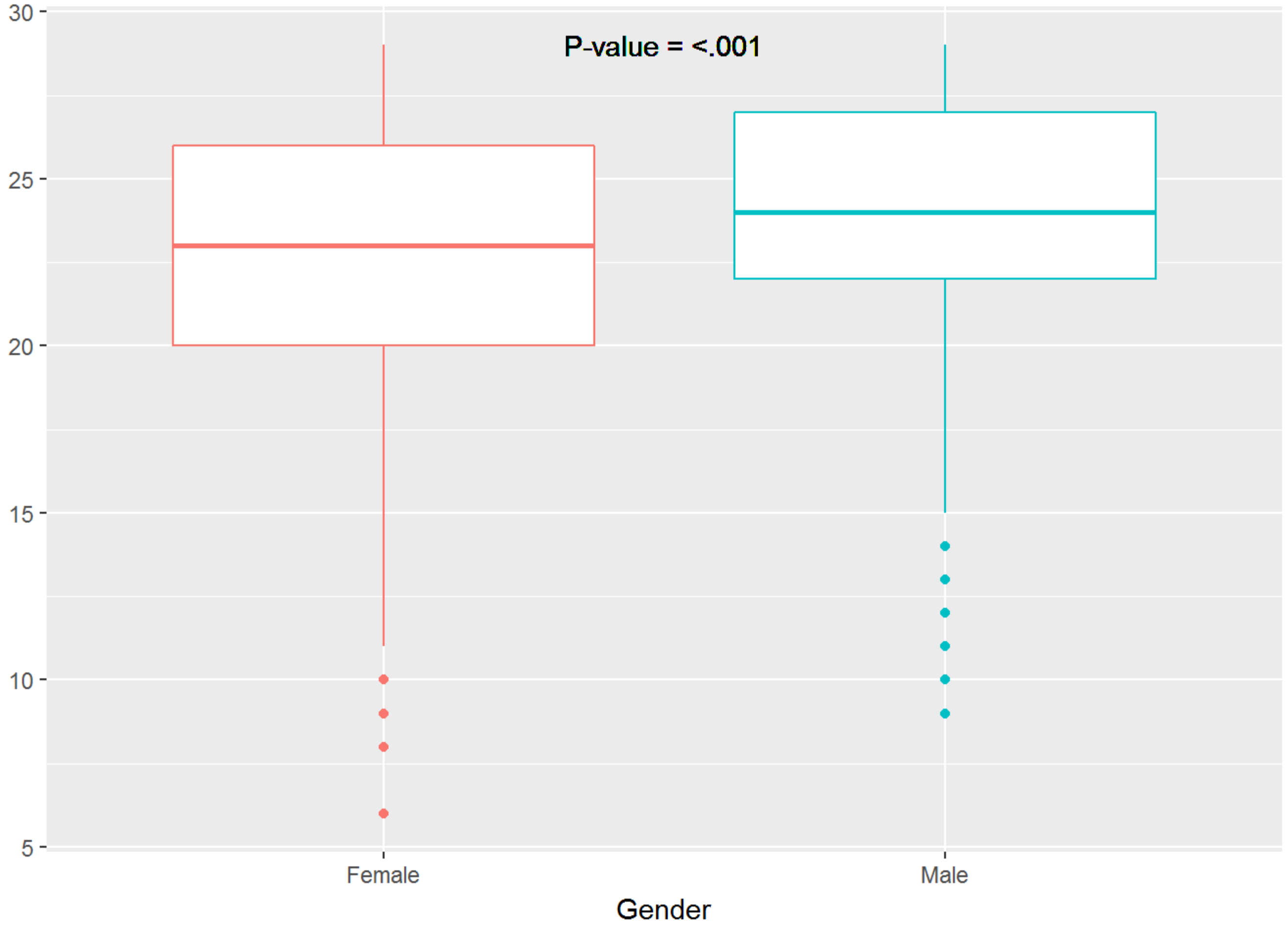

P-value = <.001
30
25
20
15
10
5
Female
Male
Gender

Family History of ALS

Value	Percent	Count	Male	Female
No	99.4	1944	809	1135
Yes	0.6	12	7	5

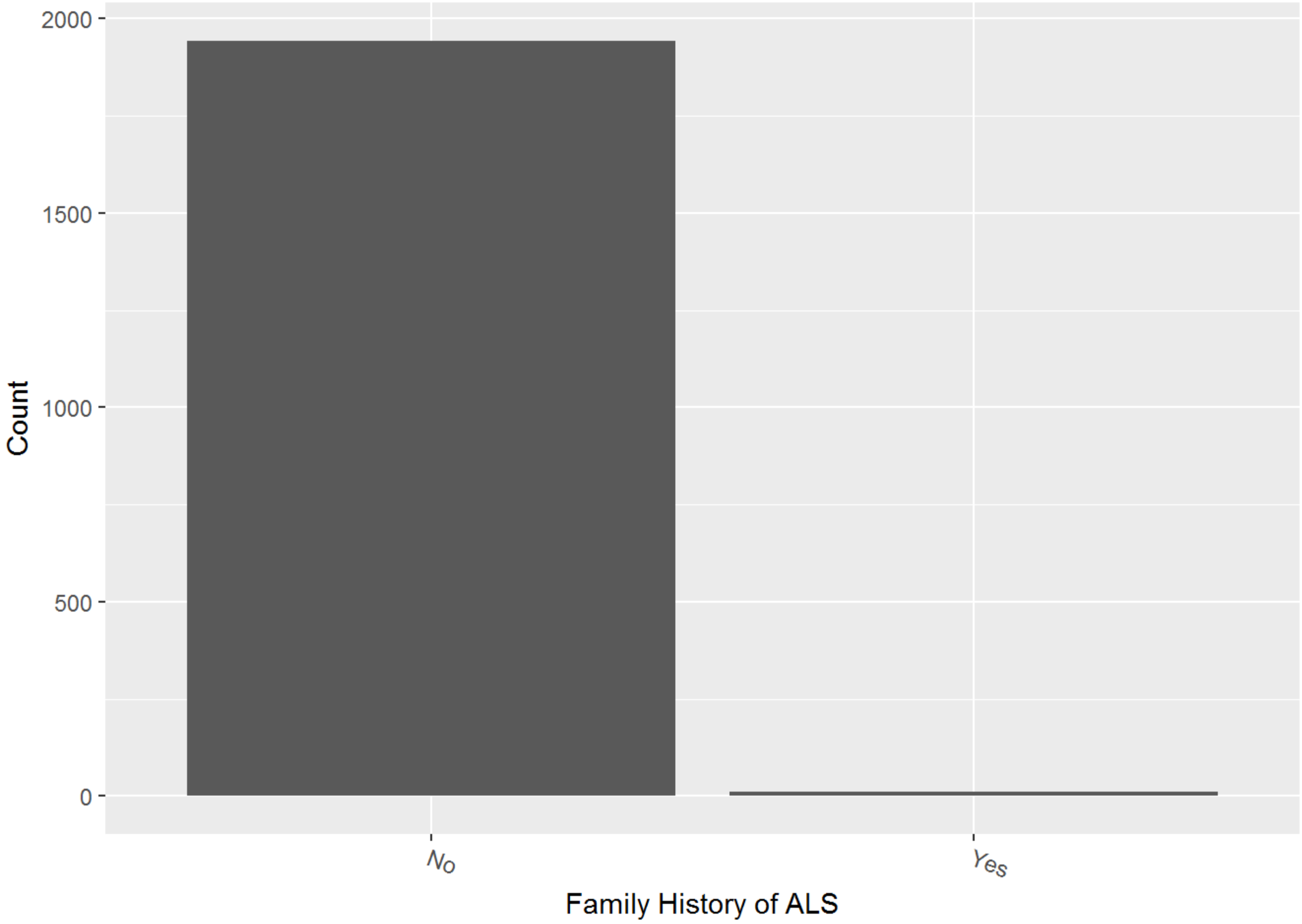

Family Members with History of ALS

Value	Percent	Count	Male	Female
BRO	0.2	4	2	2
FA	0.2	3	1	2
MO	0.2	4	2	2
DA	0.1	1	1	0
MA	0.1	1	1	0
MU	0.1	1	0	1

Memory

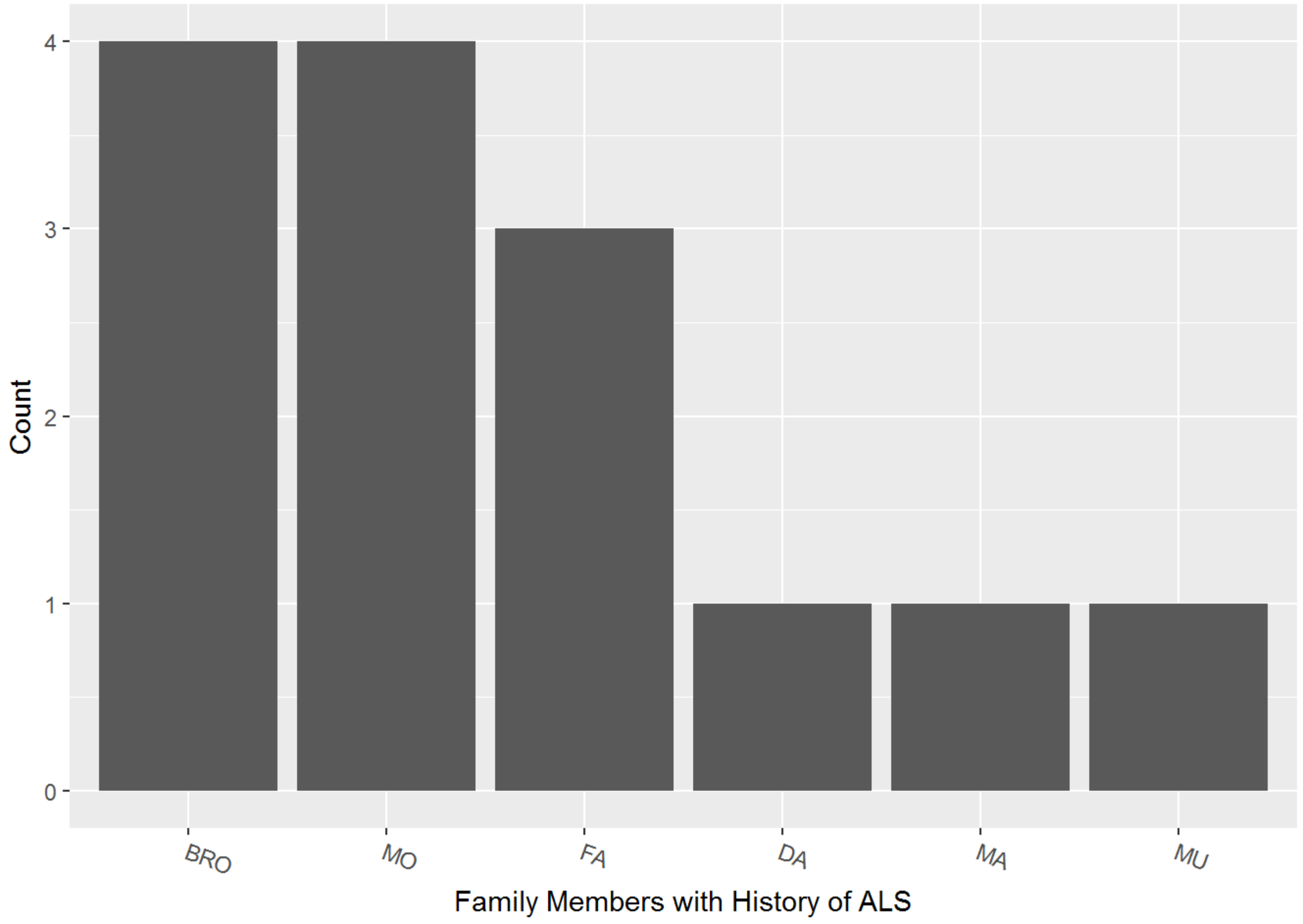

Correlation Table

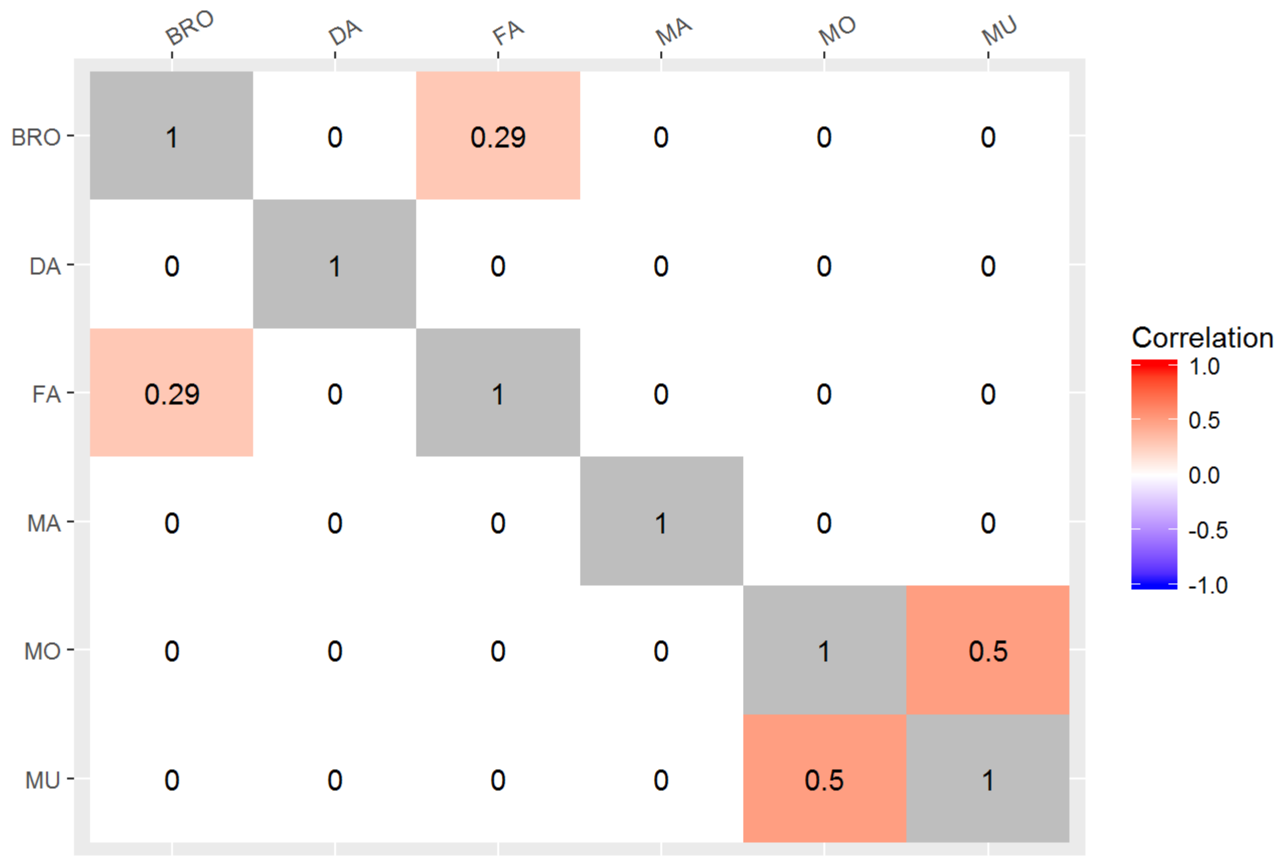

Family History of Epilepsy

Value	Percent	Count	Male	Female
No	98.8	1933	806	1127
Yes	1.2	23	10	13

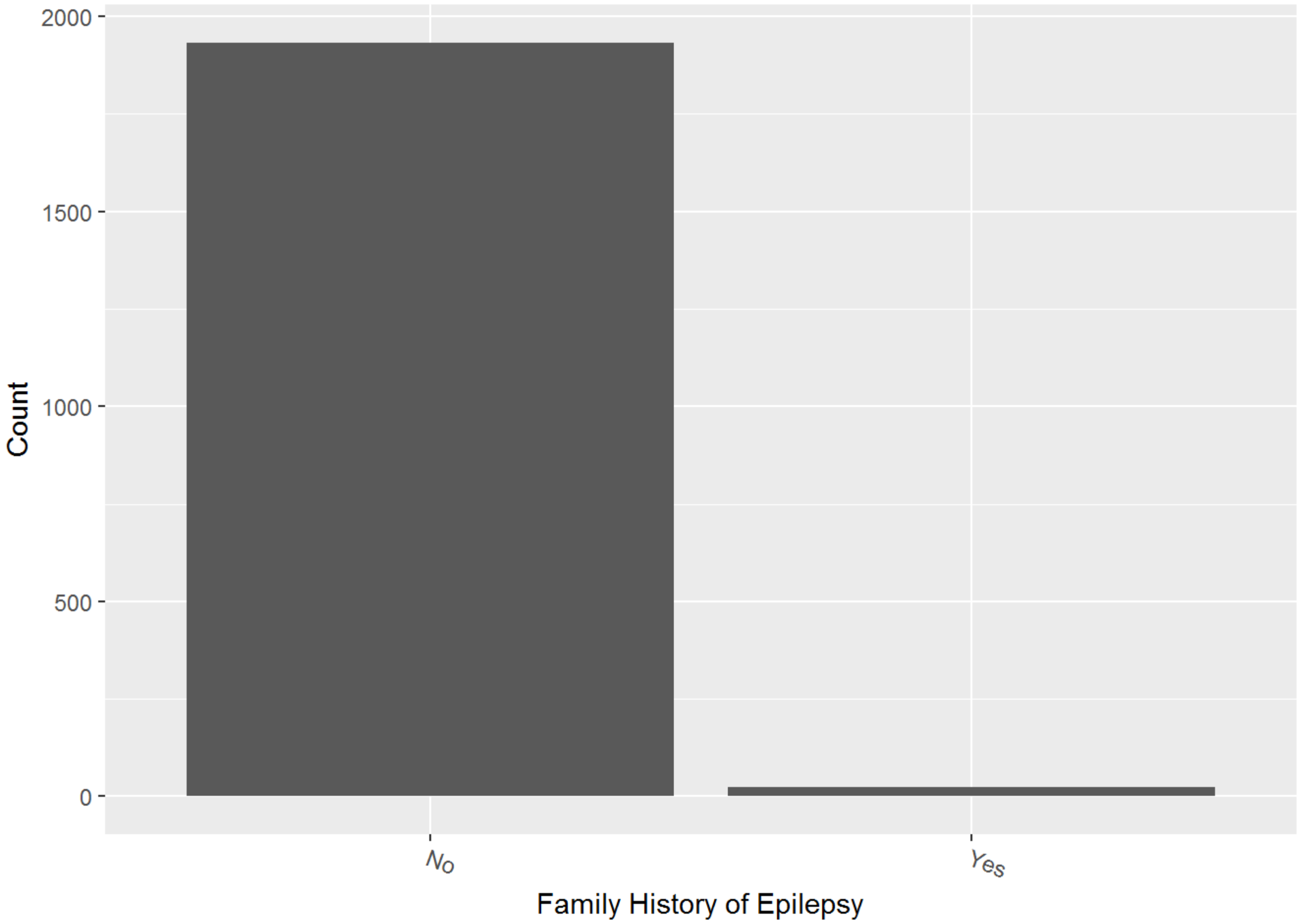

Family Members with History of Epilepsy

Value	Percent	Count	Male	Female
SN	0.4	7	2	5
FA	0.2	3	2	1
MO	0.2	3	0	3
SIS	0.2	3	1	2
BRO	0.1	1	1	0
DA	0.1	2	1	1
OTH	0.1	1	0	1
PA	0.1	1	1	0
PC	0.1	1	1	0
PU	0.1	2	1	1

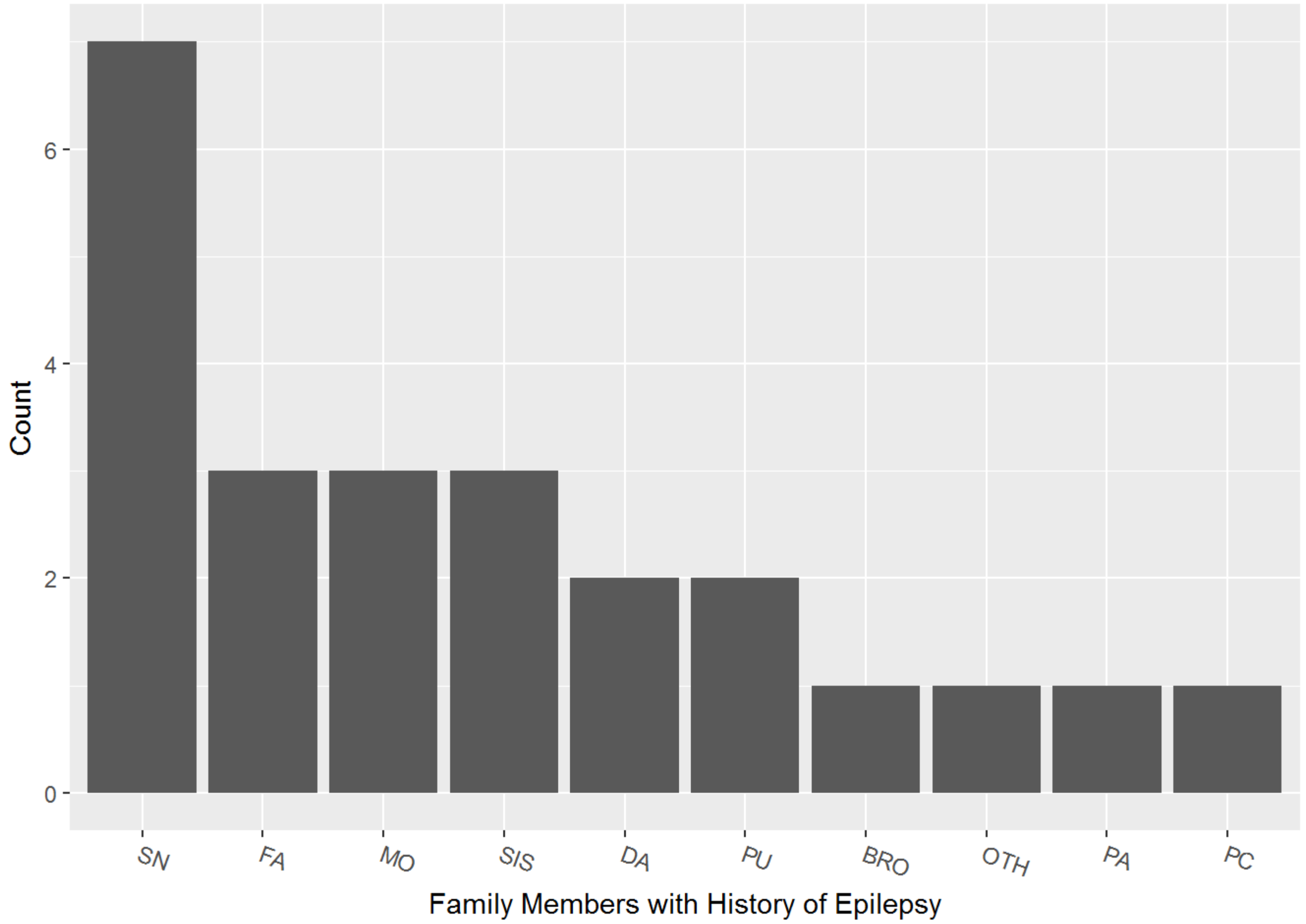

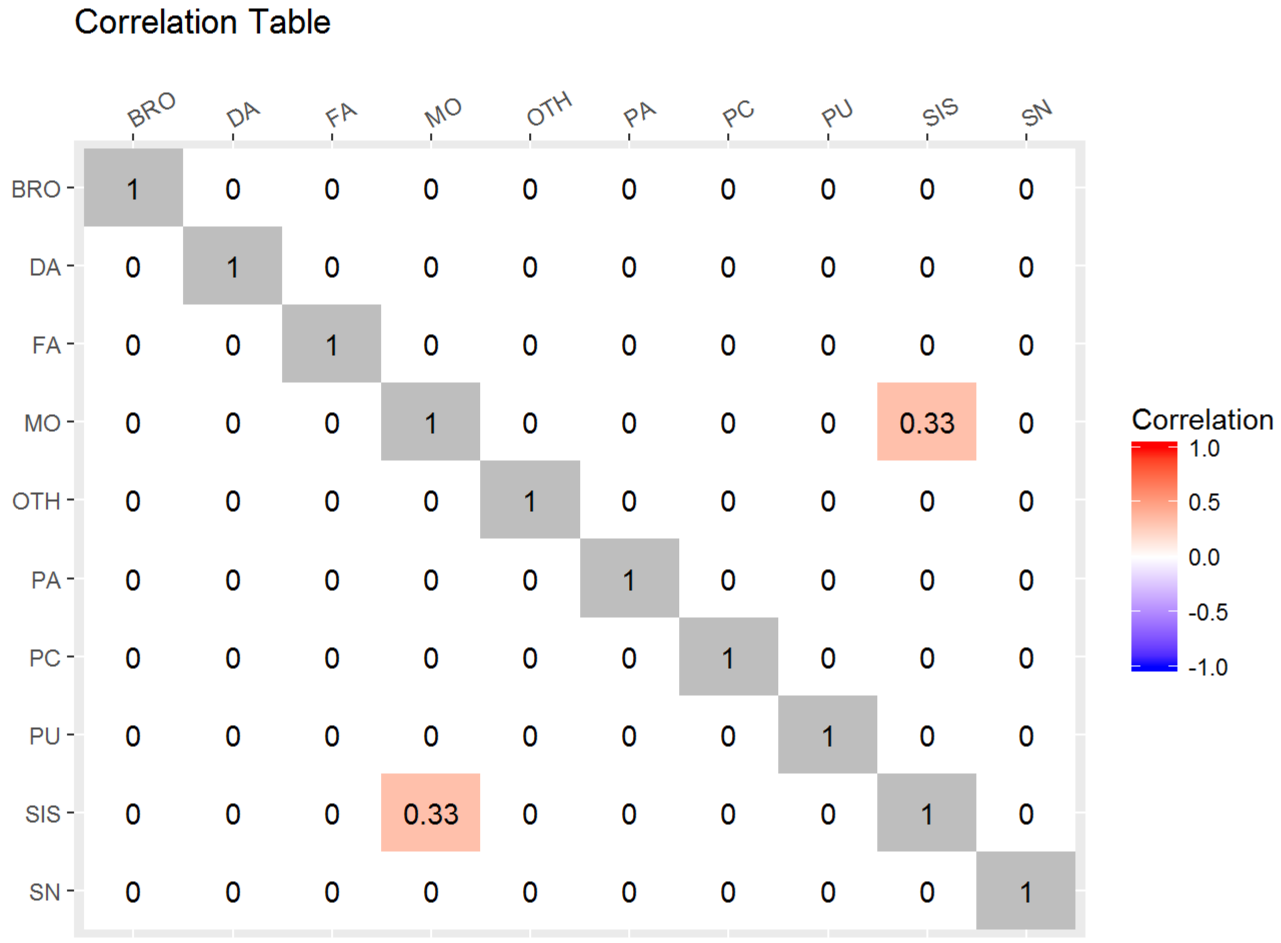

	BRO	DA	FA	MO	OTH	PA	PC	PU	SIS	SN
BRO	1	0	0	0	0	0	0	0	0	0
DA	0	1	0	0	0	0	0	0	0	0
FA	0	0	1	0	0	0	0	0	0	0
MO	0	0	0	1	0	0	0	0	0.33	0
OTH	0	0	0	0	1	0	0	0	0	0
PA	0	0	0	0	0	1	0	0	0	0
PC	0	0	0	0	0	0	1	0	0	0
PU	0	0	0	0	0	0	0	1	0	0
SIS	0	0	0	0.33	0	0	0	0	1	0
SN	0	0	0	0	0	0	0	0	0	1

Family History of Dementia

Value	Percent	Count	Male	Female
No	59.3	1159	482	677
Yes	40.7	797	334	463

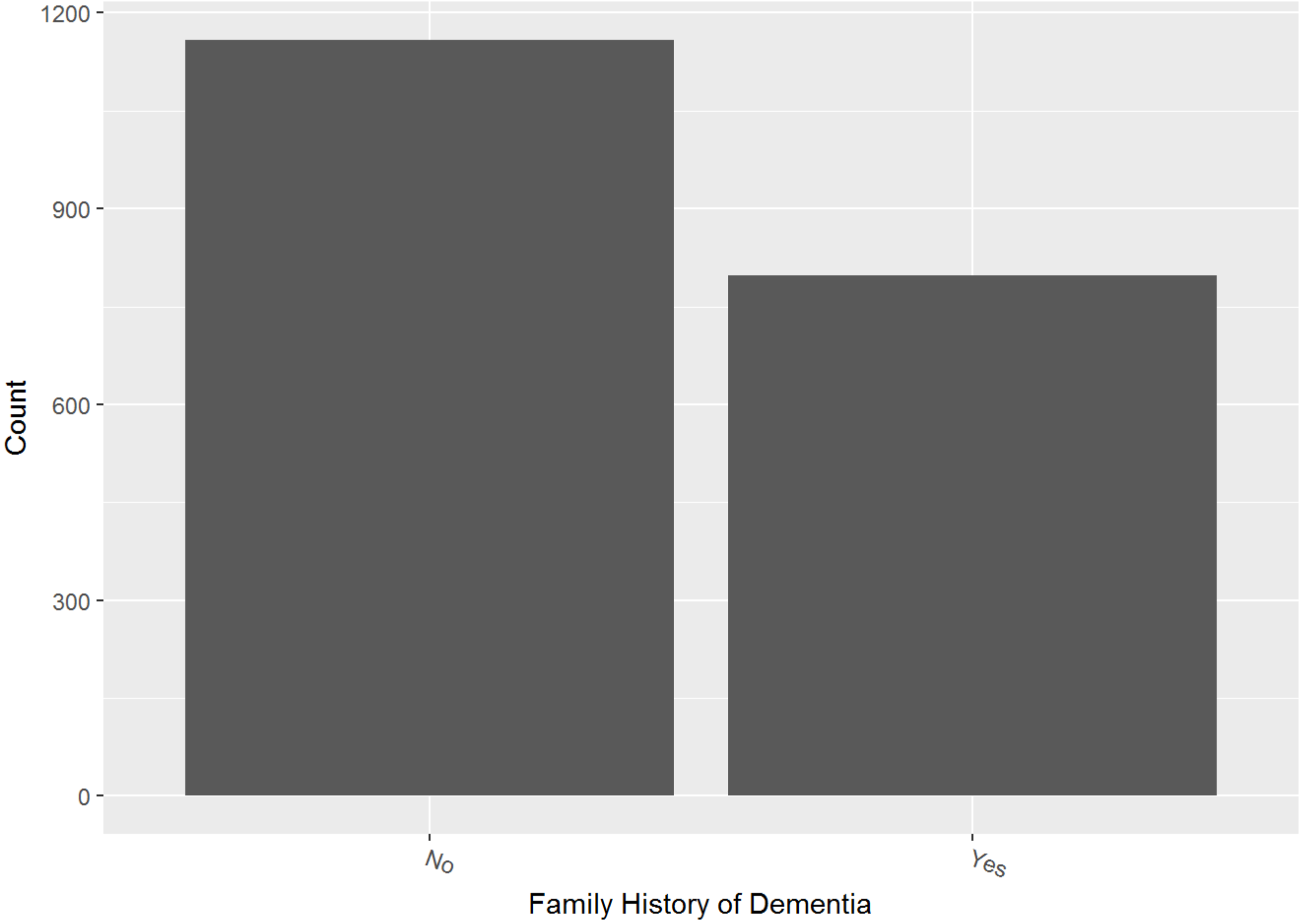

Family Members with History of Dementia

Value	Percent	Count	Male	Female
MO	19.9	389	161	228
FA	8.6	169	79	90
SIS	7.8	153	57	96
BRO	5.2	101	50	51
MA	3	58	16	42
MGM	2.5	48	15	33
PGM	1.9	37	17	20
PA	1.3	26	11	15
PU	1.1	22	14	8
MU	1	20	8	12
MGF	0.9	18	7	11
PGF	0.7	14	6	8
MC	0.5	9	7	2
PC	0.3	6	2	4
OTH	0.2	4	0	4

Memory

DA	0.1	1	0	1

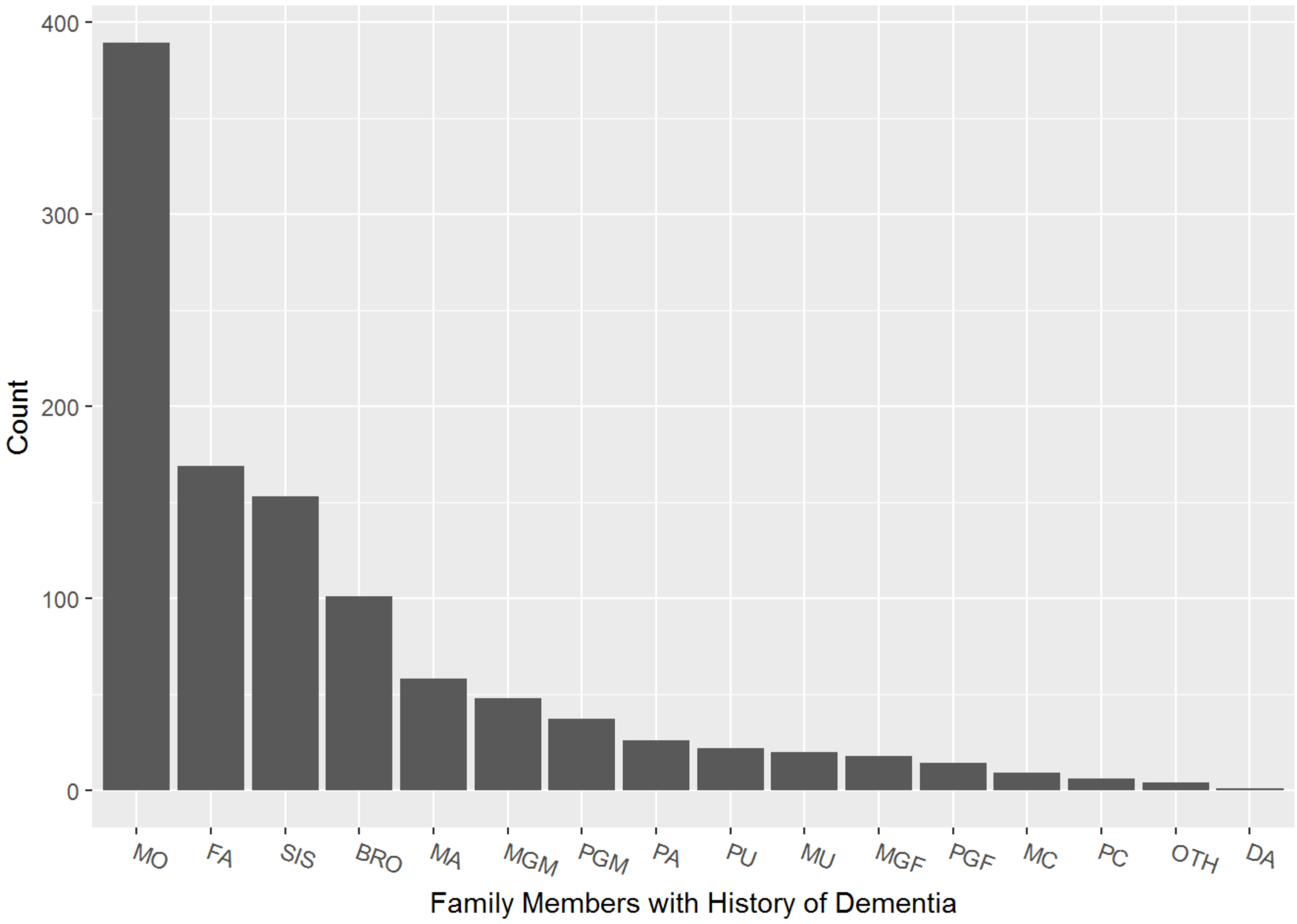

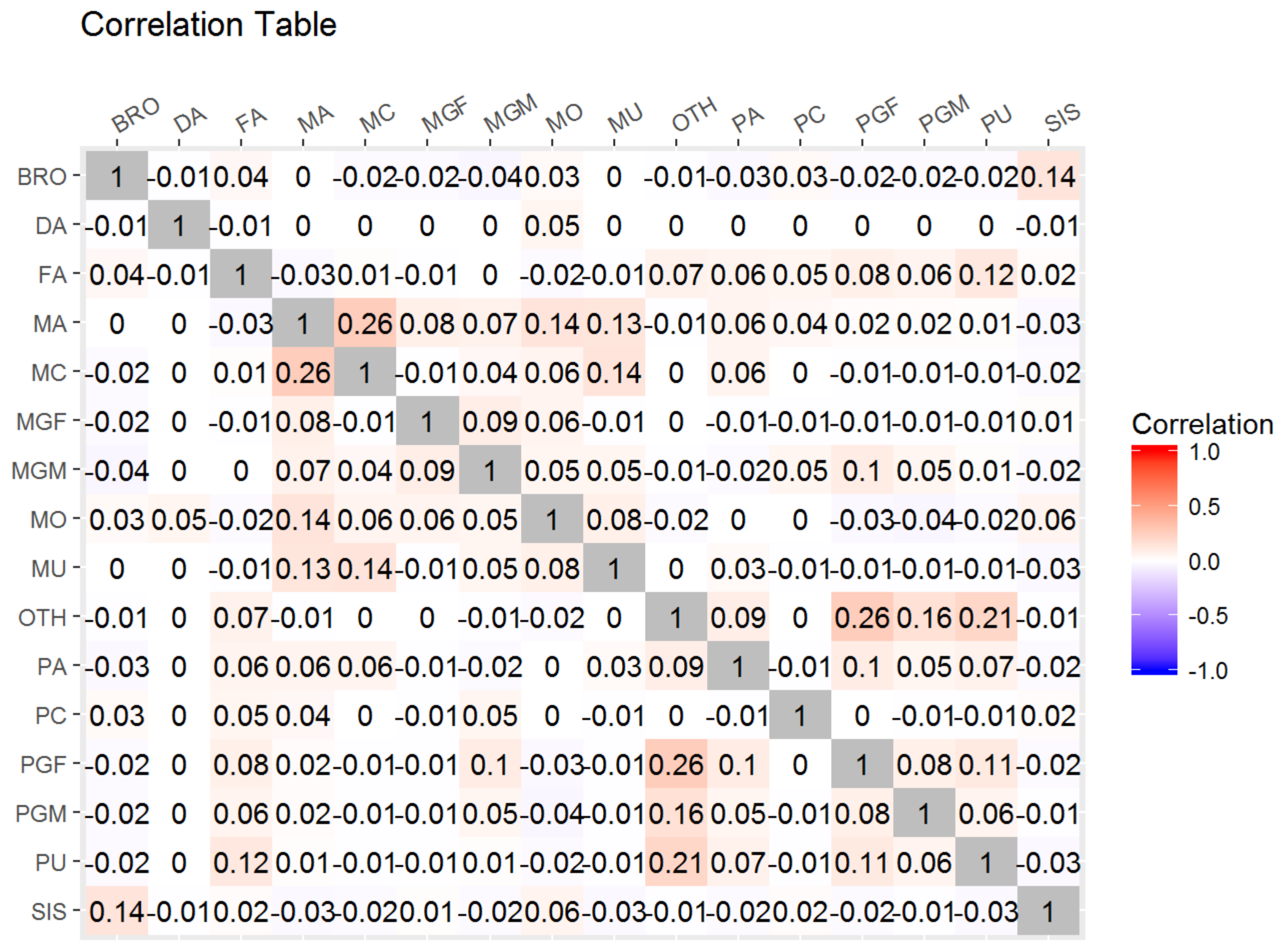

Family History of Parkinsons

Value	Percent	Count	Male	Female
No	94.7	1852	772	1080
Yes	5.3	104	44	60

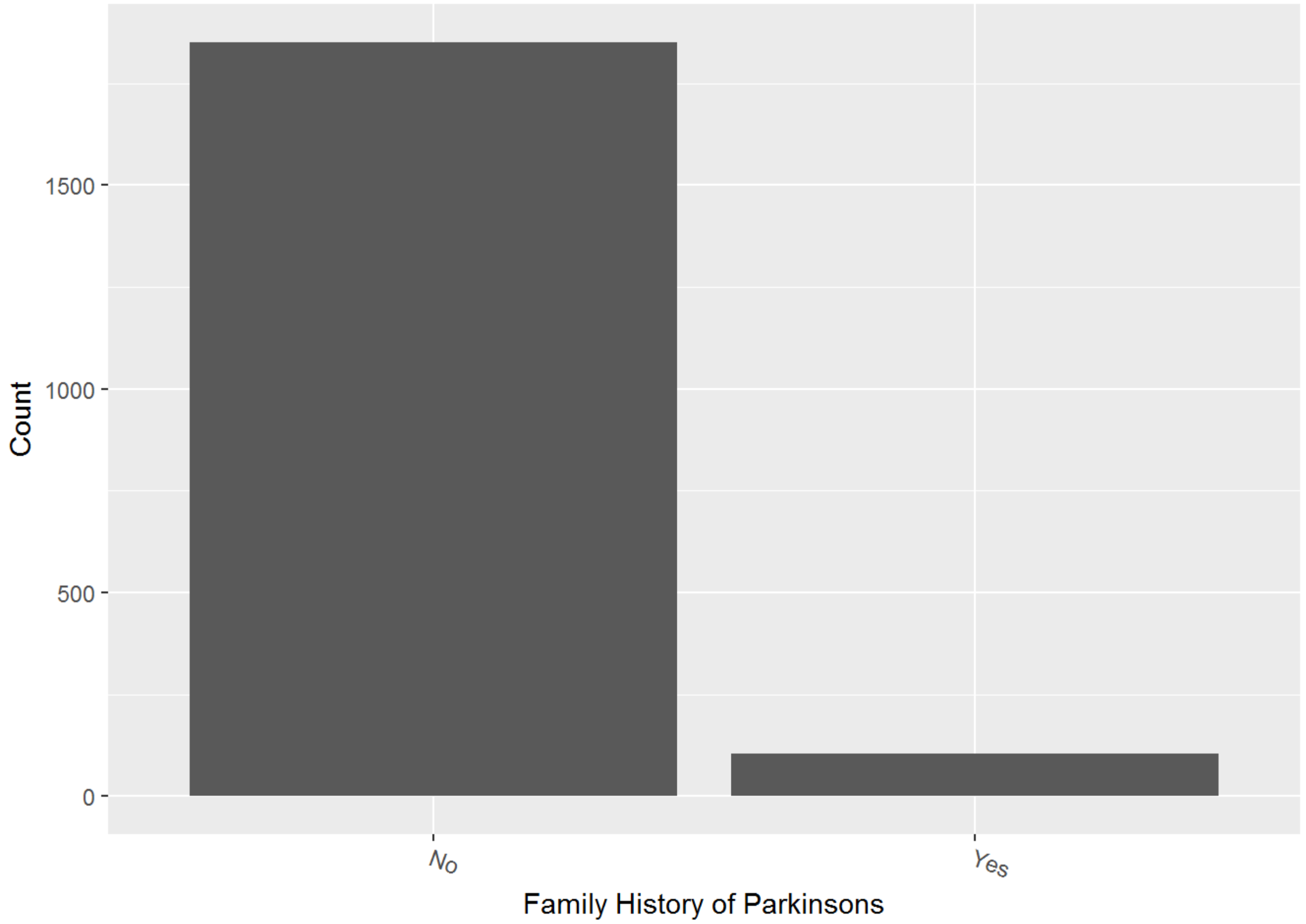

Family Members with History of Parkinsons

Value	Percent	Count	Male	Female
BRO	1.2	24	13	11
FA	1.1	22	7	15
MO	1.1	21	8	13
SIS	0.8	15	8	7
MGM	0.3	6	1	5
PU	0.3	5	2	3
MC	0.1	2	1	1
MGF	0.1	2	1	1
MU	0.1	2	2	0
OTH	0.1	2	0	2
PC	0.1	2	2	0
PGF	0.1	1	1	0
PGM	0.1	2	0	2
SN	0.1	1	0	1

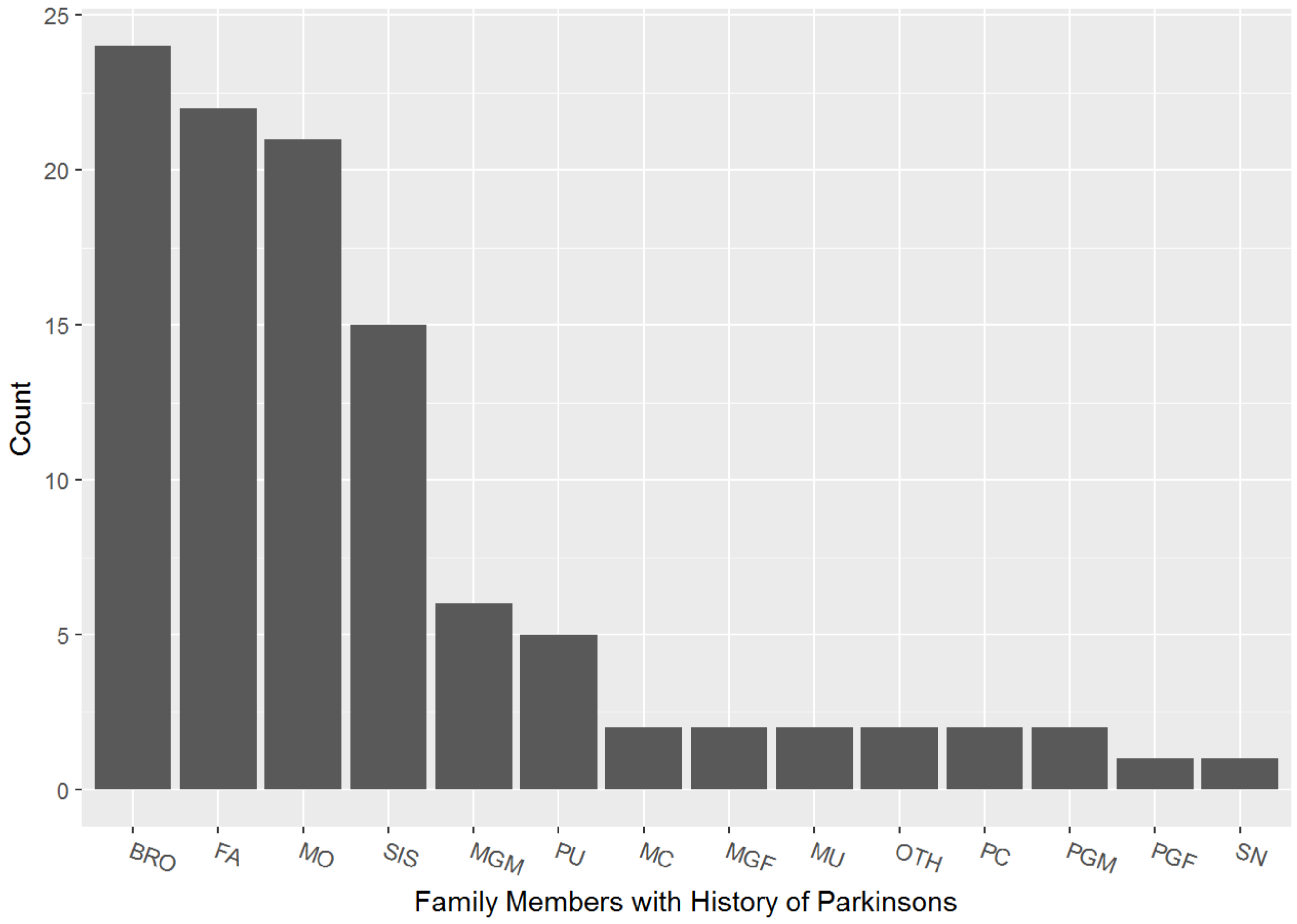

Correlation Table

	BRO	FA	MC	MGF	MGM	MO	MU	OTH	PC	PGF	PGM	PU	SIS	SN
BRO	1	0.03	0	0	0.08	-0.01	0	0	0	0	0	-0.01	0.04	0
FA	0.03	1	0	0	-0.01	-0.01	0	0	0	0	0	-0.01	-0.01	0
MC	0	0	1	0	0	0	0	0	0	0	0	0	0	0
MGF	0	0	0	1	0	0	0	0	0	0	0	0	0	0
MGM	0.08	-0.01	0	0	1	-0.01	0	0	0	0	0	0	0	0
MO	-0.01	-0.01	0	0	-0.01	1	0	0	0	0	0	-0.01	-0.01	0
MU	0	0	0	0	0	0	1	0	0	0	0	0	0	0
OTH	0	0	0	0	0	0	0	1	0	0	0	0	0	0
PC	0	0	0	0	0	0	0	0	1	0	0	0	0	0
PGF	0	0	0	0	0	0	0	0	0	1	0	0	0	0
PGM	0	0	0	0	0	0	0	0	0	0	1	0	0	0
PU	-0.01	-0.01	0	0	0	-0.01	0	0	0	0	0	1	0	0
SIS	0.04	-0.01	0	0	0	-0.01	0	0	0	0	0	0	1	0
SN	0	0	0	0	0	0	0	0	0	0	0	0	0	1

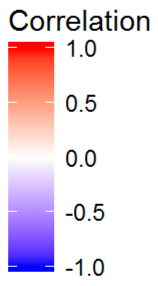

Family History of Stroke

Value	Percent	Count	Male	Female
No	81.1	1587	676	911
Yes	18.9	369	140	229

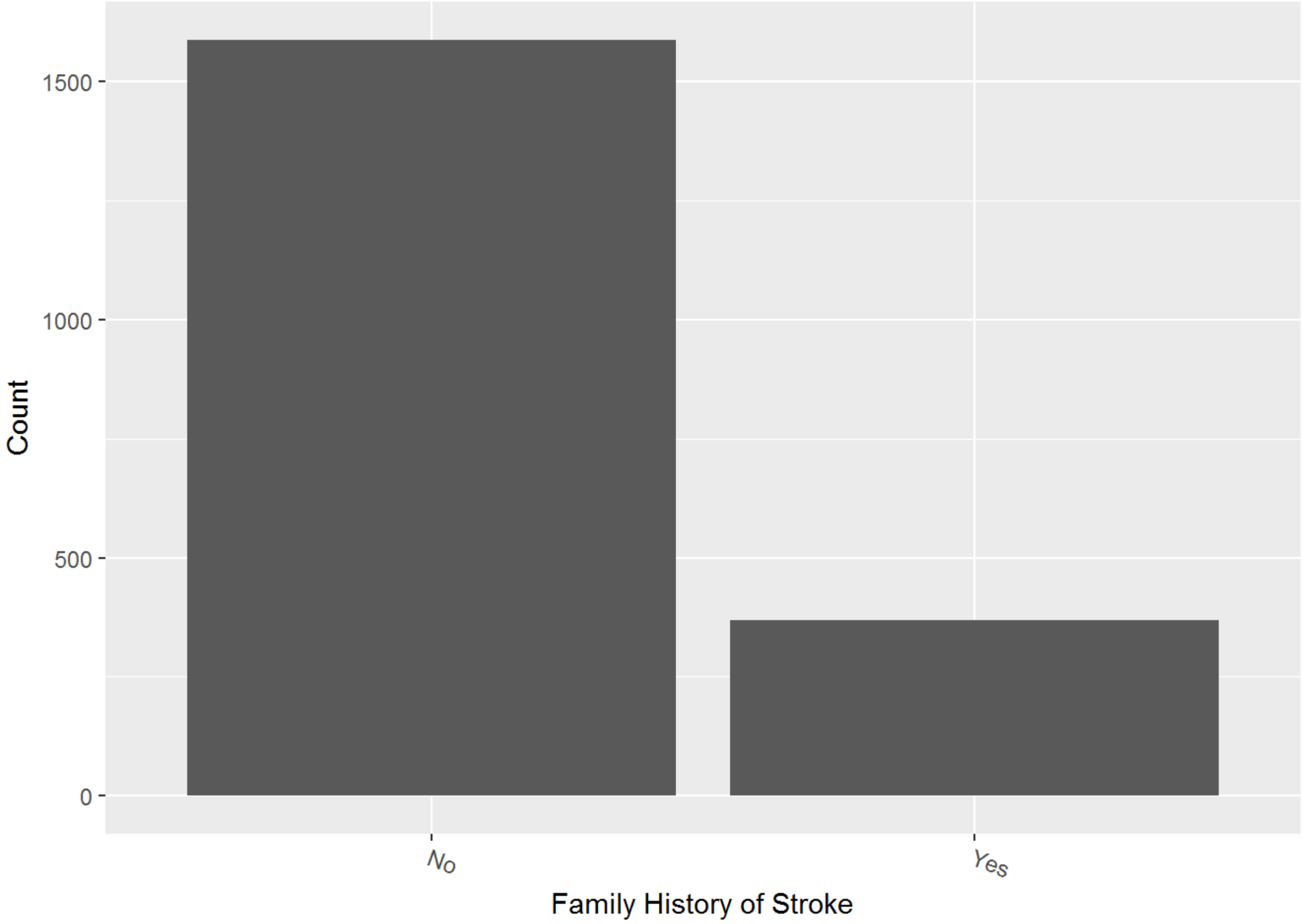

Family Members with History of Stroke

Value	Percent	Count	Male	Female
MO	8.8	172	56	116
FA	7.7	151	70	81
BRO	1.7	33	18	15
SIS	1.6	32	10	22
MGM	0.7	13	4	9
PGM	0.4	7	1	6
MA	0.3	6	3	3
MGF	0.3	6	0	6
PU	0.3	5	2	3
SN	0.3	5	0	5
OTH	0.2	3	1	2
PA	0.2	3	0	3
PGF	0.2	3	0	3
DA	0.1	1	1	0
MU	0.1	2	1	1

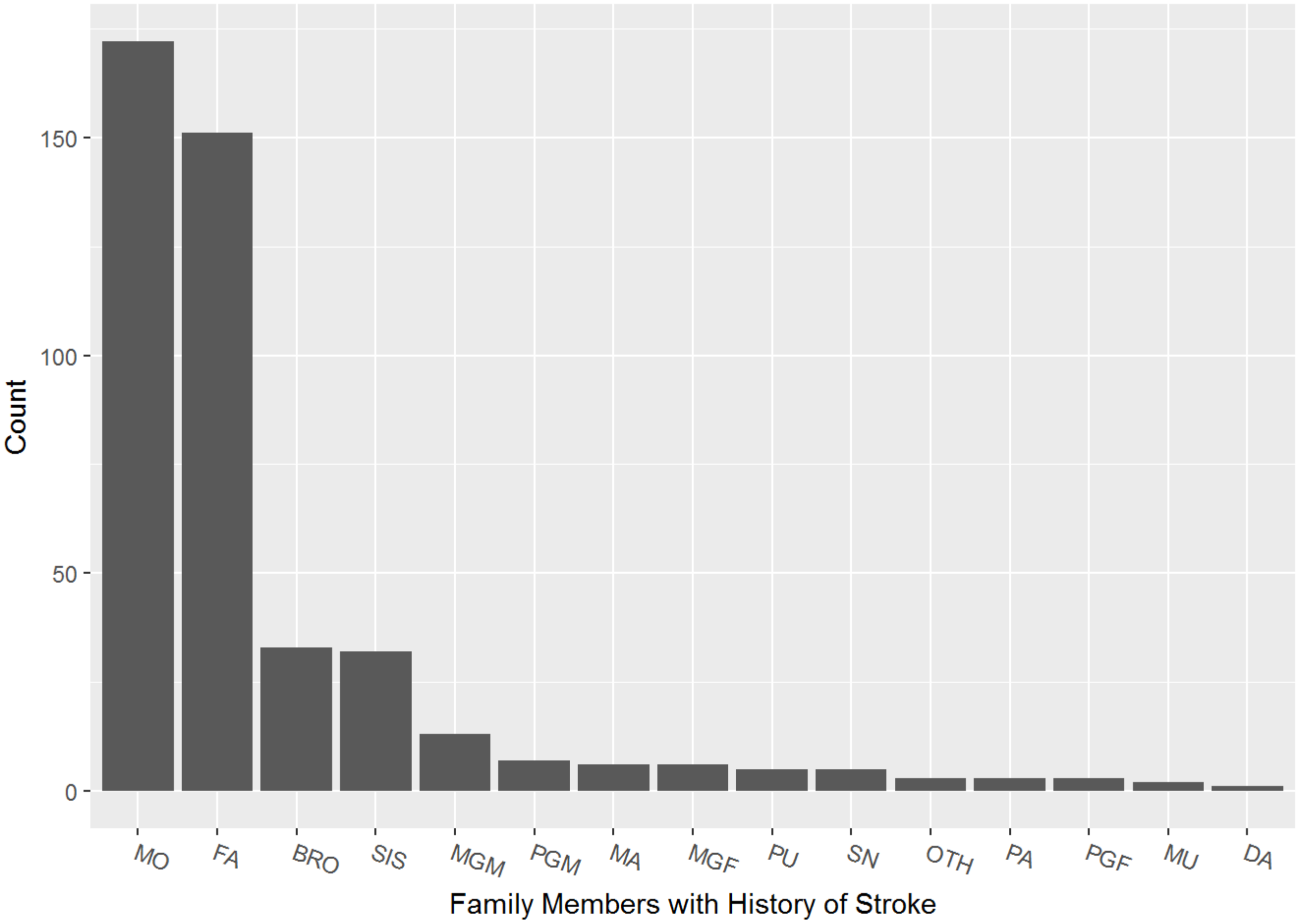

Correlation Table

	BRO	DA	FA	MA	MGF	MGM	MO	MU	OTH	PA	PGF	PGM	PU	SIS	SN
BRO	1	0	0.05	-0.01	0.06	-0.01	0.03	0	-0.01	-0.01	-0.01	-0.01	-0.01	0.08	-0.01
DA	0	1	-0.01	0	0	0	-0.01	0	0	0	0	0	0	0	0
FA	0.05	-0.01	1	0.02	0.02	0	0.11	-0.01	-0.01	0.04	0.04	0.01	-0.01	0.01	0.06
MA	-0.01	0	0.02	1	0.16	0	0.02	0	0	0	0	0	0	-0.01	0
MGF	0.06	0	0.02	0.16	1	0.11	0.08	0	0	0	0	0	0	-0.01	0
MGM	-0.01	0	0	0	0.11	1	0.06	0	0	0	0	0.1	0	0.04	0
MO	0.03	-0.01	0.11	0.02	0.08	0.06	1	-0.01	-0.01	-0.01	0.03	0.01	-0.02	0.07	-0.02
MU	0	0	-0.01	0	0	0	-0.01	1	0	0	0	0	0	0	0
OTH	-0.01	0	-0.01	0	0	0	-0.01	0	1	0	0	0	0	-0.01	0
PA	-0.01	0	0.04	0	0	0	-0.01	0	0	1	0	0	0.26	-0.01	0
PGF	-0.01	0	0.04	0	0	0	0.03	0	0	0	1	0.22	0.26	-0.01	0
PGM	-0.01	0	0.01	0	0	0.1	0.01	0	0	0	0.22	1	0	-0.01	0
PU	-0.01	0	-0.01	0	0	0	-0.02	0	0	0.26	0.26	0	1	-0.01	0
SIS	0.08	0	0.01	-0.01	-0.01	0.04	0.07	0	-0.01	-0.01	-0.01	-0.01	-0.01	1	-0.01
SN	-0.01	0	0.06	0	0	0	-0.02	0	0	0	0	0	0	-0.01	1

Correlation: 1.0, 0.5, 0.0, -0.5, -1.0
