# Supplementary material for: Building of EMR Tools to Support Quality and Research in a Memory Disorders Clinic
Source: Front Neurol. 2019 Mar 7;10:161. doi: 10.3389/fneur.2019.00161 (PMC6416163; doi:10.3389/fneur.2019.00161)
Supplement: Supplementary file 2 [file Data_Sheet_2.PDF]

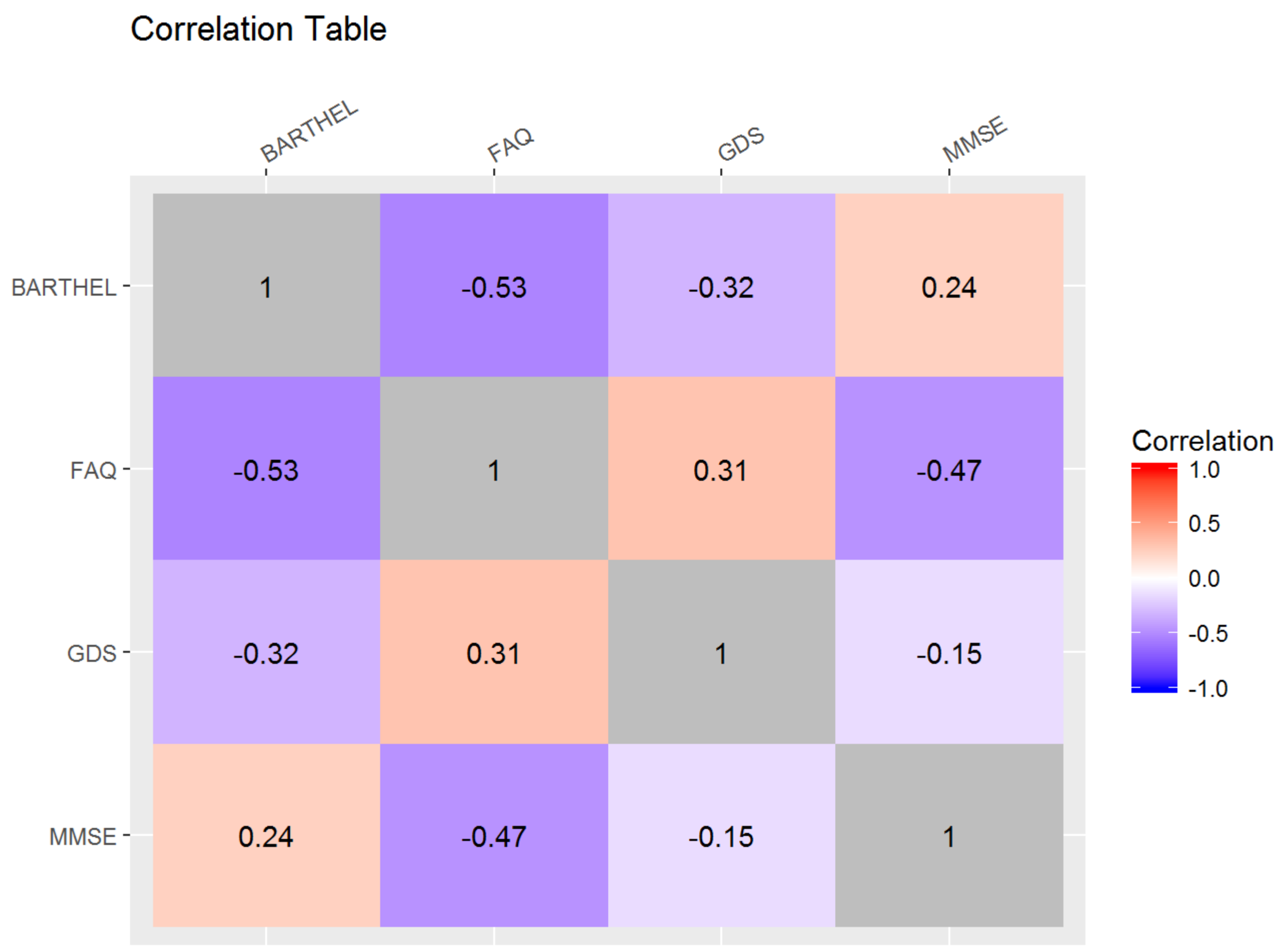

Principal Components, full sample

Total Patients with full data = 1593

Explained Variance

Principal components space can only be explored for complete data, N = 1593.

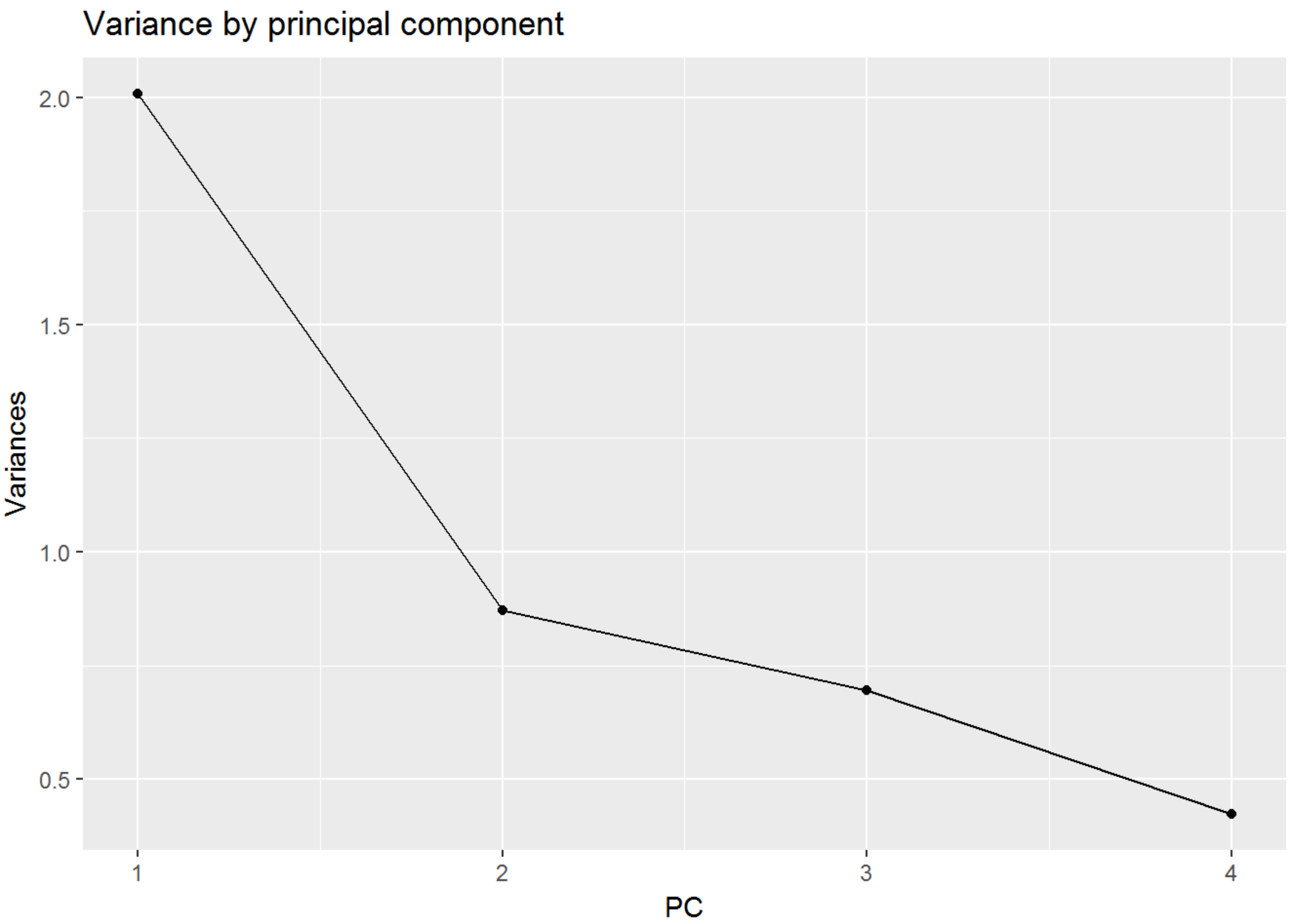

	PC1	PC2	PC3	PC4
**Standard deviation**	1.417	0.9336	0.8347	0.6505
**Proportion of Variance**	0.5021	0.2179	0.1742	0.1058
**Cumulative Proportion**	0.5021	0.72	0.8942	1

Component Loadings

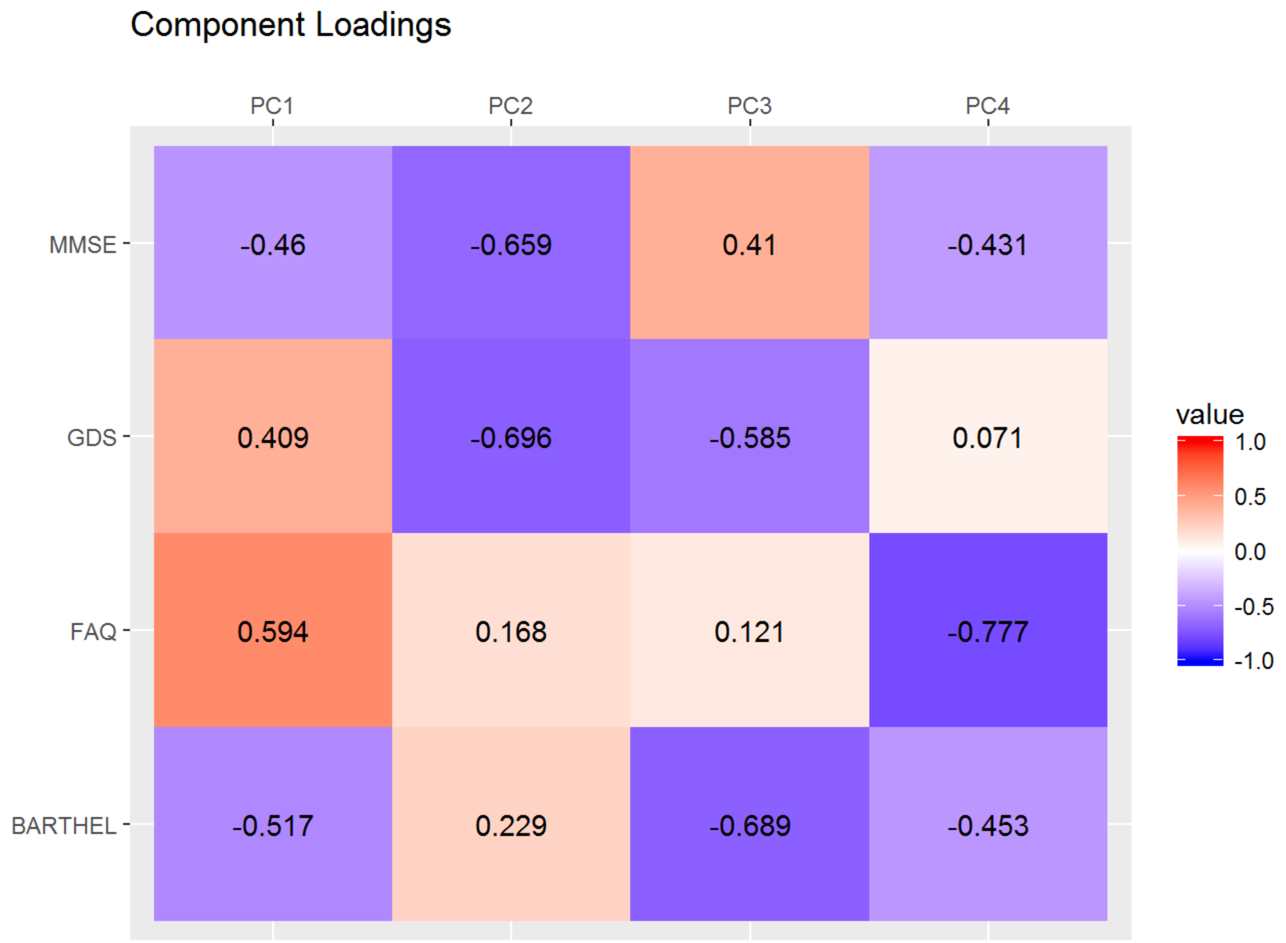

Component and Variable Correlations

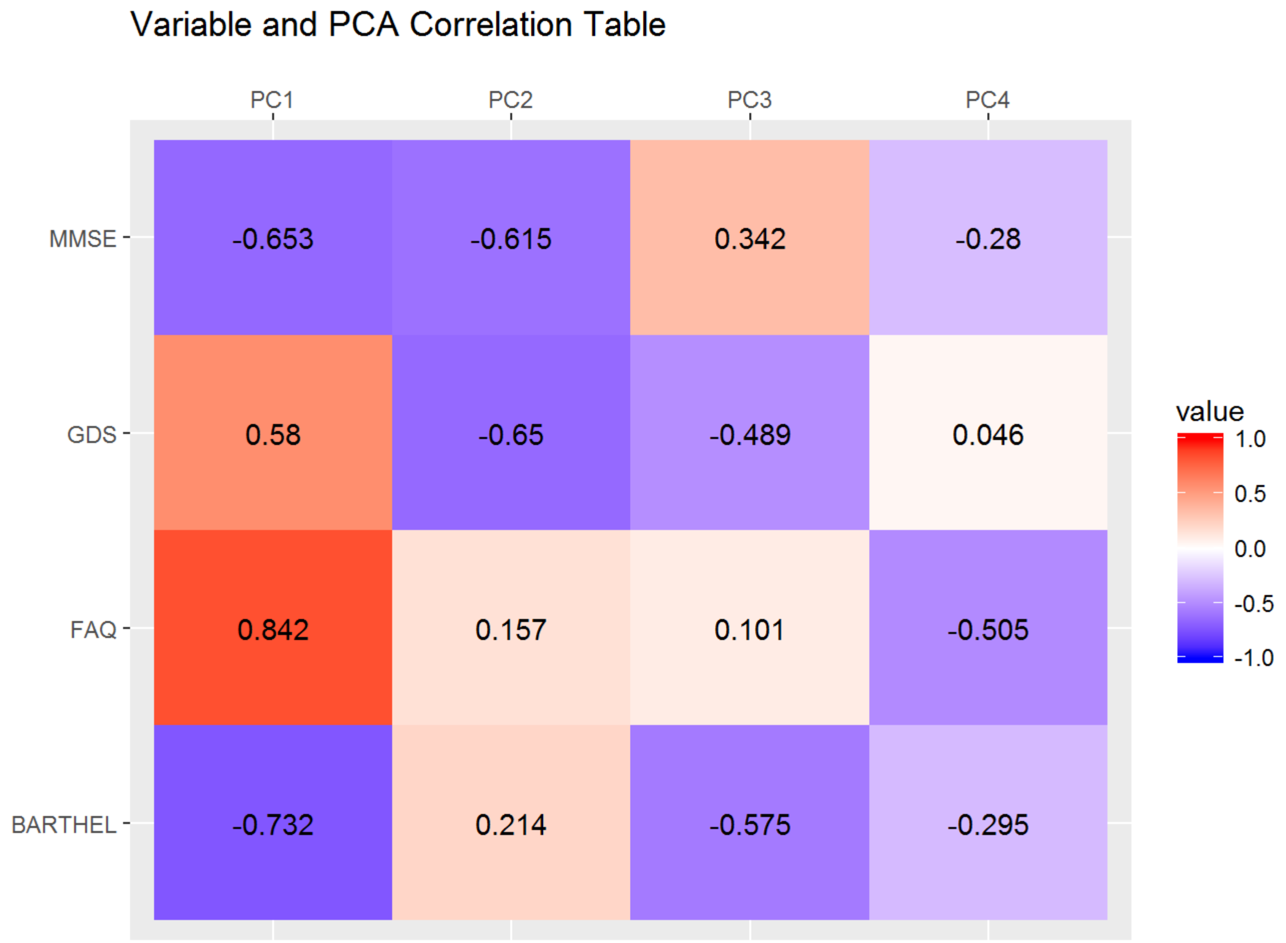

Variable loadings plotted in component space

Variable loadings plotted in component space

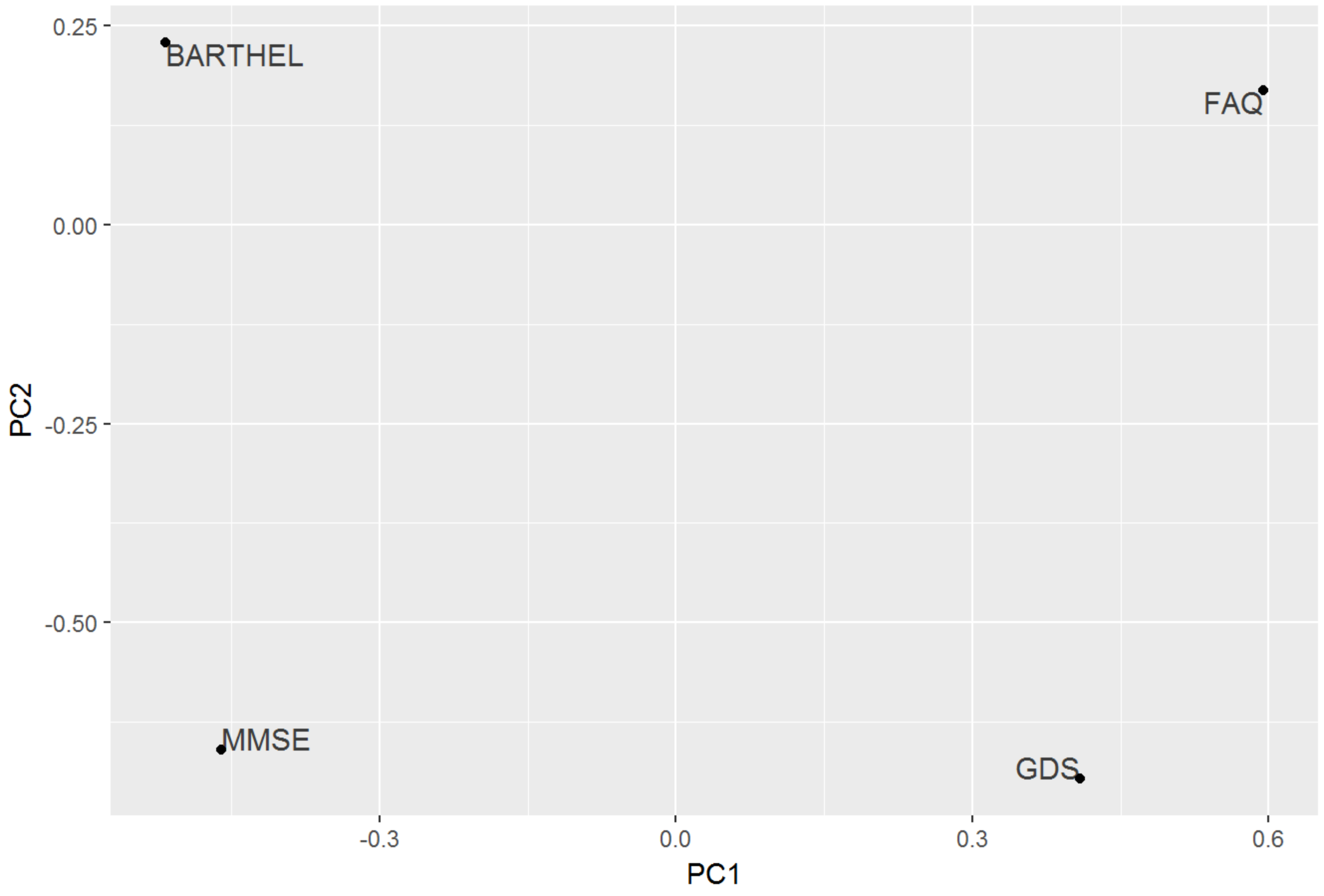

Varimax Rotation

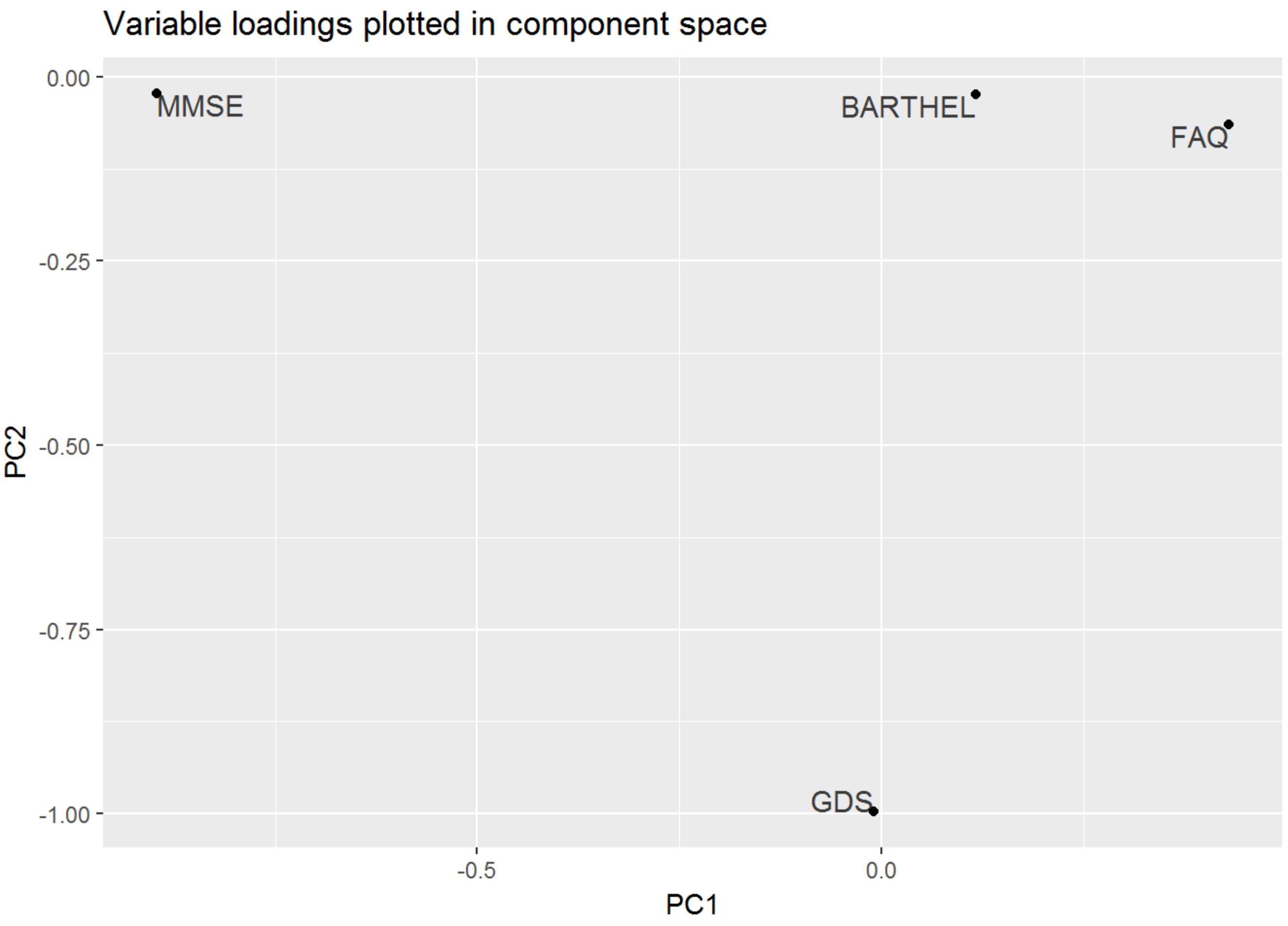

	PC1	PC2	PC3
**BARTHEL_SCORE**	0.1162	-0.02397	-0.8834
**FAQ_SCORE**	0.4283	-0.06491	0.4569
**GDS_SCORE**	-0.01067	-0.9974	-0.01082
**MMSE_SCORE**	-0.8961	-0.02226	0.1039
